# The effects of scientific messages and narratives about vaccination

**DOI:** 10.1371/journal.pone.0248328

**Published:** 2021-03-24

**Authors:** Ozan Kuru, Dominik Stecula, Hang Lu, Yotam Ophir, Man-pui Sally Chan, Ken Winneg, Kathleen Hall Jamieson, Dolores Albarracín

**Affiliations:** 1 Department of Communications and New Media, National University of Singapore, Singapore, Singapore; 2 Department of Political Science, Colorado State University, Fort Collins, Colorado, United States of America; 3 Department of Communication and Media, University of Michigan, Ann Arbor, Michigan, United States of America; 4 Department of Communication, University at Buffalo, State University of New York, Buffalo, New York, United States of America; 5 Department of Psychology, University of Illinois, Champaign, Illinois, United States of America; 6 Annenberg Public Policy Center, University of Pennsylvania, Philadelphia, Pennsylvania, United States of America; South African Medical Research Council, SOUTH AFRICA

## Abstract

A fundamental challenge complicates news decisions about covering vaccine side effects: although serious vaccine side effects are rare, less severe ones do occur occasionally. The study was designed to test whether a side effect message could induce vaccine hesitancy and whether that could be countered by pro-vaccine messages about vaccine safety. A large (*N* = 2,345), nationally representative experiment was conducted by randomly exposing participants to one of six videos about the measles, mumps, and rubella (MMR) vaccine edited from news programs produced during the 2019 measles outbreak in the United States. The design was a 2x3 factorial crossing the presence or absence of a hesitancy-inducing narrative message with a pro-vaccine science-supporting message (i.e., no message, science-supporting expert message, or pro-vaccine narrative message), leading to a total of six conditions. A general linear model was used to assess the effects of these videos on respondents’ (1) vaccine risk perceptions, (2) policy views on vaccination, (3) willingness to encourage others to vaccinate their children, and (4) intention to send a pro-vaccine letter to their state representative. Findings indicated that the science-supporting expert message about vaccine safety led to higher pro-vaccine evaluations relative to other conditions [e.g., b = -0.17, p < .001, a reduction in vaccine risk perceptions of 0.17 as compared to the control]. There was also suggestive evidence that the hesitancy-inducing narrative may limit the effectiveness of a science-supporting expert message, although this finding was not consistent across different outcomes. When shown alone the hesitancy-inducing narrative did not shift views and intentions, but more research is needed to ascertain whether exposure to such messages can undercut the pro-vaccine influence of science-supporting (expert) ones. All in all, however, it is clear that science-supporting messages are effective and therefore worthwhile in combating vaccine misinformation.

## The effects of scientific messages and narratives about vaccination

In 2018–19, the U.S. experienced the largest measles outbreak in over a quarter-century, a disease for which, like COVID-19, an effective and safe vaccine exists [1]. The increasing number of measles cases, largely driven by inadequate vaccination rates in several mostly-religious communities [2], prompted news attention attempting to explain the re-emergence of a disease declared eliminated in the U.S. nearly two decades ago [3, 4]. As cases multiplied, the volume of news coverage, as well as Google searches about measles and the measles, mumps, and rubella (MMR) vaccine, spiked (Supporting Information A in S1 File). Responding to these circumstances, the news media produced stories about the outbreaks, the safety and efficacy of the MMR vaccine, and the reluctance of some in affected communities to vaccinate their children.

Although the media have the potential to educate the public about vaccines, some news practices and values may produce unintended negative consequences. Among these is the journalistic practice that reduces abstract topics, such as vaccination refusal or hesitancy, to personal stories. News accounts about a parent who fears or reports a child’s experience with a side effect cannot be simply debunked as inaccurate or treated as misinformation *in and of itself* [5]. The U.S. Centers for Disease Control reports that the possible risks of the MMR vaccine include (a) soreness, redness, or rash, (b) fever or swelling of the glands in the cheeks or neck, or (c) in more serious cases, seizures, pneumonia, swelling of brain, and unusual bleeding [6]. Unfortunately, because of the availability heuristic [7], news stories about redness or a rash may produce vaccine hesitancy [8] because the audience overgeneralizes the vivid and sometimes dramatic narrative [9] or misjudges the severity of the vaccine side effects.

In this paper, we treated parental reports of potentially real side effects as *Hesitancy-Inducing Narratives* because, even when accurate, their portrayal in media can lead to overgeneralization and fuel vaccine hesitancy by leading the public to draw inaccurate inferences about the prevalence and severity of side effects. In short, individual cases of vaccine side effects, even if true, may elicit false inferences, and the media’s reliance on dramatic and vivid cases may lead to overestimation of risks that are relatively rare. More importantly, individual cases are not the results of randomized control trials (RCTs) conducted during vaccine development and approval processes, and hence do not constitute scientifically valid information about the risk of a vaccine. Hence, they should not undercut the generalized results of RCTs.

This dual aspect of vaccine side effects—their rare existence and people’s tendency to overgeneralize from individual stories—places their portrayal in the media in what we consider a gray zone between accurate and misleading information. We thus posit that media coverage of such stories without proper contextualization can be misleading [10] and has the potential to influence public opinion. Such stories have even influenced decisions by national health organizations as was the case in June 2013 when the “Japanese Ministry of Health, Labour, and Welfare suspended proactive recommendations for the HPV vaccine after unconfirmed reports of adverse events following vaccination appeared in the media” [11].

### Influence of vaccine hesitancy-inducing narratives

Even though prior experimental work has shown that visiting websites that are misleadingly critical of hypothetical vaccines can increase vaccine risk perceptions [12], no prior work has established whether media coverage of stories about side effects also induces (or reinforces) vaccine hesitancy. However, the existing literature offers important hints. Specifically, a number of narrative biases explain how individual accounts of vaccine side effects could be more influential than statistical information about their prevalence [13–15]. For example, narrative information about side effects leads to lower intentions to vaccinate than does statistical information about them [13].

Past research on narrative bias has shown that curbing the influence of narratives is often difficult and finding ways of reducing this bias is important [5]. Previous work on individual stories of vaccine side effects has focused on the effects of the type of information (statistical or narrative) about side effects on vaccine beliefs and intentions but has not established how best to counteract the negative influences of messages about them. Thus, our aim was to examine whether stories of vaccine side effects may be counteracted by other news intended to convey the safety and benefits of vaccination. Studies of the positive [16–18] and counter-productive [19–21] effects of attempts to reduce misinformation have generally concluded that correcting misinformation is challenging [13, 14]. Moreover, by eliciting more thinking about the earlier misinformation, corrections can make it more familiar and accessible [22]. In that situation, corrections can amplify the initial effect of the misinformation, causing message recipients to be more misinformed after than before the correction. In other cases, simply providing correct information fails to counter the emotional effects of misinformed narratives [5]. Indeed, a meta-analysis found that the misinformation effects are, on average, much larger than the debunking ones [22]. Taken together, studies on misinformation have revealed that corrections do not necessarily remove misperceptions and misinformation and that their effectiveness depends on individual differences in motivation and educational level [19, 20, 23, 24].

More specifically, hesitancy-inducing narratives may be counteracted with two types of correction strategies: (a) statistical information that is usually delivered by experts or (b) narratives about people who are pro-vaccine and share their experiences. Expert-delivered statistical information is typically effective at changing beliefs, attitudes, and behaviors [17, 18, 25] but can sometimes backfire [26]. Correctives that use emotional personal narratives are often successful as well [5, 27–29], in part because misinformation persistence is often linked to affect [30], especially when embedded in narratives [5]. Yet, how these interventions operate in individuals’ processing of vaccine side effect stories in the media remains to be established.

The context in which the public encounters messages that induce hesitancy could create additional challenges. One of these contextual matters is whether an audience receives pro-vaccine messages only or sees them juxtaposed with hesitancy-inducing narratives. Prior work on the process of correction has compared the standalone misinformation with the misinformation followed by correct information using either a between- or within-subjects design [22]. Examining such combined messages is important because an audience that receives a hesitancy-inducing narrative followed by a claim that the vaccine is safe might process the second claim differently than would those exposed only to the narrative or to the correct information [31, 32].

Similarly, information presented as categorical (e.g., “the vaccine is safe”) can be refuted by a single contrary example (of a real side effect). In such a circumstance, general and statistically supported claims about the safety of vaccines delivered by an expert such as Dr. Fauci may lose their effectiveness. Finally, examining combined messages is important because they better reflect news ecosystems in which people are exposed to conflicting messages due to what is known as media “false-balance” [31]. It is also important to examine epistemologically different forms of scientific information (e.g., aggregate conclusions drawn from randomized placebo-controlled trials on vaccine safety) vs. individualized narratives about side effects.

### Current study

This study examined the individual and combined effects of exposure to a news account of a parent’s story of a child who experienced a negative vaccine reaction and news accounts about the value and safety of the MMR vaccine during the 2018–2019 U.S. measles outbreak. We relied on a between-subjects factorial experimental design. Our factorial approach manipulated 1) absence or presence of the hesitancy-inducing narrative message (a message describing vaccine side effects) and 2) presence of an expert pro-vaccine science message containing statistical information, a narrative pro-vaccine message from the point of view of parents, or no pro-vaccine message. This 2x3 factorial design allowed us to compare a total of six conditions. It also allowed us to test both the effects of hesitancy-inducing narratives and those of correction strategies on vaccine views and intentions. Analyses thus included main effects and the interaction between the two factors.

Although we utilized a factorial approach in the analysis, we formulated specific expectations and research question as well, which were preregistered. First, we hypothesized that exposure to the hesitancy-inducing narrative compared to the control would yield (a) higher perceptions of vaccine risk, (b) less support for pro-vaccine policy positions, (c) weaker intentions to encourage parents to vaccinate their children, and (d) lower probability of agreeing to send a pro-vaccine letter to a state representative **(H1**). Second, exposure to either the science-supporting (statistical) message or the science-consistent narrative would yield lower perceptions of vaccine risk, more support for pro-vaccine policies, stronger intentions to encourage parents to vaccinate, and higher probability of agreeing to send a pro-vaccine letter to a state representative than would the control message and the hesitancy-inducing narrative **(H2).** Third, both combined conditions would produce lower perceptions of risk, higher levels of pro-vaccine policy support, stronger intentions to encourage parents to vaccinate their children, and higher probability of agreeing to send a letter to a state representative than would the hesitancy-inducing narrative alone **(H3**). Finally, we compared the influence of pro-vaccine messages (both the science-supporting message and the science-consistent narrative) with and without the initial presentation of the hesitancy-inducing narrative (**RQ1).** This comparison allowed us to test for the influence of combined messages that include both hesitancy-inducing and pro-vaccine elements.

Our study also makes a novel contribution by focusing on the influence of coverage of vaccine side effect stories in the context of ecologically valid, real news videos. By contrast, most of the prior work in this domain focused on fictitious diseases and hypothetical vaccines and did not characterize public reaction to *actual* information about the MMR vaccine. In addition, we investigate effects on a variety of attitudinal and intentional outcome measures.

## Methods

### Data

We collected data through the *AmeriSpeak* panel of NORC at the University of Chicago. *AmeriSpeak* is a probability-based nationally representative sample of American adults (see Supporting Information B in S1 File). The NORC recruitment panel was a two-stage stratified sampling frame that covers 97% of U.S. households. To prevent sampling bias (e.g., some parents might be more interested in the topic of vaccine), the invitation did not indicate that the study would focus on vaccination. They were invited to “a survey about health and wellness.” More details about the NORC sampling procedures are provided in Supporting Information B in S1 File.

Two different nationally representative samples were collected concurrently between February 28 and March 18, 2019. We combined one from an ongoing longitudinal panel on vaccination (N = 1,682), and a supplemental, cross-sectional sample (N = 864) for a total of 2,546 random assignments (see the exclusion procedures section below for the effective sample size). This sampling procedure helped us achieve the appropriate sample size determined by the power analysis (see Supporting Information B in S1 File). The demographic composition of the samples as well as comparisons to the latest U.S. Census estimates also appear in Supporting Information B in S1 File.

The first sample was part of a larger panel study of infectious diseases and vaccination attitudes focusing on the flu, measles-mumps-rubella, and human papillomavirus. The experimental module was within the fourth wave of the panel and focused on the measles to make it possible to study the outbreak occurring in the country at the time of data collection. The number of participants was 1,803. Most responded to the survey over the internet (N = 1,682, 93.3%), although a smaller group did so over the phone (N = 121, 6.7%). Among the Internet respondents, 766 took the survey on a desktop computer (42.5%), 807 (44.8%) on a smartphone, and 109 on a tablet (6%). The retention from Wave 1 (September 2018) to Wave 4 (March 2019) was 60% (exactly).

The second sample was collected by the same survey company and is made up of 1,006 “fresh” respondents who were unrelated to the panel sample (to check for the panel effects of the larger study). Most respondents in this new sample also took the survey over the internet (N = 864, 85.9%); a smaller proportion completed it over the phone (N = 142, 14.1%). Because our experiment contained audio-visual stimuli, those respondents who took the survey on the phone (listening and answering questions vocally) were not part of the experiment. Phone respondents had a slight overrepresentation of older respondents. Among Internet respondents, 324 respondents took the survey over a desktop computer (32.2%), 496 (49.3%) on a smartphone, and 44 on a tablet (4.4%).

### Procedure

After the informed consent, respondents answered a series of pre-treatment questions. In the manipulation section, the news clips were designed to ensure exposure; respondents were not able to fast-forward but were able to re-watch the video if they chose to do so. Also, participants could not move to the next page of the survey without watching the video at least once. After the exposure, we asked another set of quality control questions. We asked whether respondents were able to hear the video. Since we conducted the study shortly after the news clips aired, we also asked them if they had seen the video before (partially or fully). Exclusion criteria and results based on these quality checks are explained below. The outcome measures, manipulation checks, and debriefing of respondents about the experiment followed.

### Manipulations

Participants viewed one of the six videos. The conditions were created to test the potential hesitancy-inducing influence of the side-effects story in relation to a control group, as well as potential countering forces of the statistical or narrative science-supporting messages either presented alone or presented in combination with the hesitancy-inducing narrative message. Real, contemporary news videos were obtained from the websites of NBC, ABC, CBS, and PBS and then professionally edited for length. Videos were stripped of channel logos to minimize source effects. Full details on each video, as well as a link to each news clip, are provided in Supporting Information B in S1 File. A brief description of video contents is provided below.

The first video condition (**C1**), ***hesitancy-inducing narrative (Hes)*,** showed a mother expressing regret about vaccinating her first child against MMR and indicating that she would not vaccinate her second child because her older boy suffered pictured side-effects. The narrator quotes the mother saying that the child suffered severe side effects. In response to the reporter’s question about whether she believes that the CDC. is lying when it says vaccination is safe, the mother asserts that scientists do not know or adequately understand all of the potential hazards of vaccinations. We named this condition hesitancy-inducing narrative because it is a personal story without statistics. The second condition (**C2**), ***science-supporting (statistical) message (SSM)*,** showed an expert, Dr. Anthony S. Fauci, Director of the National Institute of Allergy and Infectious Diseases, providing scientifically based statistical information about the contagiousness of measles and the safety and effectiveness of the vaccine. Because the video includes an expert and statistical information, we named this condition science-supporting (expert) message. In the third condition (**C3**), ***science-consistent narrative (SCN)*,** participants were presented with the stories of several families with children who either would be endangered by exposure to measles (e.g., who cannot get the vaccine because of other health conditions or age limits) or who had caught measles and experienced complications. These parents blamed their situation on those who lowered herd immunity because they refused vaccination. We named this condition the science-consistent narrative because its personal stories deliver pro-vaccine information without using pro-vaccine statistics. In the fourth condition (**C4**), ***hesitancy-inducing narrative + science-supporting statistical message (Hes+SSM)*,** participants watched the hesitancy-inducing narrative followed by the science-supporting message. The two components of this combined video were slightly shortened to make the length comparable with the first three conditions. The two parts are presented uninterrupted in a single continuous video. In the fifth condition (**C5**), ***hesitancy-inducing narrative + science-consistent narrative (Hes+SCN)*,** participants watched the hesitancy-inducing narrative followed by the science-consistent narrative. The two components of this combined video were slightly shortened as well and the two parts are presented uninterrupted in a single continuous video. Finally, participants in the **control condition** (**C6**) watched a video of comparable length about aspirin. This video was edited to be equivalent in length and format and addressed a health-related topic not related directly to vaccines. These conditions were categorized based on the 2x3 factorial design as mentioned before.

### Exclusion procedures

Not all participants from the sampling procedure were included in the experiment. Since the study was conducted online using a self-administered survey experiment with video messages, we administered the manipulations carefully to ensure exposure. One hundred twenty-five respondents, homogenously distributed across conditions, were excluded for one of three reasons: not being able to hear the audio check, reporting having been exposed to the video in full outside of the study, or not being able to play the experiment video: (1) 85 reported they could not hear, and others entered the wrong number during an audio check at the beginning of the study. The 85 participants who could not hear were asked if they had another audio-working device. Of those 85, the 64 who said they did not were dropped from the experiment; others joined with a new device. (2) After viewing the video, 32 respondents stated that they had seen it fully somewhere and were thus dropped from analysis because prior exposure would confound our experimental manipulation. However, 80 respondents who said they remembered seeing part of the video were retained. (3) 22 participants indicated they could not view the entire video and were dropped from analysis due to inadequate exposure to the experimental manipulation. (4) 7 participants who indicated they could not hear the experiment video were dropped as well due to inadequate exposure to the experimental manipulation. Additionally, those who left the audio check question blank (N = 56) at the very beginning of the survey were excluded.

Due to a survey progression (display logic) error, some participants were able to answer questions without having been shown the experimental module; this reduction in sample size was also random across conditions. Hence, the overall experiment sample size was 2,345. A small number of participants with missing data in dependent and independent variables were also dropped. Final Ns are reported along with the results in Table 1.

**Table 1:** General linear model, fixed effects ANCOVA and binary logit models predicting four outcome variables with experimental conditions and covariates.

	MMR Vaccine Risk Perceptions		Pro-Vaccine Policy Views		Encouraging Others to Vaccinate Their Children				Agreeing to Send a Pro-Vaccine Letter	
					Coding 1		Coding 2			
	*GLM*	*ANCOVA*	*GLM*	*ANCOVA*	*Logistic*	*OR [CI]*	*Logistic*	*OR [CI]*	*Logistic*	*OR [CI]*
	*Coef*. *(se)*, *p*	*F (1*, *2280)*,	*Coef*. *(se)*, *p*	*F (1*, *2285)*,	*Coef*. *(se)*, *p*		*Coef*. *(se)*, *p*		*Coef*. *(se)*, *p*	
		*p*, *(η*_*p*_^*2*^*)*		*p*, *(η*_*p*_^*2*^*)*						
Intercept	2.38*** (.07)	-	3.00*** (.13)	-	-1.68*** (.37)	-	-2.99*** (.40)	-	-1.22*** (.36)	-
Hesitancy-Inducing Message Factor	.02 (.03)	.44 (.000)	-.11* (.06)	3.96* (.002)	.02 (.19)	1.0 [.7–1.5]	.07 (.17)	1.1 [.8–1.5]	-.15 (.16)	.9 [.6–1.2]
Science-Supporting Message Factor	-	17.26*** (.015)		13.45*** (.012)	-	-	-	-	-	-
Science-Supporting Message	-.17*** (.03)	-	.18*** (.05)	-	.52** (.19)	1.7 [1.2–2.5]	.52*** (.17)	1.7 [1.2–2.3]	.37* (.16)	1.5 [1.1–2]
Science Consistent Narrative	-.02 (.03)	-	-.10 (.08)	-	.13 (.19)	1.1 [.8–1.7]	-.02 (.17)	.9 [.7–1.4]	-.16 (.16)	.9 [.6–1.22]
Hesitancy-Inducing Message Factor X Science-Supporting Message Factor	-	3.05* (.003)		3.21* (.002)	-	-	-	-	-	-
Hesitancy-Inducing Message X Science Supporting Message	.07 (.04)	-	.00 (.08)	-	-.34 (.28)	.7 [.4–1.2]	-.49* (.24)	.6 [.4–1]	-.01 (.23)	.99 [.6–1.6]
Hesitancy-Inducing Message X Science Consistent Narrative	-.04 (.05)	-	.17* (.08)	-	-.11 (.27)	.9 [.5–1.5]	.08 (.24)	1.1 [.7–1.7]	.48* (.23)	1.6 [1–2.5]
			***Covariates***							
Vaccine Misinformation	.47*** (.02)	731.25*** (.243)	-.66*** (.03)	471.16*** (.171)	-.84*** (.10)	.4 [.3-.5]	-1.37*** (.11)	.3 [.2-.3]	-.93*** (.09)	.4 [.3-.5]
Media Exposure (Measles)	-.06*** (.01)	38.8*** (.017)	.07*** (.02)	21.04*** (.009)	.30*** (.06)	1.4 [1.2–1.5]	.29*** (.05)	1.2 [1.1–1.5]	.17*** (.05)	1.2 [1.1–1.3]
Trust in Medical Authorities	-.29*** (.02)	316.2*** (.122)	-.23*** (.03)	60.8*** (.028)	.83*** (.09)	2.3 [1.9–2.7]	.86*** (.10)	2.4 [2–2.9]	.37*** (.08)	1.5 [1.2–1.7]
Ideology (Liberal)	-	-	.09*** (.01)	37.45*** (.016)	-	-	-	-	.21*** (.04)	1.2[1.1–1.3]
Parent	.03 (.02)	3.37 (.001)	-.05 (.03)	2.35 (.001)	.14 (.11)	1.2 [.9–1.4]	-.08 (.10)	.9 [.8–1.1]	-.10 (.09)	.9 [.8–1.1]
Female	.00 (.02)	0.002 (.000)	.00 (.02)	6.28** (.003)	.55*** (.12)	1.7 [1.4–2.2]	.43 *** (.10)	1.5 [1.3–1.9]	-.10 (.09)	.9 [.8–1.1]
Supplemental Sample	.03 (.02)	2.77 (.001)	-.02 (.03)	.42 (.000)	.06 (.12)	1.1 [.8–1.4]	-.02 (.11)	.9 [.8–1.1]	-.15 (.10)	.9 [.7–1.1]
***R***^***2***^	.54		.39		.19		.23		.13	
***N***	2,292		2,298		2,342		2,342		2,305	

**Notes.** For MMR vaccine risk perceptions and pro-vaccine policy views, we report coefficients from general linear model (GLM) as well as analysis of covariance (ANCOVA) results. For encouraging others to vaccinate their children and agreeing to send a pro-vaccine letter to state representative, modelled with binary logistic regressions, coefficients (log odds) and odds ratios are reported. Standard errors are in parentheses and confidence intervals are in brackets. OR–odds ratio, *η*_*p*_^*2*^ *–*partial eta square. For encouraging others to vaccinate their children, results for two different coding strategies are reported (see Methods section). Significance tests rely on t value for GLM, F value for ANCOVA, and z scores for binary logit regression. ANCOVA p values are Type III sum of squares.

*** is p < .001,

** is p <. 01,

* is p < .05. For GLM/logit, factor coding was as follows: Hesitancy Absent = 0, Hesitancy Present = 1, Science-Supporting Message Factor reference is the Control condition. Hence, reference group is the control (Hesitancy Absent + Science-Supporting Message Null). Ideology is only included in policy-relevant models. For logistic models, McFadden pseudo-R^2^ are reported. Model fit for Likelihood of Encouraging Others to Vaccinate (MMR) Their Children (C1: Coding 1, see Methods section for details of coding): χ2(11) = 468.313, p< .001 (log-likelihood = -997.9179). Model fit for Likelihood of Encouraging Others to Vaccinate (MMR) Their Children (C2: Coding 2, see Methods section for details of coding): χ2(11) = 709.006, p< .001 (log-likelihood = -1237.35). Model fit for Willingness to Send a Pro-Vaccine Letter to State Representative: χ2(12) = 417.714, p< .001 (log-likelihood = -1362.521).

### Manipulation checks

Manipulation checks asked respondents to identify whether any of the eight listed content items appeared in the video. Four of the items (individually or in different combinations) were included and the remaining four were distractors that did not appear in any of the conditions. This arrangement allowed us to assess how well the manipulations worked across conditions. The results showed that our manipulations worked. (For details, see Supporting Information B in S1 File).

### Ethics statement

The study received approval from the Institutional Review Board (IRB) of the University of Pennsylvania. The study team declares full compliance with the ethical guidelines. Participants were appropriately asked for informed consent about the study at the beginning and they were debriefed at the end. The study participants were told that they could contact the research team with questions or if they wanted to see the original full versions of the news clips. Note that the videos they were shown were edited versions of news segments that had been nationally aired a few weeks before the study. Our study prioritized using actual news videos and not manipulating their content such that no information was changed or added during an epidemic at the time of data collection. The only stylistic change added a print overlay for the statistical information offered by Dr. Fauci. Hence, we did not expect or encounter adverse conditions or ethical problems, and there was no deception.

### Measures

Outcome measures included evaluations of the MMR vaccine risk perceptions, pro-vaccine policy views, encouraging others to vaccinate their children, and agreeing to send a pro-vaccine letter to state legislators. Full question and response option wordings for all questions are provided in Supporting Information B in S1 File.

#### MMR vaccine risk perceptions index (outcome measure)

We measured MMR vaccine evaluations with four items that tapped various but closely related beliefs and evaluations about the vaccine, which were averaged into an index (e.g., “Just your best guess, how risky, if at all, do you think the measles, mumps, and rubella (MMR) vaccine is?” with response options, “Not risky at all,” “Not too risky,” “Somewhat risky,” “Very risky,” Supporting Information B in S1 File). The items had high reliability (α = .81) and an exploratory factor analysis suggested a single factor solution was reasonable (Maximum-Likelihood estimation, variance explained = .54). Higher scores in the index represent more negative evaluations of the MMR vaccine. Given the subjective nature of risk perceptions and variety of ways to measure the construct [33], tapping these closely related vaccine evaluations with multiple items provides broader applicability and ecological validity for our conclusions about vaccine risk perceptions and prevents bias from a single item. We also provide the main results for each item separately in Supporting Information C in S1 File.

#### Pro-vaccine policy index (outcome measure)

Pro-vaccine policy views were measured with six items about ongoing policy discussions around various types of exemption to vaccination [34]. To capture the different dimensions of the policy debates surrounding vaccination, such as forms of exemption (e.g., religious or medical) and mandatory school vaccinations, we asked these multiple questions, such as “It should be mandatory for parents to vaccinate their children against preventable diseases such as measles, mumps, and rubella,” with response options “Strongly support,” “Somewhat support,” “Neither support nor oppose,” “Somewhat oppose,” “Strongly oppose,” Supporting Information B in S1 File). The items had high reliability (α = .85) and an exploratory factor analysis supported a single factor solution (Maximum-Likelihood estimation, variance explained = .52). The measure, which was the average of all six items, provides a battery of overall vaccine-related policy positions. We also provide the main results for each item separately in Supporting Information C in S1 File.

#### Intention to encourage other parents to vaccinate their children (outcome measure)

A single item measured respondents’ willingness to encourage other parents to vaccinate their children against measles, mumps, and rubella: “How likely are you to encourage parents to give the measles, mumps, and rubella (MMR) vaccine to children?” with response options “Not likely at all,” “Not too likely,” “Somewhat likely,” “Very likely” (Supporting Information B in S1 File). Given that this was a specific behavioral intention regarding the MMR vaccine, a single item was deemed sufficient.

#### Agreeing to send a pro-vaccine letter to state representative (outcome measure)

We also used the single item shown for this specific behavioral intention of agreeing to send a pro-vaccine letter. The short letter statement can be seen in Supporting Information B in S1 File. We imputed the respondents’ state of residence into this statement where it reads “*current state of residence”* to increase engagement, relevance, and realism.

#### Covariates

Our theoretical covariates included (a) respondents’ vaccine misinformation (prior vaccine misinformation could influence reaction to hesitancy-inducing narrative), measured with four items (e.g., “It is better to develop natural immunity by getting the disease than by receiving the vaccine”), and averaged into an index (α = .80); (b) trust in medical authorities (trust could influence reaction to science-supporting or science-consistent messages), measured with two items (“U.S. Centers for Disease Control and Prevention (CDC)” and “Your primary care doctor or primary medical provider”) and averaged into an index (α = .80). We also measured (a) conservative-liberal ideology, which we included as a covariate for policy-related outcomes, with a single item; (b) exposure to news about measles (as an indication of issue salience) measured with a single item; and (c) being a parent of someone under 30. We decided to identify parents with children under 30 years of age because the MMR vaccine is mostly a childhood vaccine with available booster shots for later ages. This non-optimal age cut-off point for parental status was chosen because the study was designed as part of a larger time-sharing study of various other vaccines. Methodological covariates included gender of the respondents (because random assignment did not produce equivalent conditions, female coded = 1, male coded = 0) and sample type (methodological check given we ran the tests on the aggregated sample, see Data section for further details, main sample coded = 0, supplemental sample coded = 1). Supporting Information B in S1 File contains more details on question wording.

### Analytical strategy

Although we preregistered pairwise contrasts across our six conditions, in response to the feedback during revisions, we grouped the six conditions into two factors for analytical efficiency. The first factor, which we named hesitancy-inducing message factor, had two levels, which are hesitancy-inducing narrative and control. The second factor, which we named science message factor, had three levels: control, science-supporting message, and science-consistent narrative. Because of the large *N*, we used conservative Bonferroni adjustments for 15 possible pairwise comparisons.

To assess experimental effects, we utilized both general linear model (GLM) regression and analysis of covariance (ANCOVA) to predict the MMR vaccine risk perceptions and pro-vaccine policy views, because these outcome measures were scales (continuous variables composed of multiple items). We utilized logit models for encouraging others to vaccinate their children and agreeing to send a pro-vaccine letter to state representative because these measures were single-item ordinal/categorical measures. The models included the two factors and introduced the two main effects along with the combined effect (interaction) of the two factors, and other covariates. The results show mean differences for MMR risk perceptions (ranging from 1 to 4.25) and pro-vaccine policy views (ranging from 1 to 5) and shift in the predicted probabilities (0 to 1, see the two coding strategies below) for encouraging others and sending a pro-vaccine letter (0 or 1). Balanced (equally weighted) estimated marginal means are calculated as homogeneity of variances assumption was met. Cell-weighted ANCOVA models produced the same results. We included the same set of covariates for all models (see section above) with the exception of ideology, which was included in only two policy-related models (pro-vaccine policy support and sending a pro-vaccine letter to state representative), because it is a policy-relevant variable. Various statistical assumptions were checked, and are reported in Supporting Information B in S1 File. The assumption of normality was violated, although this is less problematic for comparisons involving random assignments (experiment), especially when the sample size is large and cell sizes are similar.

#### Recoding of encouraging others to vaccinate their children

Given the violations of some logit regression assumptions and the strong negative skew in intentions to encourage others to vaccinate their children (Supporting Information A in S1 File for further details), we recoded this outcome measure into a binary option consisting of lower likelihood (= 2 or 1, coded 0) and higher likelihood (= 3 or 4, coded 1) and conducted a binary logistic regression. Given that about half of respondents selected the highest likelihood (originally coded = 4), we also recoded it into a second/alternative binary option consisting of lower likelihood (= 3 or lower, coded 0) and highest likelihood (= 4, coded 1). We present these results for both coding strategies.

Finally, preregistration details that also include itemized deviations from the preregistration are in Supporting Information D in S1 File, additional analyses that relied on non-factorial analytical approach (preregistered) are in Supporting Information E in S1 File (of which results did not differ substantially), and finally, additional robustness checks are in Supporting Information F in S1 File.

## Results

**Fig 1:**
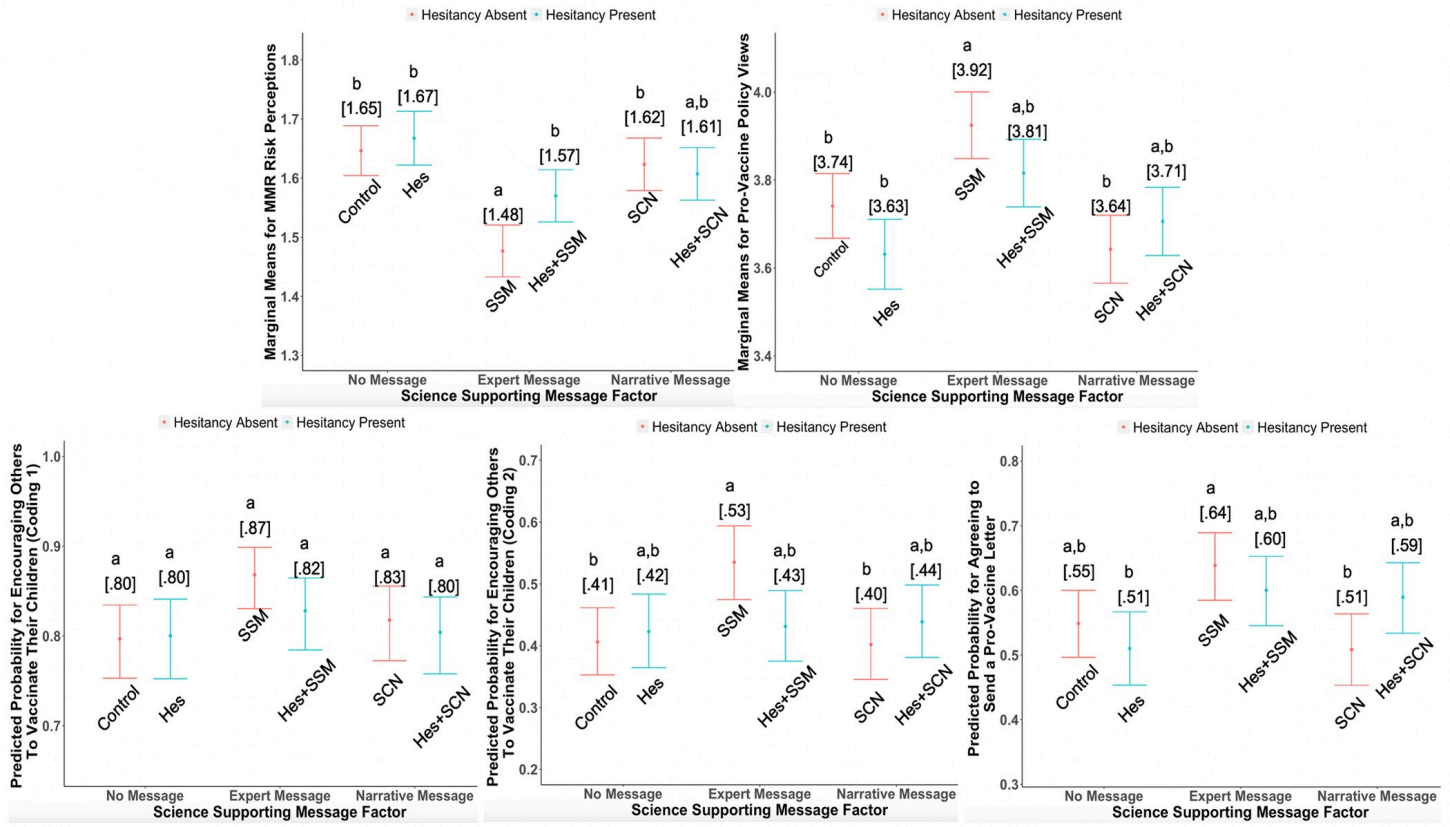
Hes–Hesitancy-inducing narrative, SSM–Science-Supporting Message, SCN–Science-Consistent Message. Shared letters denote that no significant differences exist among the conditions (which was based on Bonferroni correction for a total of 15 pairwise comparisons among the six conditions). Non-overlapping CIs do not imply significant difference due to the Bonferroni correction.

Table 1 contains coefficients for the main and interaction effects as well as coefficients for the covariates. The table is organized with vertical panels for each of our dependent measures. Both GLM regression and ANCOVA results are provided for the continuous outcome variables (for comparison purposes with the logit models of the categorical outcomes). For logit models, odd ratios are provided too. Fig 1 shows the estimated marginal means for the continuous dependent variables and the predicted probabilities for the categorical dependent variables. Bonferroni-corrected post-hoc contrasts were performed among the means and probabilities in Fig 1 and different letters indicate means or probabilities that are significantly different from each other. Full statistical details about the contrasts appear in Supporting Information B in S1 File.

We first examined the effects of our experimental messages on MMR vaccine risk perceptions, policy views, intentions to encourage others to vaccinate their children against measles (for both coding strategies), and intentions to send a pro-vaccine letter to a state representative. As shown in the second row of Table 1, exposure to the hesitancy-inducing narrative had no significant effect on our outcomes, except for a small negative effect on vaccine policy views, F(1,2280) = 3.96, p = .046. This generally null effect can also be seen in the five plots in Fig 1, which show no differences between the hesitancy-inducing narrative condition and the control condition after Bonferroni corrections were performed.

In contrast to the null main effects of the hesitancy-inducing narrative, the science-supporting message had significant positive effects for all our dependent measures (rows 3, 4, and 5 in Table 1). Specifically, presenting (vs. not presenting) the science-supporting message led to lower MMR vaccine risk perceptions, stronger pro-vaccine policy views, and stronger intentions to encourage others to vaccinate their children and send a letter to a representative. Of note, however, this main effect was mostly driven by the expert science-supporting message, which remained significantly different from both the control condition and hesitancy-inducing narrative in the contrasts in Fig 1. In contrast, the narrative science-supporting message did not differ from the control message.

We also examined the interaction between the hesitancy-inducing narrative and the science-supporting factor and conducted specific contrasts. We found that the hesitancy-inducing narrative and the science-supporting factor interacted significantly for vaccine risk perceptions, vaccine policy views, and encouraging others to vaccinate their children (the second coding strategy; see Table 1). These interactions can be decomposed by inspecting the contrasts in Fig 1 and also the planned interactions between each science-supporting message and the hesitancy-inducing narrative. As shown by the contrasts in Fig 1, when no hesitancy-inducing narrative was presented, the expert science-supporting message alone led to a weaker perception that the vaccine is risky than did either not presenting any science-supporting message (i.e., the control condition) or presenting the science-consistent narrative. However, when a hesitancy-inducing narrative was presented, vaccine risk perceptions did not differ across the science-supporting message, the science-consistent narrative, and the control conditions. As seen in Fig 1, this same pattern was present for the other outcome variables, although applying the conservative Bonferroni correction for 15 possible pairwise comparisons rendered those comparisons not significant. With respect to the more focused interactions, the combined effect of the hesitancy-inducing narrative x science-supporting expert message was significant only for the second coding strategy of encouraging others to vaccinate their children (Table 1). Overall then, because this interactive pattern was weak and not always significant, we recommend replicating this finding. Finally, the more focused interaction between the hesitancy-inducing message and the science-consistent narrative was significant for policy views and sending a letter, although the contrasts with Bonferroni corrections were not significant. We also note that all effect sizes were small as shown by the partial eta-squares and odds ratios in Table 1.

Our results lead us to the following conclusions about our hypotheses and research questions. First, we had hypothesized that the hesitancy-inducing narrative could reduce pro-vaccine views and intentions. Our null findings do not lend support to H1. The second hypothesis posited that presentation of science-supporting message factor would increase pro-vaccine views and intentions. Our findings were consistent with this hypothesis but only for the science-supporting message in which expert information was relayed. H2 is, as a result, partially supported. Third, we had hypothesized that the science-supporting message might counteract the potentially negative influence of hesitancy-inducing narrative when presented together. Presenting the science-supporting messages along with the hesitancy-inducing narrative did not result in stronger pro-vaccine views or intentions relative to the sole presentation of the hesitancy-inducing narrative; thus, H3 is not supported. Finally, for RQ1, the obtained differences between the science-supporting messages with and without the hesitancy-inducing narrative were mostly inconclusive. We found suggestive evidence (i.e., one significant finding. and several trends) that the hesitancy-inducing narrative could limit the pro-vaccine effect of the expert science-supporting message.

## Discussion

Understanding the effects of news coverage of measles and the MMR vaccine is crucial, especially in light of recent public health crises such as the U.S. measles outbreak as well as the potential of MMR misinformation to elicit hesitancy toward other known and novel vaccines [35]. This study offers a first empirical test of the potential influence of a personalized story of parents’ response to a child who suffered a vaccine side effect. Our study detected no significant effect of this message when presented alone: Except for one outcome variable (i.e., policy views), the vaccine-hesitant narrative by itself did not induce hesitancy or reduce pro-vaccine positions in a statistically significant way. This result is generally consistent with research suggesting that exposure to single messages rarely produces an impact [36] and stands in contrast to a study that reported increased vaccine risk perceptions due to exposure to a vaccine-critical website. However, the prior study is very different from ours in terms of topic, context, and methods [12]. Still, we should exercise caution when interpreting this non-significant finding as conclusive evidence given our mainly null effects. More research should be conducted in the future to test our hypothesis.

Our results also showed that when presented alone, the statistical information provided by an expert (i.e., the science-supporting message) about vaccine safety and effectiveness produced stronger pro-vaccine beliefs than did the control message, although the effect sizes were relatively small [31, 37]. However, for vaccine risk perceptions, the science-supporting expert message mostly ceased to offer a benefit when it followed the hesitancy-inducing narrative. Additionally, although when combined with the hesitancy-inducing narrative the expert science-supporting message did not differ from the other four conditions for any of our outcome measures, it differed from the other conditions significantly when it was presented alone. We interpret this particular finding and the trend in other outcome measures as potential evidence that the hesitancy-inducing narrative may weaken the pro-vaccine influence of the expert statistical message [37]. Future work should further examine this process to determine if it occurs in other contexts. We note that effects might differ, and we suspect might be more pronounced, if respondents were exposed to more tightly circumscribed, experimenter-created messages rather than the real-world ones studied here. By contrast, our data provide the ecological validity of using real news clips in the context of an actual outbreak.

We also found that the pro-vaccine narrative message did not have a significant effect either alone or in combination with the hesitancy narrative, showing its relative ineffectiveness compared to the statistical message about the MMR vaccine [19]. This finding also stands in contrast to the literature showing the effectiveness of narrative corrections [5]. More research is needed to understand how to increase the effectiveness of the appeals we tested in this experiment.

Our findings offer empirical evidence on a variety of vaccine-related beliefs and intentions. Investigating these diverse outcomes is important in real-world contexts and during disease outbreaks. For example, experimental research on policy support and behaviors is scarce [34, 38]. In light of ongoing legislative discussion and action concerning medical, religious, and personal belief exemptions at the level of the states [39, 40], understanding the impact of news stories on support for pro-vaccine and anti-vaccine policies is important. We showed that pro-vaccine scientific messages boost both pro-vaccine policy views and the willingness to send a pro-vaccine letter, although the small effect sizes should be kept in mind. Still, the empirical evidence of effects during an outbreak is important and should be replicated for other outbreaks and pandemics such as COVID-19. Although our effect sizes were small, such effects could have significance at the societal level during pandemics.

In addition to theoretical contributions to our understanding of persuasion effects, this study adds to our understanding of vaccine-related messages in ways that are important methodologically. Most experimental research on vaccine attitudes and misinformation has relied on small convenience samples [12, 14, 23, 41], online convenience samples such as Amazon Mechanical Turk [42, 43], other online *non-probability* panels [37], or in few cases, probability samples of specialized populations (i.e., only parents, which would be not be an ideal sample when we focus on outcomes such as policy views) [19]. Work on narrative bias and vaccine side effects that relies on fictitious diseases and hypothetical vaccines makes it difficult to render a judgment about real vaccines [13–15]. Prior studies also focused mostly on print text, and occasionally still photographs [19], but not on actual broadcast news segments. Importantly, industry statistics show that most individuals prefer to get their news on television (e.g., United States) [44].

## Limitations

Due to heightened media attention during the measles outbreak, participants may have been exposed to multiple news stories and a great deal of social media content about measles and the MMR vaccine, which may have affected their responsiveness to our experimental materials. Because of randomization and media exposure as a covariate, we would expect these effects to be comparable across conditions and not to contaminate our results.

Second, our findings are limited to vaccination against MMR, and may therefore operate differently for other vaccines, such as those against the flu, HPV (human papillomavirus) or COVID-19. Different vaccines also elicit different considerations. For example, the HPV vaccine elicits religious and parental concerns because of the assumption of some that it signals acceptability of sexual activity for teenagers. These differences could lead to different patterns of message processing concerning descriptions of side effects. Relatedly, future research should examine how other theoretical variables such as religiosity might alter reactions to such experimental messages.

Third, although our manipulations sought to maximize external validity by using edited versions of real news videos that were aired in the middle of a national measles outbreak, future research should replicate these effects in different media (e.g., news articles) and come up with designs to disentangle some of the effects we observed. One potential problem with less-controlled real news clips is that we cannot know, for example, whether beliefs shift because of the expert authority acting as a source cue, the nature of statistical information provided, or both. This aspect may also contribute to the study’s small effect sizes, which we note as another limitation. We also are unsure of whether participants processed the source and message information separately [45]. Similarly, we do not currently know whether the effect of the hesitancy-inducing narrative was produced by the credibility of the mother or the evocative visual image of the rash-ridden child [46,47]. Finally, our video content may not be fully representative of the media environment, which is both diverse and fast changing. These issues should be addressed in future research.

### Implications for public health campaigns: A transparency dilemma?

Our results have important implications for public health campaigns and decisions in newsrooms as journalists tackle vaccine hesitancy-inducing or -reinforcing narratives. First, although the statistical message had more favorable and more consistently positive effects, public health campaigns and news coverage that aim to promote vaccination may benefit from using both the scientific messages and pro-vaccine narratives that amplify the stories of individuals and that may better address the emotional aspect of the misinformation [5]. Since we do not find negative effects of pro-vaccine parental stories, more research should be conducted to judge their effectiveness in different formats and contexts. Second, giving voice to vaccine-hesitant parents as a way of explaining the increase in measles cases can still have detrimental effects [21, 48, 49]. Moreover, the effect of a hesitancy-inducing narrative may increase if repeated exposure occurs or if the stories circulate within social media where repetition and like–minded interpersonal interaction prevail [17, 50]. More research is needed to determine how to communicate the relatively low risk involved in vaccination compared to the higher risks of vaccine-preventable infectious diseases.

At a broader level, these findings also speak to the importance of having the scientific community and news media acknowledge and carefully communicate the rare but real side effects of vaccines. As science writer Melinda Wenner Boyer noted, “*Scientists are so terrified of the public’s vaccine hesitancy that they are censoring themselves*, *playing down undesirable findings and perhaps even avoiding undertaking studies that could show unwanted effects”* [51]. This anecdotal evidence by a leading science writer at *The New York Times* should be taken as an early warning about the complexity and challenges in the public discourse on vaccine safety. Failure to be transparent about the side effects of vaccines and to communicate them effectively may enhance the credibility of anti-vaccination rhetoric. The reluctance of scientists to talk about side effects leaves the media with little guidance on how to cover them.

## Supporting information

S1 File (DOCX)
