# Supplementary material for: The effects of scientific messages and narratives about vaccination
Source: PLoS One. 2021 Mar 24;16(3):e0248328. doi: 10.1371/journal.pone.0248328 (PMC7990169; doi:10.1371/journal.pone.0248328)
Supplement: S1 File — (DOCX) [file pone.0248328.s001.docx]

**S1 File**

**Supporting Information A.** Salience of Measles and MMR in the Traditional and Social Media

**Supporting Information B.** Other Methodological and Analytical Details

**Supporting Information C.** Item by Item Analysis Main Results for Index Scores

**Supporting Information D.** Preregistration and Itemized Deviations from the Pre-registration

**Supporting Information E.** Old Results from (Non-factorial) Regression Analysis and Their Supplementary Analyses

***Subsection 1:*** Details for Main Effects

***Subsection 2:*** Details for Vaccine Misinformation Moderation

***Subsection 3:*** Odds Ratios for Binary Logit Model

***Subsection 4:*** Results Including Respondent Ideology for the Two Policy Related Outcome Variables

***Subsection 5:*** Methodological Check – Device of Survey Respondent (PC vs Smartphone/Tablet) and Sample Type

***Subsection 6.*** Other Results for Preregistered Tests (Other outcome measures, moderator, and the longitudinal effects)

**Supporting Information F.** Additional Preregistered Analyses and Robustness Checks

***Subsection 1:*** Other Outcome Measures Pre-registered

***Subsection 2:*** Vaccine Misinformation Moderation

***Subsection 3:*** Device of Survey-taking (PC vs phone/tablet)

**Supporting Information A. Salience of measles and MMR in the Traditional and Social media**

According to data from Factiva, the Associated Press and Reuters newswire agencies have been increasingly more likely to cover measles. Volume of news coverage mentioning the word “measles” increased from 117 stories in 2017, to 225 stories in 2018, and to 456 stories between January and April of 2019. According to Google search trend analyses, public attention has been increasing as well. The period during which Google has been tracking these data (2014 to present) has shown two peaks in search interest in measles. The first one was in February of 2015 (Disneyland outbreak) and the second one was May of 2019. The 2019 peak is also projected to surpass the 2015 peak and will likely comprise the highest volume ever of measles related Google searches. We therefore conclude that measles is as salient of an issue as it has ever been in recent US history.

**Supporting Information B. Methodological Details**

**The Sample**

The (weighted and unweighted) demographic composition of Wave 1 original data, the experiment sample 1 (Wave 4, N = 1,803), the second experiment sample (control sample, N = 1,006), and the latest census estimates are provided below in Table B1 for comparison purposes. The original Wave 1 data matched latest U.S. Census Bureau estimates regarding key demographics (i.e. age, gender, race, income) that were available, except for education. There were more respondents with high education compared to the census. Hence, NORC implemented *adaptive targeting* (e.g., more incentives, phone calls/reminders) of the respondents with low education to keep education distribution from getting more skewed. More details on NORC sampling procedures, please refer to “https://tessexperiments.org/NORC AmeriSpeak Information for IRBs 2016 10 18.pdf” by Dennis JM. *Documentation for NORC’s AmeriSpeak for Institutional Review Boards*.

**Table B1.** Comparison of Sample Demographics (% without and with Weights) with the Most Recent U.S. Census Bureau Estimates for Key Demographic Variables – Age, Sex, Race, Education, Income

| **Category** | **Panel**  **Wave 1**  **N = 3,005** | **Panel**  **Wave 1**  **N = 3,005 Weighted** | **Panel**  **Wave 4**  **N = 1,803** | **Panel**  **Wave 4**  **N = 1,803**  **Weighted** | **Control**  **N = 1,006** | **Control**  **N = 1,006**  **Weighted** | **U.S. Census Est.** |
| --- | --- | --- | --- | --- | --- | --- | --- |
| **Age** |  |  |  |  |  |  |  |
| 18-24 | 10.3 | 11.7 | 8.8 | 11.7 | 7 | 11.7 | 12.6 |
| 25-44 | 35.4 | 34.9 | 35.3 | 35.1 | 38.3 | 34.5 | 34.2 |
| 45-64 | 30.7 | 32.9 | 32.3 | 32.8 | 33.1 | 33.3 | 33.5 |
| 65 and above | 24.6 | 20.4 | 23.6 | 20.4 | 21.6 | 20.4 | 20.2 |
|  |  |  |  |  |  |  |  |
| **Sex** |  |  |  |  |  |  |  |
| Females | 52.9 | 51.7 | 51.6 | 51.7 | 46.6 | 51.7 | 51.3 |
| Males | 47.1 | 48.3 | 48.4 | 48.3 | 53.4 | 48.3 | 48.7 |
|  |  |  |  |  |  |  |  |
| **Race - Non-Hispanic Identifying** |  |  |  |  |  |  |  |
| Non-Hispanic White | 60.3 | 63.4 | 63.6 | 63.4 | 63.4 | 63.4 | 60.7 |
| Non-Hispanic Black or African American | 13.8 | 11.9 | 12.4 | 11.9 | 11 | 11.9 | 12.5 |
| Non-Hispanic Asian | 6 | 4.2 | 5.9 | 4.2 | 4.4 | 2.8 | 5.7 |
| Non-Hispanic American Indian and Alaska Native | - | - | - | - | - | - | 0.7 |
| Non-Hispanic Native Hawaiian and Pacific Islander | - | - | - | - | - | - | 0.2 |
| Non-Hispanic Two or more races | 3.6 | 3 | 2.7 | 2.2 | 4.2 | 4.8 | 2.1 |
| Non-Hispanic Other (includes Indian, Pacific) | 1.5 | 1.3 | 1.5 | 1.4 | 0.9 | 0.8 |  |
| **Race - Hispanic Identifying** | - | - | - | - | - | - |  |
| Just “Hispanic” | 14.9 | 16.3 | 14 | 16.3 | 16.1 | 16.3 |  |
| Hispanic White | - | - | - | - | - | - | 15.9 |
| Hispanic Black or African American | - | - | - | - | - | - | 0.9 |
| Hispanic Asian | - | - | - | - | - | - | 0.2 |
| Hispanic American Indian and Alaska Native | - | - | - | - | - | - | 0.5 |
| Hispanic Native Hawaiian and Pacific Islander | - | - | - | - | - | - | 0.1 |
| Hispanic Two or more races | - | - | - | - | - | - | 0.6 |
|  |  |  |  |  |  |  |  |
| **Education** |  |  |  |  |  |  |  |
| None | 0.1 | 0.4 | 0.1 | 0.2 | 0.1 | 0.3 | 0.3 |
| 1st - 4th grade | 0 | 0 | 0 | 0 | 0.1 | 0 | 0.6 |
| 5th - 6th grade | 0.2 | 0.3 | 0.1 | 0.2 | 0.5 | 0.5 | 1.3 |
| 7th - 8th grade | 0.5 | 1.3 | 0.4 | 0.9 | 0.5 | 0.7 | 1.5 |
| 9th grade | 0.5 | 1.2 | 0.5 | 1.3 | 0.3 | 0.3 | 1.4 |
| 10th grade | 0.6 | 1.5 | 0.4 | 1.1 | 0.7 | 1.2 | 1.6 |
| 11th grade | 0.9 | 2.1 | 0.8 | 2.4 | 1.7 | 3.3 | 4.2 |
| 12^th^ grade no diploma | 1.6 | 3.8 | 1.7 | 4.6 | 1.8 | 4.3 | - |
| High school graduate | 14.5 | 28.6 | 15.1 | 28.6 | 14.4 | 28.6 | 28.6 |
| Some college, no degree | 23.5 | 19.3 | 21.9 | 19.6 | 24 | 20.5 | 18.5 |
| Associate's degree | 10.7 | 9 | 9.7 | 8.7 | 10.5 | 7.8 | 9.7 |
| Bachelor's degree | 28.2 | 19.3 | 28.5 | 18.6 | 26.9 | 10.2 | 20.6 |
| Master's degree | 13.2 | 9.3 | 15 | 10.1 | 13.7 | 9.4 | 8.5 |
| Professional or doctorate degree | 5.5 | 3.8 | 5.8 | 3.8 | 4.8 | 3.9 | 3.1 |
|  |  |  |  |  |  |  |  |
| **Income** |  |  |  |  |  |  |  |
| Under $15,000 | 8.9 | 10.9 | 8 | 10.5 | 9.6 | 11.5 | 10.7 |
| $15,000 to $24,999 | 10.4 | 11.1 | 10.1 | 10.7 | 9.6 | 11.8 | 9.6 |
| $25,000 to $34,999 | 11.5 | 11.7 | 11.1 | 12 | 10.3 | 12.5 | 9.2 |
| $35,000 to $49,999 | 12.4 | 12.8 | 12 | 12.6 | 11.9 | 12.7 | 12.3 |
| $50,000 to $74,999 | 18.2 | 17.9 | 18.7 | 17.8 | 18.8 | 17.8 | 16.5 |
| $75,000 to $99,999 | 14.9 | 13.9 | 15.3 | 13.6 | 16 | 14.4 | 12.5 |
| $100,000 to $149,999 | 15 | 13.3 | 15.4 | 13.1 | 15.2 | 11.5 | 14.5 |
| $150,000 to $199,999 | 4.8 | 4.5 | 5 | 4.5 | 4.7 | 4.2 | 7 |
| $200,000 and over | 4.1 | 4 | 43 | 3.9 | 4.2 | 3.8 | 7.70 |
| number of households | - | - | - | - | - | - | 127,586 |
| median income | $50-59k | $50-59k | $50-59k | $50-59k | $60-75k | $60-75k | $61,372 |
| average income | $50-59k | $50-59k | $50-59k | $50-59k | $50-59k | $50-59k | $86,220 |

**Notes.** Values are %s unless otherwise stated. For age, sex, race, and income, the most recent estimates provided by the Census Bureau are for 2017, for education, the most recent estimate is for 2018. Estimates are projections based on the 2010 Census. For income, %s are given for households. For race, %s are given separately for those who identify as non-Hispanic or Hispanic. For age and education, %s are given for those above 18 years old. For gender, %s are given for all individuals (including below 18 years old). Income refers to total household income categories, percentages reflect the proportion of income within the total number of households in the US in 2017 (N=127,586). Average and median income for the samples represent the response category, not the exact amount like in the census estimates. Census resources” For age and sex, see <https://factfinder.census.gov/faces/tableservices/jsf/pages/productview.xhtml?src=bkmk>; for race, see <https://factfinder.census.gov/faces/tableservices/jsf/pages/productview.xhtml?src=bkmk>; for education, see <https://www.census.gov/data/tables/2018/demo/education-attainment/cps-detailed-tables.html>; for income, see <https://www.census.gov/data/tables/2018/demo/education-attainment/cps-detailed-tables.html>.

**Power analysis and sample combination:**

Given our prior attrition rates (20%), and the prior (wave 3) sample size N = 2,100, we expected we would obtain about 1700 respondents for the first (panel) sample, and this would correspond to approximately 280 respondents per condition. To supplement this sample and to test for panel sensitization, we decided to obtain a second sample. We ran a power analysis for a d = .10 (based on Cohen recommendations for small effects, which we considered characteristic of media effects, especially given that our messages were real news video clips), and found that approximately a sample size of N = 410 was required for an alpha level of p= .001 (due to multiple tests) and a power of .80. As we found out that it is underpowered, we decided to combine two samples. As a result, we wanted to have at least 130 more respondents per condition in this combined sample to reach N = 410 per condition. This determined our sample size in the second sample in which we ended up having approximately 140 respondents for each of the six conditions. Since we ran our analyses on the combined sample, we included a dummy variable indicating sample type as a control variable. We conducted all tests on each sample alone as well, of which results are provided in the relevant section below in the Supporting Information.

**Manipulation Videos**

C1, Hes: Hesitancy-inducing narrative

<https://upenn.app.box.com/s/mtu4q5iftkctd8jqnhkxn0my9fn0pca3/file/397025682383>

C2, SSM: Science-supporting message

<https://upenn.app.box.com/s/mtu4q5iftkctd8jqnhkxn0my9fn0pca3/file/397037158867>

C3, SCN: Science-consistent narrative

<https://upenn.app.box.com/s/mtu4q5iftkctd8jqnhkxn0my9fn0pca3/file/397043335324>

C4, Hes+SSM: Hesitancy + Science-message

<https://upenn.app.box.com/s/mtu4q5iftkctd8jqnhkxn0my9fn0pca3/file/397051486037>

C5, Hes+SCN: Hesitancy + Science-narrative

<https://upenn.app.box.com/s/mtu4q5iftkctd8jqnhkxn0my9fn0pca3/file/397051855463>

C6, Control

<https://upenn.app.box.com/s/mtu4q5iftkctd8jqnhkxn0my9fn0pca3/file/397050735496>

**Original Video Sources – Editing Details**

Measles Sources:

NBC Nightly News with Lester Holt – 1/23/3019 (6:30pm EST)

CBS Evening News with Jeff Glor – 1/23/2019 (6:30pm EST)

ABC World News Tonight with David Muir – 1/23/2019 (6:30pm EST)

Anthony Fauci segment from PBS Newshour 1/28/2019 (6pm EST) – SSM condition

Videos can be found by searching TV News Archive (<https://archive.org/details/tv>)

Hes condition is all from CBS News.

SCN condition uses clips from NBC, ABC, CBS, and NBC News.

CTRL uses the content of “An Aspirin a Day isn’t Always Recommended” (Associated Press YouTube channel) <https://www.youtube.com/watch?v=rRI-ZaC71Js>

**Manipulation Checks**

At the beginning of the study, respondents were told that they would need audio equipment; they were encouraged to test the speakers (or headphones) of their device. After this, respondents listened to an audio-coded message containing a few numbers which they had to type in order to start the survey. This procedure ensured that respondents were able to hear our news clips.

We evaluated whether the manipulations worked by presenting eight items to all respondents. Four of the items were false, such that they did not appear in any condition/video. The rest of the items appeared either alone (in CTRL, Hes, SSM, or SCN conditions) or in combination conditions. Respondents were able to select all items that they think were included as content in the videos (yes or no as response options). This way we were able to assess whether manipulations worked in a standardized way for all respondents across all conditions. In the table below, absolute %s are given, and note that 1 to 7% (across the different conditions) of respondents who took the survey on the Internet skipped/did not answer the manipulation check. The results show that our manipulations worked. The only unexpected pattern we found concerns the SCN condition, where 53% of the respondents in that condition selected an item that did not appear in the video; although 86% correctly selected the item that they were supposed to select. When we examined the video in detail to understand why this might have happened (53% incorrectly selecting the item “a mother’s concerns with the side effects of the measles, mumps, and rubella (MMR) vaccine on children”), we thought one reason for this could be that some respondents did not read this statement fully and carefully as it was the longest statement among the eight (this item had similar but smaller % in the SSM condition as well).

**Table C1.** Manipulation Checks

| **Statement** | **% stating this was included in the video** | | | | | |
| --- | --- | --- | --- | --- | --- | --- |
| The video I saw included content about… | Hes | SCN | SSM | Hes+SCN | Hes+ SSM | Control |
| aspirin and heart disease | 2 | 2 | 2 | 2 | 2 | 97 |
| market research on the popularity of using herbal remedies in fighting infectious diseases | 2 | 5 | 5 | 4 | 3 | 3 |
| accessibility of the flu vaccine in schools in rural America | 5 | 12 | 10 | 7 | 7 | 4 |
| parental concerns about growing childhood obesity | 2 | 3 | 4 | 4 | 2 | 2 |
| a mother discussing a C section resulting from measles | 2 | 86 | 3 | 85 | 4 | 2 |
| parental concerns about the side effects of vaccination on the elderly | 7 | 7 | 7 | 9 | 10 | 4 |
| the risk of measles discussed by a medical expert | 11 | 44 | 95 | 44 | 91 | 6 |
| a mother’s concerns with the side effects of the measles, mumps, and rubella (MMR) vaccine on children | 97 | 53 | 14 | 96 | 97 | 5 |

**General Assessments of the video**

Participants were asked whether the video content was clear, an overwhelming majority reported that they strongly agreed (72%) or somewhat agreed (23%) that the video was clear, with only 5% disagreeing. Respondents substantively varied in their agreement with whether video was engaging, was complex, or presented new information that they did not know before. The distributions across the conditions were similar in reported engagement and complexity while the science-supportive expert video was judged as presenting more novel information than the others (Agreement for SSM = 75% vs Agreement Range for other conditions = 38% to 66%).

**Measures**

**Table.** Full Question Wordings

| **Name** | **Question** | **Response options** |
| --- | --- | --- |
|  |  |  |
| MMR Vaccine Risk Perceptions (α = .81) | | |
| Item 1 (Risk) | Just your best guess, how risky, if at all, do you think the measles, mumps, and rubella (MMR) vaccine is? | Not risky at all (original code = 1), Not too risky (2), Somewhat risky (3), Very risky (4). |
| Item 2 ( Effectiveness) | Just your best guess, please indicate how effective, if at all, you think the measles, mumps, and rubella (MMR) vaccine will be at preventing measles among those who get the vaccine in the future? (reversed) | Not effective at all (original code = 1), Not too effective (2), Somewhat effective (3), Very effective (4) |
| Item 3 (Relative Risk) | Based on what you know, is the measles, mumps, and rubella (MMR) vaccine: | Much less risky than catching measles (original code = 1), Slightly less risky than catching measles (2), As risky as catching measles (3), Slightly more risky than catching measles (4), Much more risky than catching measles (5). |
| Item 4 (Negative Affect) | Based on what you know, how positive or negative do you feel about the measles, mumps, and rubella (MMR) vaccine? (reversed) | Very negative (original code = 1), Somewhat negative (2), Somewhat positive (3), Very positive (4) |
|  |  |  |
| Pro-Vaccine Policy Views (α = .85) | | |
|  | Consider the following statements referring to different kinds of laws that states may put into place about vaccines. For each one, please indicate whether you would strongly support, somewhat support, neither support nor oppose, somewhat oppose, or strongly oppose such a law. | |
| Item 1 | It should be mandatory for parents to vaccinate their children against preventable diseases such as measles, mumps, and rubella. | Strongly support (original code = 1), Somewhat support (2), Neither support nor oppose (3), Somewhat oppose (4), Strongly oppose (5) |
| Item 2 | The state I live in should allow parents to choose not to vaccinate their children for medical reasons. | Strongly support (original code = 1), Somewhat support (2), Neither support nor oppose (3), Somewhat oppose (4), Strongly oppose (5) |
| Item 3 | The state I live in should allow parents to choose not to vaccinate their children for medical reasons. (reversed). | Strongly support (original code = 1), Somewhat support (2), Neither support nor oppose (3), Somewhat oppose (4), Strongly oppose (5) |
| Item 4 | The state I live in should allow parents to choose not to vaccinate their children for religious reasons. (reversed). | Strongly support (original code = 1), Somewhat support (2), Neither support nor oppose (3), Somewhat oppose (4), Strongly oppose (5) |
| Item 5 | The state I live in should allow parents to choose not to vaccinate their children for personal or philosophical reasons. (reversed). | Strongly support (original code = 1), Somewhat support (2), Neither support nor oppose (3), Somewhat oppose (4), Strongly oppose (5) |
| Item 6 | The state I live in should set aside public funds for free or discounted access to the measles, mumps, and rubella (MMR) vaccines for all children. | Strongly support (original code = 1), Somewhat support (2), Neither support nor oppose (3), Somewhat oppose (4), Strongly oppose (5) |
|  |  |  |
| Intention to Encourage Other Parents to Vaccinate Their Children | | |
| Item 1 | How likely are you to encourage parents to give the measles, mumps, and rubella (MMR) vaccine to children? | Not likely at all (original code = 1), Not too likely (2), Somewhat likely (3), Very likely (4) |
|  |  |  |
| Agreeing to Send Letter to State Representative | | |
| Item 1 | We have prepared the following statement in support of a law that would require childhood vaccinations in your state. It reads: “I strongly support a bill that would improve childhood immunization rates in my [current state of residence] and protect children and our communities from disease and death resulting from vaccine-preventable illness. The bill should remove the ‘personal belief’ exemptions from the school immunization law and also require the governing board of a school district to notify parents or guardians of a school’s immunization rates.” Would you like us to contact your State Representative in your name with that message?” | Yes (coded 1), No (coded 0) |
|  |  |  |
| Vaccine Misinformation (α = .80)  For each of the statements listed below, please indicate how accurate you think they are: | | |
| Item 1 | Vaccines given to children for diseases like measles, mumps, and rubella can cause neurological disorders like autism. | Very Inaccurate original code = 1), Somewhat Inaccurate (2), Somewhat Accurate (3), Very Accurate (4) |
| Item 2 | Vaccines in general are full of toxins and harmful ingredients like “antifreeze.” | Very Inaccurate original code = 1), Somewhat Inaccurate (2), Somewhat Accurate (3), Very Accurate (4) |
| Item 3 | It makes no difference if parents choose to delay or spread out vaccines instead of relying on the official CDC vaccine schedule. | Very Inaccurate original code = 1), Somewhat Inaccurate (2), Somewhat Accurate (3), Very Accurate (4) |
| Item 4 | It is better to develop natural immunity by getting the disease than by receiving the vaccine. | Very Inaccurate original code = 1), Somewhat Inaccurate (2), Somewhat Accurate (3), Very Accurate (4) |
|  |  |  |
|  | Trust in Medical Authorities (α = .80)  How much trust, if at all, do you have in the following to give you accurate information about the benefits and risks of vaccination? | |
| Item 1 | US Centers for Disease Control and Prevention (CDC) | A great deal of trust (original code = 1), Some trust (2), Not too much trust (3), Very little trust at all (4) |
| Item 2 | Your primary care doctor or primary medical provider | A great deal of trust (original code =1), Some trust (2), Not too much trust (3), Very little trust at all (4) |
|  |  |  |
|  | Conservative-Liberal Ideology | |
| Item 1 | **Generally speaking, would you describe your political views as:** | Very conservative (original code = 1), Somewhat conservative (2), Moderate (3), Somewhat liberal (4), Very liberal (5) |
|  |  |  |
|  | Exposure to Measles Related Media | |
| Item 1 | Since [last time the respondent took the survey], how frequently have you read, heard or seen news about measles in newspapers, magazines, on radio, or on television? | Never (original code = 1), Rarely (2), Sometimes (3), Often (4) |
|  |  |  |
|  | Parent | |
| Item 1 | Are you the parent or guardian of any children under the age of 30? | Yes (1), No (0) |

Vaccine misinformation. The original four-point scale ranges from 0-3, with higher numbers indicating more anti-vaccine beliefs. Median value for our sample is 0.5. 31% of the sample scored zero at vaccine misinformation, 12% of the sample is at the median value, and 1% of the sample is at the maximum value. Other outcome measures that were not included in the main report are: feelings of afraid and disgust about measles, perceived severity of complications that measles can cause, concern of contracting measles, and likelihood of sharing/posting the video that respondents have just watched.

Full question wording for these items are provided below:

**Perceptions of Measles (Outcome variable):**

“How afraid, if at all, do you feel about measles? (MEAS1) Very afraid, Somewhat afraid, Not too afraid, Not afraid at all

“How disgusted, if at all, do you feel about measles?” (MEAS2) Very disgusted, Somewhat disgusted, Not too disgusted, Not disgusted at all

“Just your best guess, how severe (e.g., life threatening, causing major illness), if at all, do you think complications from measles can be?” (MEAS4) Not severe at all, Not too severe, Somewhat severe, Very severe

“How concerned, if at all, are you that measles will become widespread throughout the United States?” (MEAS5) Very concerned, Somewhat concerned, A little concerned, Not at all concerned

The greater scores of this measles risk perceptions index represent greater risk associated with measles.

**Sharing the manipulation video (Outcome variable):**

How likely would you be to share or post the video you have just watched on your social media profile, such as Facebook, or via email? (POLVW2). Not likely at all, Not too likely, Somewhat likely, Very likely, Don’t use social media or email.

**Misperceptions about vaccines (Outcome variable):**

For each of the statements listed below, please indicate how accurate you think they are:(A) Severe side effects to the measles, mumps, and rubella (MMR) vaccine are common. (B) The CDC has not studied the risks of measles, mumps, and rubella (MMR) vaccine sufficiently. (C) There is no treatment for measles. (D) Some people, such as infants or people with specific health problems cannot receive measles, mumps, and rubella (MMR) vaccine. (E) For the measles, mumps, and rubella (MMR) vaccine to be effective, two doses are necessary. Response options were Very Inaccurate, Somewhat Inaccurate, Somewhat Accurate, Very Accurate. The greater scores in this index represent more misinformation.

**Potential Issue with policy question addressed**

Note that as of 2019, a great majority of states, 45 of them, provide non-medical (either religious, personal or philosophical) exemptions for mandatory school vaccinations; only California, Mississippi, West Virginia, New York and Maine do not provide such exemptions. Some of the exemptions are also vaccine specific, so our question does not perfectly match the legislation. We examined the results for this outcome variable by controlling for the respondents from these five states (N= 458, 20%) as well and did not find conflicting patterns. It is likely that most respondents, although we did not measure it, did not know about their state’s specific legislation status. Despite this complexity in the legislation details, the main point of the hypothetical proposal is broadly about supporting or opposing moves that would boost vaccination rates, hence we think this is a robust question tapping legislation support intentions.*http://www.ncsl.org/research/health/school-immunization-exemption-state-laws.aspx

**Assumption Tests for Analytical Models**

**ANCOVA assumptions.** First, both the MMR vaccine risk perceptions and pro-vaccine policy views had a linear relation with the covariates as assessed by visual inspection of scatter plots. The regression slopes were similar across the different levels of covariates as indicated by nonsignificant (*p* > .05) interactions between the covariates and our two experimental factors. Second, we checked for heteroskedasticity. The Shapiro Wilk test was significant (*p* < .05), as were the results of Breusch-Pagan test and the *F*-test for heteroskedasticity. However, residual and normal Q-Q plots showed a small deviation from normality. Transformations for the outcome variable (i.e., log, sqrt, cubic and Box-Cox) did not remove the detected heteroskedasticity fully. Hence, we also checked results with robust standard estimators and White adjustment to account for this violation, achieving substantively similar results. Third, we checked for the assumption of homogeneity of variance and outliers. The Levene’s test was not significant (p > .05), leading to the conclusion that this assumption was fully met. There were a small number of multivariate outliers (less than 30 and 20 for the MMR vaccine risk perceptions and pro-vaccine policy views, respectively), removal of which did not change the results.

**Logit regression assumptions.** We also checked assumptions for our measures of intentions to encourage others to vaccinate their children and to send a pro-vaccine letter to state representative, which were single items with ordinal and binary response options, respectively. These variables were analyzed using ordinal and binary logit models with the same covariates included in the ANCOVAs. We checked for three assumptions: (a) multicollinearity, (b) proportional odds assumption, and (c) heterogeneity of effects at different levels of the dependent measure. First, the variance inflation factor was low for the logit models (*VIF* < 2) and hence no multicollinearity was detected. Second, the proportional odds assumption did not hold for the ordinal logit model (predicting encouraging others to vaccinate their children), and this was driven by heterogeneous effects for the vaccine misinformation predictor. Given the model assumption violations and the strong negative skew in intentions to encourage others to vaccinate their children, we recoded this outcome measure into a binary option consisting of lower likelihood (= 2 or 1, coded 0) and higher likelihood (= 3 or 4, coded 1) and conducted a binary logistic regression. Given that about half of respondents selected the highest likelihood (originally coded = 4), we also recoded it into a second/alternative binary option consisting of lower likelihood (= 3 or lower, coded 0) and highest likelihood (= 4, coded 1). We present results for both coding strategies in the main paper. We also ran the ordinal logit by collapsing the two lowest level (and lowest cell N size) response options (Not likely at all and Not too likely) only and ending up with three levels. This model produced similar results. Finally, we ran the ordinal logit by removing the vaccine misinformation predictor to address the proportional odds assumption in that non-binary ordinal model, finding substantively same results.

**Additional Details for the Table 1 and Figure 1 reported in the Manuscript:**

**Table.** Means, Adjusted Means, Cell, Ns, and Percentages for the Four Outcome Variables across the Six Conditions

|  | **Experimental Conditions** | | | | | | |
| --- | --- | --- | --- | --- | --- | --- | --- |
| **Factor 1 – Hesitancy-Inducing Message:** | *Hesitancy-Inducing Message Absent* | | |  | *Hesitancy-Inducing Message Present* | | |
| **Factor 2 – Science-Supporting Message:** | No Message  (Control) | Expert Message  (SSM) | Narrative Message  (SCN) |  | No Message  (Hes) | Expert Message  (Hes+SSM) | Narrative Message  (Hes+SCN) |
|  |  |  |  |  |  |  |  |
| **MMR Vaccine Risk Perceptions (Means)** | | | | | | | |
| M | 1.66 | 1.50 | 1.61 |  | 1.65 | 1.57 | 1.60 |
| (sd) | (.65) | (.66) | (.67) |  | (.66) | (.60) | (.62) |
| M_adj_ | 1.65 | 1.48 | 1.62 |  | 1.67 | 1.57 | 1.61 |
| (se) | (.02) | (.02) | (.02) |  | (.03) | (.02) | (.02) |
| **Pro-Vaccine Policy Support (Means)** | | | | | | | |
| M | 3.71 | 3.88 | 3.67 |  | 3.69 | 3.81 | 3.70 |
| (sd) | (.96) | (.95) | (1.05) |  | (.98) | (.92) | (.98) |
| M_adj_ | 3.74 | 3.92 | 3.64 |  | 3.63 | 3.81 | 3.71 |
| (se) | (.04) | (.04) | (.04) |  | (.04) | (.04) | (.04) |
| **Encouraging Others to Vaccinate Their Children (Cells Ns and %S)** | | | | | |  |  |
| Not likely at all | 41  (9.69%) | 31  (7.93%) | 36  (9.38%) |  | 36  (9.86%) | 31  (8.01%) | 34  (8.90%) |
| Not too likely | 65  (15.37%) | 43  (11.00%) | 49  (12.76%) |  | 50  (13.70%) | 54  (13.95%) | 57  (14.92%) |
| Somewhat likely | 132  (31.21%) | 109  (27.88%) | 121  (31.51%) |  | 104  (28.49%) | 120  (31.01%) | 109  (28.53%) |
| Very likely | 185  (43.74%) | 208  (53.20%) | 178  (46.35%) |  | 175  (47.95%) | 182  (47.03%) | 182  (47.64%) |
| **Agree to Send Letter (Cell Ns and %s)** | |  |  |  |  |  |  |
| No | 195 (45.99%) | 154  (39.29%) | 185  (48.18% |  | 170 (46.58%) | 159  (40.98%) | 161  (42.03%) |
| Yes | 229  (54.01%) | 238  (60.71%) | 199  (51.82%) |  | 195  (53.42%) | 229  (59.02%) | 222  (57.96%) |

**Notes**. Factor 1 is Absence vs Presence of Hesitancy Inducing Message, Factor 2 is Science Supporting Messages: Absence; Science-Supporting Message; or Science-Supporting Message. M_adj_ is adjusted mean from the ANCOVA models, sd is standard deviation, se is standard error. %s are column-wise.

**Table**. Binary Logit Models Predicting Likelihood of Encouraging Others to Vaccinate (MMR) Their Children and Willingness to Send a Pro-Vaccine Letter to One’s State Representative

|  | **Model** |  |  |  |  |  |  |  |  |
| --- | --- | --- | --- | --- | --- | --- | --- | --- | --- |
|  | **Coef.** | **se** | **z** | **p** |  | **Odds Ratio** | **CI**  **2.5** | **CI**  **97.5** |  |
| **Likelihood of Encouraging Others to Vaccinate (MMR) Their Children C1** |  |  |  |  |  |  |  |  |  |
| Vaccine Misinformation | -.84 | (.10) | -8.81 | .00 |  | .43 | .36 | .52 |  |
| Supplemental Sample | .06 | (.12) | .51 | .61 |  | 1.06 | .84 | 1.35 |  |
| Parent | .14 | (.11) | 1.27 | .20 |  | 1.16 | .92 | 1.44 |  |
| Media Exposure (Measles) | .30 | (.06) | 5.47 | .00 |  | 1.36 | 1.22 | 1.51 |  |
| Female | .55 | (.12) | 4.81 | .00 |  | 1.74 | 1.39 | 2.18 |  |
| Trust in Medical Authorities | .83 | (.09) | 9.08 | .00 |  | 2.29 | 1.92 | 2.74 |  |
| Hesitancy Inducing Narrative (Hes) | .02 | (.19) | .12 | .91 |  | 1.02 | .71 | 1.48 |  |
| Science-supporting Message (SSM) | .52 | (.19) | 2.67 | .01 |  | 1.68 | 1.15 | 2.47 |  |
| Science-consistent Narrative (SCN) | .13 | (.19) | .71 | .48 |  | 1.14 | .79 | 1.66 |  |
| Hes X SSM | -.34 | (.28) | -1.21 | .23 |  | .72 | .41 | 1.23 |  |
| Hes X SCN | -.11 | (.27) | -.41 | .68 |  | .90 | .53 | 1.52 |  |
| Pseudo R^2^ | .19 |  |  |  |  |  |  |  |  |
|  |  |  |  |  |  |  |  |  |  |
| **Likelihood of Encouraging Others to Vaccinate (MMR) Their Children C2** | **Coef.** | **se** | **z** | **p** |  | **Odds Ratio** | **CI**  **2.5** | **CI**  **97.5** |  |
| Vaccine Misinformation | -1.37 | (.11) | -12.75 | .00 |  | .25 | .21 | .31 |  |
| Supplemental Sample | -.02 | (.11) | -.23 | .82 |  | .98 | .79 | 1.20 |  |
| Parent | -.08 | (.10) | -.81 | .42 |  | .92 | .76 | 1.12 |  |
| Media Exposure (Measles) | .29 | (.05) | 5.96 | .00 |  | 1.33 | 1.21 | 1.47 |  |
| Female | .43 | (.10) | 4.36 | .00 |  | 1.54 | 1.27 | 1.87 |  |
| Trust in Medical Authorities | .86 | (.10) | 8.80 | .00 |  | 2.36 | 1.95 | 2.86 |  |
| Hesitancy Inducing Narrative (Hes) | .07 | (.17) | .41 | .68 |  | 1.07 | .77 | 1.49 |  |
| Science-supporting Message (SSM) | .52 | (.17) | 3.10 | .00 |  | 1.68 | 1.21 | 2.34 |  |
| Science-consistent Narrative (SCN) | -.02 | (.17) | -.11 | .91 |  | .98 | .71 | 1.36 |  |
| Hes X SSM | -.49 | (.24) | -2.03 | .04 |  | .61 | .38 | .98 |  |
| Hes X SCN | .08 | (.24) | .35 | .73 |  | 1.09 | .68 | 1.74 |  |
| Pseudo R^2^ | .23 |  |  |  |  |  |  |  |  |
|  |  |  |  |  |  |  |  |  |  |
| **Willingness to Send a Pro-Vaccine Letter to State Representative** | **Coef.** | **se** | **z** | **p** |  | **Odds Ratio** | **CI**  **2.5** | **CI**  **97.5** |  |
| Vaccine Misinformation | -.93 | (.09) | -10.16 | .00 |  | .40 | .33 | .47 |  |
| Supplemental Sample | -.15 | (.10) | -1.47 | .14 |  | .86 | .71 | 1.05 |  |
| Parent | -.10 | (.09) | -1.09 | .28 |  | .90 | .75 | 1.09 |  |
| Media Exposure (Measles) | .17 | (.05) | 3.80 | .00 |  | 1.19 | 1.09 | 1.30 |  |
| Female | -.10 | (.09) | -1.09 | .28 |  | .90 | .75 | 1.08 |  |
| Trust in Medical Authorities | .37 | (.08) | 4.50 | .00 |  | 1.45 | 1.24 | 1.71 |  |
| Ideology (Liberal) | .21 | (.04) | 4.87 | .00 |  | 1.23 | 1.13 | 1.34 |  |
| Hesitancy Inducing Narrative (Hes) | -.15 | (.16) | -.98 | .33 |  | .86 | .63 | 1.17 |  |
| Science-supporting Message (SSM) | .37 | (.16) | 2.37 | .02 |  | 1.45 | 1.07 | 1.98 |  |
| Science-consistent Narrative (SCN) | -.16 | (.16) | -1.04 | .30 |  | .85 | .63 | 1.15 |  |
| Hes X SSM | -.01 | (.23) | -.04 | .97 |  | .99 | .64 | 1.55 |  |
| Hes X SCN | .48 | (.23) | 2.13 | .03 |  | 1.62 | 1.04 | 2.53 |  |
| Pseudo R^2^ | .13 |  |  |  |  |  |  |  |  |

**Notes**. Factor 1 is Absence vs Presence of Hesitancy Inducing Message, Factor 2 is Science Messages: Absence, Science-Supportive Message, or Science-Consistent Message. Pseudo R^2^ is based on McFadden calculation. LR denotes likelihood ratio chi-square (χ^2^). Model fit for Likelihood of Encouraging Others to Vaccinate (MMR) Their Children (C1: Coding 1, see Methods section for details of coding): χ2(11) = 468.313, p< .001 (log-likelihood= -997.9179). Model fit for Likelihood of Encouraging Others to Vaccinate (MMR) Their Children (C2: Coding 2, see Methods section for details of coding): χ2(11) = 709.006, p< .001 (log-likelihood= -1237.35). Model fit for Willingness to Send a Pro-Vaccine Letter to State Representative: χ2(12) = 417.714, p< .001 (log-likelihood= -1362.521).

**Table.** Pairwise Contrasts and Simple Effects in Adjusted Means and Predicted Probabilities

|  |  | **MMR Vaccine Risk Evaluations Mean Difference** | **Pro-Vaccine Policy Views Mean Difference** | **Likelihood of Encouraging Others**  **(Coding 1)** | **Likelihood of Encouraging Others**  **(Coding 2)** | **Likelihood of Sending a Pro-Vaccine Letter** |
| --- | --- | --- | --- | --- | --- | --- |
|  |  |  |  |  |  |  |
| **Among** | **Contrast** | M_diff_ (se), p | M_diff_ (se), p | Prob. Difference, χ^2^[df]= chi, p | Prob. Difference χ^2^[df]= chi, p | Prob. Difference, χ^2^[df]= chi, p |
|  |  |  |  |  |  |  |
| Hesitancy Absent | [Expert - No Science Message] | -.17 (.03), p<.001 | .18 (.05), p=.001 | .071, χ^2^(1)= 7.2, p=.008 | .129, χ^2^(1)= 9.6, p=.002 | .090, χ^2^(1)= 5.6, p=.018 |
|  | [Expert - Narrative] | -.15 (.03), p<.001 | .28 (.06), p<.001 | .051, χ^2^(1)= 3.6, p=.058 | .133, χ^2^(1)= 9.7, p=.002 | .13, χ^2^(1)= 10.9, p=.001 |
|  | [Narrative - No Science Message] | -.02 (.03), p=.46 | -.10 (.06), p=.07 | .020, χ^2^(1)= .51, p=.48 | -.004, χ^2^(1)= .012, p=.91 | -.04, χ^2^(1)= 1.1, p=.30 |
|  |  |  |  |  |  |  |
| Hesitancy Present | [Expert - No Science Message] | -.10 (.03), p=.003 | .18 (.06), p=.001 | .028 χ^2^(1)= .7, p=.40 | .009 χ^2^(1)= 2.2, p=.14 | .09 χ^2^(1)= .17, p=.68 |
|  | [Expert - Narrative] | -.04 (.03), p=.24 | .11 (.06), p=.05 | .024, χ^2^(1)= .64, p=.42 | -.008, χ^2^(1)= 5.5, p=.019 | .011, χ^2^(1)= 4.6, p=.033 |
|  | [Narrative - No Science Message] | -.06 (.03), p=.06 | .08 (.06), p=.19 | .004, χ^2^(1)= .097, p=.76 | .017, χ^2^(1)= .001, p=.97 | .079, χ^2^(1)= 3.2, p=.074 |
|  |  |  |  |  |  |  |
| No Science Message | [Hesitancy Absent – Hesitancy Present] | -.02 (.03), p=.51 | .11 (.06), p=.047 | -.003, χ^2^(1)= .013, p=.91 | -.016, χ^2^(1)= .17, p=.68 | .038, χ^2^(1)= .96, p=.33 |
| Expert | [Hesitancy Absent – Hesitancy Present] | -.09 (.03), p=.003 | .11 (.06), p=.049 | .040, χ^2^(1)= 3.8, p=.05 | .104, χ^2^(1)= 7.1, p=.008 | .038, χ^2^(1)= 1.2, p=.28 |
| Narrative | [Hesitancy Absent – Hesitancy Present] | .02 (.03), p=.62 | -.06 (.06), p=.26 | -.013, χ^2^(1)= .33, p=.56 | -.037, χ^2^(1)= .073, p=.79 | -.081, χ^2^(1)= 3.3, p=.068 |
|  |  |  |  |  |  |  |
| Simple Effect for Factor 2 (Science-Supporting Message) | Hesitancy-Inducing Message Absent | F(2,2280) = 17.26, p <. 001 | F(2,2285) = 13.45, p <. 001 | NA | NA | NA |
|  | Hesitancy-Inducing Message Present | F(2,2280) = 4.61, p = .01 | F(2,2285) = 5.43, p = .004 | NA | NA | NA |

**Notes**. For column 1 and 2 in contrasts, adjusted mean differences are presented (F tests), the rest are differences in predicted probabilities (Wald tests). Coding 1, as explained in the Methods section, refers to comparing Somewhat Likely + Very Likely against Not too likely + Not likely at all. Coding 2 refers to the comparing Very Likely against all other response options. Simple effect is shown only for the three-level factor since the two-level factor (Hesitancy Message) results would be the same. P values here not adjusted for Bonferroni correction of 15 pairwise tests.

**Supporting Information C. Preregistration and Itemized Deviations from the Pre-registration**

**Preregistration.** The hypotheses, measures, sample, and analytical strategy of this study were preregistered at *AsPredicted*. The full original pre-registration is provided below. There are a few differences between pre-registration and our final analysis and reporting in the main manuscript. First, we re-numbered and re-organized some hypotheses and research questions in the context of the framing of the manuscript. Second, due to the high number of outcome measures we included in the preregistration, we did not report all analyses in the main report, but provide them in Supporting Information. We have made some changes to analytical approach based on reviewer feedback. Supporting Information provides additional analyses that were mentioned in the preregistration which include a set of additional outcome variables, a moderator analysis, methodological robustness check, and an additional follow up (longitudinal) measurement of outcome measures that showed no effects. These changes are itemized in detail in the second section below, titled Itemized Deviations from the Pre-registration. We believe the results we report and how in the context of the main manuscript, although do not follow the preregistration verbatim, remain truthful to the core of our preregistration. We think that these differences between the original preregistration and final manuscript should be very clearly labeled with explanations, which we provide below. Data is publicly available for other scholars to investigate these other variables further.

**Original Copy of Pre-registration:**

**[Original Pre-registration Text Starts Here]**

*"Measles and MMR vaccine Experiment"*(#21225)

Created: 03/22/2019 10:48 AM (PT)

Author(s)

[name redacted]

1) Have any data been collected for this study already?

It's complicated. We have already collected some data but explain in Question 8 why readers may consider this a valid pre-registration nevertheless.

2) What's the main question being asked or hypothesis being tested in this study?

Hypotheses and Research Questions.

Below, we refer to anti-vaccine and pro-vaccine adjectives while describing our numerous outcome measures. With anti-vaccine, we mean any negative attitude about vaccines, negative beliefs about the efficacy and risks of the vaccine, and disagreements with policy positions that would boost vaccination rates. In contrast, with pro-vaccine, we mean the opposite attributes describing beliefs, and attitudes that view MMR vaccine positively and support more vaccination. See below our outcome variables for details.

H1. Exposure to condition 1 (C1, anti-vax mother) will increase anti-vaccine beliefs, attitudes, and behavior as compared to all the other conditions.

H2. Exposure to C2 (narrative) and C3 (expert view) will decrease anti-vaccine beliefs, attitudes, and behavior as compared to C1 and C6 (control).

H3. Exposure to C4 (anti-vax mother and narrative) and C5 (anti-vax mother and expert view) will decrease anti-vax beliefs, attitudes, and behavior as compared to C1.

Previous literature suggests both narrative and expert view interventions could be effective in reducing misperceptions. Because of this, we propose the following statements as research questions:

RQ1. Is C2 more effective than C3 in decreasing anti-vax beliefs, attitudes, and behavior?

RQ2. Is C4 more effective than C5 in decreasing anti-vax beliefs, attitudes, and behavior?

RQ3. Are C4 AND C5 more effective in decreasing anti-vax beliefs, attitudes, and behavior than C2 AND C3?

H4. Results in H1, H2, and H3 will be moderated by respondents’ misinformation (H4a) and conspiratorial ideas (H4b).

People who hold more misinformed beliefs about vaccines (H4a) and conspiratorial ideas (H4b) will be more likely to hold stronger anti-vax beliefs, attitudes, and behavior when exposed to C1 as compared to people low on misinformation and conspiratorial beliefs.

Similarly, people who hold more misinformed beliefs about vaccines (H4a) and conspiratorial ideas (H4b) will be less likely to change their minds in the pro-vaccine direction when exposed to C2, C3, C4, or C5 as compared to people low on misinformation and conspiratorial beliefs.

H5. We will test all above hypotheses and research questions for potential long lasting effects at the second (follow up) data collection one month after the study as well.

3) Describe the key dependent variable(s) specifying how they will be measured.

Outcome Questions:

Emotions about measles (afraid, disgusted)

Perceived riskiness of MMR vaccine

Perceived complications arising from MMR vaccine

Concern for measles spreading

Effectiveness of MMR vaccine

Relative riskiness of vaccine vs disease

Positivity towards MMR vaccine

Vaccine Knowledge

Policy Views about vaccination

Willingness to share the video on social media

Likelihood to encourage parents to get the vaccine

Send letter to politician

Trust in relevant actors (e.g. state government, pharmaceutical companies, CDC)

4) How many and which conditions will participants be assigned to?

Condition 1 – Anti-vaxx mother

Condition 2 – Personal narratives of affected families

Condition 3 – Dr. Fauci of NIH with infographics

Condition 4 – Condition 1 + Condition 2

Condition 5 – Condition 1 + Condition 3

Condition 6 – CONTROL – Aspirin for heart patients

5) Specify exactly which analyses you will conduct to examine the main question/hypothesis.

We will use ANOVA, ordinary least squares, and ordinal logit regressions to predict the influence of experimental conditions and moderation analyses on the outcome variables listed in Section 6 below. We will use Tukey HSD as the correction method for posthoc multiple comparisons. We are going to analyze two samples detailed below (from a panel and new sample) independently and also combined. These samples may be published separately or together.

6) Describe exactly how outliers will be defined and handled, and your precise rule(s) for excluding observations.

We will exclude those who answer “No” to these question.

1. Were you able to view the entire video? Yes or No

2. Could you clearly hear the sound in the video? Yes or No

7) How many observations will be collected or what will determine sample size?

No need to justify decision, but be precise about exactly how the number will be determined.

There are two different, nationally representative samples collected concurrently between February 28 and March 18. One sample is composed of about 1,800 participants, who are adult residents of the United States. The data was collected by NORC Amerispeak panel. That sample is part of a larger panel study, and it constitutes wave four of that panel.

The second sample is collected by the same survey company and is made up of 1,000 respondents, and this sample is independent and not part of the panel or another study. We did not yet receive the data for this sample.

Also, for the first sample, there will be another data collection one month after the original data collection (in April 2019) which will include some outcome questions listed above. That data collection will measure the potential long lasting effects of the experiment registered here.

8) Anything else you would like to pre-register?

(e.g., secondary analyses, variables collected for exploratory purposes, unusual analyses planned?)

Further explanation for preregistration: We have designed an experiment that is implemented on two different nationally representative samples at the same time. We do not have access to full data of the study as a survey company administers data collection at this point. We have only provisional access to the data from one sample at the time of this preregistration. Also, we have formed our hypotheses and design before data collection. Our hypotheses and analytical procedures are straightforward in the sense that it is clear from our design what our expectations would be based on the previous literature.

Covariates: We will check is groups differ in demographic characteristics at baseline and will include any variable that differs as a covariate in all analyses.

Analyses excluding participants previously exposed to the videos: Using the same methods outlined for the main analyses, we will reanalyze data after excluding participants who report prior exposure to the video contents. These analyses will proceed like the main analyses.

Analyses excluding participants who do not pass manipulation checks: Using the same methods outlined for the main analyses, we will reanalyze data after excluding participants who do not pass the checks for attention to the video contents. These analyses will proceed like the main analyses.

Moderators analyses: Using the same methods outlined for the main analyses, we will conduct audience segmentation analyses on the basis of prior vaccination attitudes, perceived risk, norms, perceived vaccine efficacy, initial level of misinformation, political attitudes, and conspiratorial beliefs. Segmentation effect will be tested by analyzing interactions between treatment and levels of these moderators.

**[Original Pre-registration Text Ends Here]**

**Itemized Deviations from the Pre-registration**

1. Changes about hypotheses and research questions wordings/numbering:
   1. “H1. Exposure to condition C1 (C1, anti-vax mother) will increase anti-vaccine beliefs, attitudes, and behavior as compared to the control condition.” Original comparison was “as compared to all the other conditions”. This does not make much sense as it is a statement of all possible pairwise comparisons. The other hypotheses and research questions already test other pairwise comparisons of interest specifically.
   2. “RQ1. Are C4 AND C5 more effective in decreasing anti-vax beliefs, attitudes, and behavior than C2 AND C3?” The RQ1 reported in the manuscript was originally numbered as RQ3 in the preregistration. It is redundant to test the original RQ1 and RQ2 separately, these differences are already tested in the context of original RQ3. RQ and RQ2, which were: RQ1. Is C2 more effective than C3 in decreasing anti-vax beliefs, attitudes, and behavior? RQ2. Is C4 more effective than C5 in decreasing anti-vax beliefs, attitudes, and behavior?
   3. “H4. Results in H1, H2, and H3 and RQ1 will be moderated by respondents’ misinformation. In brief, we expect those high in misinformation (as opposed to low) to be less responsive to corrections and more responsive to misinformation condition.” The detailed hypothesis numbering on this is unnecessary (H4a, H4b). We are going to look at all pairwise comparisons, there is no need to examine this separately in relation to C1 vs C2 through 5.
2. Regarding H4 moderation for vaccine misinformation, we had the same expectation for another variable, prior conspiratorial beliefs. We do not report this in the main report, but provide results in the relevant section in the Supporting Information
3. We did not report a few outcome variables in the manuscript, the results on these are reported in the relevant section in the Supporting Information. The reason for exclusion in the main report was not due to significance results, as there are significant results in the same direction for these questions too (and sometimes stronger results). The reasons for exclusion from the main report include
   1. (1) theoretical coherence of the paper,
   2. (2) low reliability of some indexes (beliefs about measles, which included four items),
   3. (3) an outcome variable had low validity (“likelihood of sharing the manipulation video on social media,” this variable is not very useful for testing our predictions as we do not know why respondents would share the video (for example, did the respondent share because they believe it or to troll and illustrate and despise misinformation content). Similarly, trust in health related actors is not theoretically relevant since we cannot expect overall trust, as a stable factor, to be influenced by a two minute video clip.
   4. (4) two variable we originally viewed as an outcome variable served actually a de-facto manipulation check (information about vaccines question that asked about the content that varied across videos, so there was very strong significant differences among the conditions, but it does not provide a proper test of our hypotheses, it could rather be best characterized as a manipulation check. Vaccine misperceptions outcome variable, on the other hand, was highly correlated with prior vaccine misinformation predictor and included items that directly referenced to contents of the two of the videos, so again, we were concerned about this outcome variable acting as a de facto manipulation check (informational recognition in line with the video content) instead of constituting a valid outcome variable. Future research should study this as an outcome variable without measuring vaccine misinformation prior to the treatments.
   5. Given the small effects we observed, we did not include the longitudinal effects in the main report, but we mention them in the paper and report full results in the Supporting Information.
4. We had stated in the preregistration that we would use Tukey’s HSD test for pairwise comparisons. The software we used (Stata, version 15.1) allowed us to use Bonferroni correction in the specific models we utilized. However this is not a problem given that Bonferroni is more conservative correction for multiple tests than Tukey’s test.
5. Although we preregistered pair-wise contrasts across our six conditions, during the peer review process, we received useful feedback and made some analytical changes. We grouped the six conditions into two factors as suggested by reviewers and analyzed the results with this factorial approach. Substantive results are mostly unchanged with this new approach. We report all the old analyses in this Appendix as well. Given the interaction of two factors for main hypothesis testing, we dropped the vaccine misinformation moderation from the reporting of main results as well, because 1) this moderator was heavily skewed, 2) we documented null effects in prior analysis and report them in the Appendix, 3) there was no straightforward way to examine the moderation (e.g. ANCOVA assumptions require that a covariate is not included as an interaction term), and 4) the three-way interactions would be hard to interpret in the context of ANCOVA and binary logit models in this design.

**Supporting Information C.** Item by Item Analysis Main Results for Index Scores

Table C1.

| Dependent Variable: Just your best guess, how risky, if at all, do you think the measles, mumps, and rubella (MMR) vaccine is? | | | | | | | |
| --- | --- | --- | --- | --- | --- | --- | --- |
|  | **SS (Type III)** | ***df*** | **MS** | ***F*** | ***P*** | ***η_p_^2^*** |  |
|  |  |  |  |  |  |  |  |
| Parent | 3.078 | 1 | 3.08 | 5.59 | .02 | .002 |  |
| Female | 5.692 | 1 | 5.69 | 10.34 | .00 | .004 |  |
| Control Sample | 0.141 | 1 | .14 | .26 | .61 | .000 |  |
| Trust in Medical Authorities | 48.105 | 1 | 48.11 | 87.34 | .00 | .037 |  |
| Vaccine Misinformation | 139.978 | 1 | 139.98 | 254.16 | .00 | .100 |  |
| Media Exposure (Measles) | 7.309 | 1 | 7.31 | 13.27 | .00 | .006 |  |
| Hesitancy-Inducing Message (Factor 1) | 2.381 | 1 | 2.38 | 4.32 | .04 | .002 |  |
| Science-Supporting Message (Factor 2) | 14.005 | 2 | 7.00 | 12.72 | .00 | .011 |  |
| Factor 1 X Factor 2 | 4.085 | 2 | 2.04 | 3.71 | .03 | .003 |  |
| Residuals | 1263.432 | 2294 | .55 |  |  |  |  |
| R^2^ | 28.7 |  |  |  |  |  |  |

Table C2.

| Dependent Variable: Just your best guess, please indicate how effective, if at all, you think the measles, mumps, and rubella (MMR) vaccine will be at preventing measles among those who get the vaccine in the future? | | | | | | | |
| --- | --- | --- | --- | --- | --- | --- | --- |
|  | **SS (Type III)** | ***df*** | **MS** | ***F*** | ***P*** | ***η_p_^2^*** |  |
|  |  |  |  |  |  |  |  |
| Parent | 0.011 | 1 | .01 | .04 | .84 | .000 |  |
| Female | 1.067 | 1 | 1.07 | 4.06 | .04 | .000 |  |
| Control Sample | 0.192 | 1 | .19 | .73 | .39 | .002 |  |
| Trust in Medical Authorities | 52.503 | 1 | 52.50 | 199.47 | .00 | .055 |  |
| Vaccine Misinformation | 82.35 | 1 | 82.35 | 312.86 | .00 | .132 |  |
| Media Exposure (Measles) | 5.765 | 1 | 5.77 | 21.90 | .00 | .007 |  |
| Hesitancy-Inducing Message (Factor 1) | 0.486 | 1 | .49 | 1.85 | .17 | .000 |  |
| Science-Supporting Message (Factor 2) | 3.596 | 2 | 1.80 | 6.83 | .00 | .006 |  |
| Factor 1 X Factor 2 | 0.738 | 2 | .37 | 1.40 | .25 | .000 |  |
| Residuals | 604.352 | 2296 | .26 |  |  |  |  |
| R^2^ | 0.37 |  |  |  |  |  |  |

Table C3.

| Dependent Variable: Based on what you know, is the measles, mumps, and rubella (MMR) vaccine: Much less risky than catching measles (original code = 1), Slightly less risky than catching measles (2), As risky as catching measles (3), Slightly more risky than catching measles (4), Much more risky than catching measles (5) | | | | | | | |
| --- | --- | --- | --- | --- | --- | --- | --- |
|  | **SS (Type III)** | ***df*** | **MS** | ***F*** | ***P*** | ***η_p_^2^*** |  |
|  |  |  |  |  |  |  |  |
| Parent | 0.264 | 1 | .26 | .49 | .48 | .000 |  |
| Female | 0.157 | 1 | .16 | .29 | .59 | .000 |  |
| Control Sample | 2.629 | 1 | 2.63 | 4.87 | .03 | .002 |  |
| Trust in Medical Authorities | 71.769 | 1 | 71.77 | 132.88 | .00 | .055 |  |
| Vaccine Misinformation | 189.044 | 1 | 189.04 | 350.01 | .00 | .132 |  |
| Media Exposure (Measles) | 8.476 | 1 | 8.48 | 15.69 | .00 | .007 |  |
| Hesitancy-Inducing Message (Factor 1) | 0.107 | 1 | .11 | .20 | .66 | .000 |  |
| Science-Supporting Message (Factor 2) | 7.268 | 2 | 3.63 | 6.73 | .00 | .006 |  |
| Factor 1 X Factor 2 | 0.588 | 2 | .29 | .54 | .58 | .000 |  |
| Residuals | 1238.999 | 2294 | .54 |  |  |  |  |
| R^2^ | 0.35 |  |  |  |  |  |  |

Table C4.

| Dependent Variable: Based on what you know, how positive or negative do you feel about the measles, mumps, and rubella (MMR) vaccine? | | | | | | | |
| --- | --- | --- | --- | --- | --- | --- | --- |
|  | **SS (Type III)** | ***df*** | **MS** | ***F*** | ***P*** | ***η_p_^2^*** |  |
|  |  |  |  |  |  |  |  |
| Parent | 1.244 | 1 | 1.24 | 3.50 | .06 | .002 |  |
| Female | 1.118 | 1 | 1.12 | 3.15 | .08 | .001 |  |
| Control Sample | 0.457 | 1 | .46 | 1.29 | .26 | .001 |  |
| Trust in Medical Authorities | 74.143 | 1 | 74.14 | 208.61 | .00 | .083 |  |
| Vaccine Misinformation | 169.98 | 1 | 169.98 | 478.26 | .00 | .173 |  |
| Media Exposure (Measles) | 8.915 | 1 | 8.92 | 25.08 | .00 | .011 |  |
| Hesitancy-Inducing Message (Factor 1) | 0.209 | 1 | .21 | .59 | .44 | .000 |  |
| Science-Supporting Message (Factor 2) | 5.926 | 2 | 2.96 | 8.34 | .00 | .007 |  |
| Factor 1 X Factor 2 | 2.873 | 2 | 1.44 | 4.04 | .02 | .004 |  |
| Residuals | 814.615 | 2292 | .36 |  |  |  |  |
| R^2^ | 0.44 |  |  |  |  |  |  |

Table C5.

| Dependent Variable: It should be mandatory for parents to vaccinate their children against preventable diseases such as measles, mumps, and rubella. | | | | | | | |
| --- | --- | --- | --- | --- | --- | --- | --- |
|  | **SS (Type III)** | ***df*** | **MS** | ***F*** | ***P*** | ***η_p_^2^*** |  |
|  |  |  |  |  |  |  |  |
| Parent | 2.106 | 1 | 2.11 | 1.92 | .17 | .001 |  |
| Female | 5.227 | 1 | 5.23 | 4.76 | .03 | .002 |  |
| Control Sample | 0.403 | 1 | .40 | .37 | .55 | .000 |  |
| Trust in Medical Authorities | 90.772 | 1 | 90.77 | 82.60 | .00 | .035 |  |
| Vaccine Misinformation | 296.952 | 1 | 296.95 | 270.21 | .00 | .106 |  |
| Media Exposure (Measles) | 22.134 | 1 | 22.13 | 20.14 | .00 | .009 |  |
| Ideology (Liberal) | 10.935 | 1 | 10.94 | 9.95 | .00 | .004 |  |
| Hesitancy-Inducing Message (Factor 1) | 0.244 | 1 | .24 | .22 | .64 | .000 |  |
| Science-Supporting Message (Factor 2) | 27.668 | 2 | 13.83 | 12.59 | .00 | .011 |  |
| Factor 1 X Factor 2 | 5.136 | 2 | 2.57 | 2.34 | .10 | .002 |  |
| Residuals | 2505.659 | 2280 | 1.10 |  |  |  |  |
| R^2^ | 0.31 |  |  |  |  |  |  |

Table C6.

| Dependent Variable: The state I live in should allow parents to choose not to vaccinate their children for medical reasons. | | | | | | | |
| --- | --- | --- | --- | --- | --- | --- | --- |
|  | **SS (Type III)** | ***df*** | **MS** | ***F*** | ***P*** | ***η_p_^2^*** |  |
|  |  |  |  |  |  |  |  |
| Parent | 0.801 | 1 | .80 | .66 | .42 | .000 |  |
| Female | 9.781 | 1 | 9.78 | 8.10 | .00 | .004 |  |
| Control Sample | 3.044 | 1 | 3.04 | 2.52 | .11 | .001 |  |
| Trust in Medical Authorities | 18.981 | 1 | 18.98 | 15.73 | .00 | .007 |  |
| Vaccine Misinformation | 322.992 | 1 | 322.99 | 267.58 | .00 | .105 |  |
| Media Exposure (Measles) | 26.998 | 1 | 27.00 | 22.37 | .00 | .010 |  |
| Ideology (Liberal) | 6.597 | 1 | 6.60 | 5.47 | .02 | .002 |  |
| Hesitancy-Inducing Message (Factor 1) | 1.841 | 1 | 1.84 | 1.53 | .22 | .001 |  |
| Science-Supporting Message (Factor 2) | 25.341 | 2 | 12.67 | 10.50 | .00 | .009 |  |
| Factor 1 X Factor 2 | 1.802 | 2 | .90 | .75 | .47 | .001 |  |
| Residuals | 2753.346 | 2281 | 1.21 |  |  |  |  |
| R^2^ | 0.24 |  |  |  |  |  |  |

Table C7.

| Dependent Variable: The state I live in should allow parents to choose not to vaccinate their children for medical reasons. | | | | | | | |
| --- | --- | --- | --- | --- | --- | --- | --- |
|  | **SS (Type III)** | ***df*** | **MS** | ***F*** | ***P*** | ***η_p_^2^*** |  |
|  |  |  |  |  |  |  |  |
| Parent | 1.484 | 1 | 1.48 | .88 | .35 | .000 |  |
| Female | 2.394 | 1 | 2.39 | 1.42 | .23 | .001 |  |
| Control Sample | 1.732 | 1 | 1.73 | 1.03 | .31 | .000 |  |
| Trust in Medical Authorities | 34.404 | 1 | 34.40 | 20.39 | .00 | .009 |  |
| Vaccine Misinformation | 208.682 | 1 | 208.68 | 123.69 | .00 | .051 |  |
| Media Exposure (Measles) | 0.706 | 1 | .71 | .42 | .52 | .000 |  |
| Ideology (Liberal) | 2.355 | 1 | 2.36 | 1.40 | .24 | .001 |  |
| Hesitancy-Inducing Message (Factor 1) | 17.516 | 1 | 17.52 | 10.38 | .00 | .005 |  |
| Science-Supporting Message (Factor 2) | 11.901 | 2 | 5.95 | 3.53 | .03 | .003 |  |
| Factor 1 X Factor 2 | 0.714 | 2 | .36 | .21 | .81 | .000 |  |
| Residuals | 3848.27 | 2281 | 1.69 |  |  |  |  |
| R^2^ | 0.14 |  |  |  |  |  |  |

Table C8.

| Dependent Variable: The state I live in should allow parents to choose not to vaccinate their children for religious reasons. | | | | | | | |
| --- | --- | --- | --- | --- | --- | --- | --- |
|  | **SS (Type III)** | ***df*** | **MS** | ***F*** | ***P*** | ***η_p_^2^*** |  |
|  |  |  |  |  |  |  |  |
| Parent | 7.028 | 1 | 7.03 | 4.86 | .03 | .002 |  |
| Female | 2.993 | 1 | 2.99 | 2.07 | .15 | .001 |  |
| Control Sample | 2.949 | 1 | 2.95 | 2.04 | .15 | .001 |  |
| Trust in Medical Authorities | 14.072 | 1 | 14.07 | 9.73 | .00 | .004 |  |
| Vaccine Misinformation | 373.077 | 1 | 373.08 | 257.93 | .00 | .102 |  |
| Media Exposure (Measles) | 10.921 | 1 | 10.92 | 7.55 | .01 | .003 |  |
| Ideology (Liberal) | 41.55 | 1 | 41.55 | 28.73 | .00 | .012 |  |
| Hesitancy-Inducing Message (Factor 1) | 1.991 | 1 | 1.99 | 1.38 | .24 | .001 |  |
| Science-Supporting Message (Factor 2) | 24.394 | 2 | 12.20 | 8.43 | .00 | .007 |  |
| Factor 1 X Factor 2 | 9.775 | 2 | 4.89 | 3.38 | .03 | .003 |  |
| Residuals | 3297.834 | 2280 | 1.45 |  |  |  |  |
| R^2^ | 0.23 |  |  |  |  |  |  |

Table C9.

| Dependent Variable: The state I live in should allow parents to choose not to vaccinate their children for personal or philosophical reasons. | | | | | | | |
| --- | --- | --- | --- | --- | --- | --- | --- |
|  | **SS (Type III)** | ***df*** | **MS** | ***F*** | ***P*** | ***η_p_^2^*** |  |
|  |  |  |  |  |  |  |  |
| Parent | 0.784 | 1 | .78 | .66 | .42 | .000 |  |
| Female | 14.891 | 1 | 14.89 | 12.46 | .00 | .005 |  |
| Control Sample | 0.924 | 1 | .92 | .77 | .38 | .000 |  |
| Trust in Medical Authorities | 71.879 | 1 | 71.88 | 60.14 | .00 | .026 |  |
| Vaccine Misinformation | 438.982 | 1 | 438.98 | 367.30 | .00 | .139 |  |
| Media Exposure (Measles) | 25.131 | 1 | 25.13 | 21.03 | .00 | .009 |  |
| Ideology (Liberal) | 18.849 | 1 | 18.85 | 15.77 | .00 | .007 |  |
| Hesitancy-Inducing Message (Factor 1) | 1.391 | 1 | 1.39 | 1.16 | .28 | .001 |  |
| Science-Supporting Message (Factor 2) | 25.965 | 2 | 12.98 | 10.86 | .00 | .009 |  |
| Factor 1 X Factor 2 | 11.273 | 2 | 5.64 | 4.72 | .01 | .004 |  |
| Residuals | 2726.193 | 2281 | 1.20 |  |  |  |  |
| R^2^ | 0.34 |  |  |  |  |  |  |

Table C10.

| Dependent Variable: The state I live in should set aside public funds for free or discounted access to the measles, mumps, and rubella (MMR) vaccines for all children. | | | | | | | |
| --- | --- | --- | --- | --- | --- | --- | --- |
|  | **SS (Type III)** | ***df*** | **MS** | ***F*** | ***P*** | ***η_p_^2^*** |  |
|  |  |  |  |  |  |  |  |
| Parent | 0.002 | 1 | .00 | .00 | .96 | .000 |  |
| Female | 4.073 | 1 | 4.07 | 4.48 | .04 | .002 |  |
| Control Sample | 0.149 | 1 | .15 | .16 | .69 | .000 |  |
| Trust in Medical Authorities | 26.584 | 1 | 26.58 | 29.20 | .00 | .013 |  |
| Vaccine Misinformation | 91.259 | 1 | 91.26 | 100.25 | .00 | .042 |  |
| Media Exposure (Measles) | 2.949 | 1 | 2.95 | 3.24 | .07 | .001 |  |
| Ideology (Liberal) | 98.277 | 1 | 98.28 | 107.96 | .00 | .045 |  |
| Hesitancy-Inducing Message (Factor 1) | 1.155 | 1 | 1.16 | 1.27 | .26 | .001 |  |
| Science-Supporting Message (Factor 2) | 4.944 | 2 | 2.47 | 2.72 | .07 | .002 |  |
| Factor 1 X Factor 2 | 0.863 | 2 | .43 | .47 | .62 | .000 |  |
| Residuals | 2077.359 | 2282 | .91 |  |  |  |  |
| R^2^ | 0.2 |  |  |  |  |  |  |

**Supporting Information D.** Old Results from (Non-factorial) Regression Analysis

***Subsection 1:*** Details for Main Effects

We used ordinary least squares regressions (and binary logit for the dichotomous outcome variable of agreeing to send a letter to state representative) to predict how our video condition, the misinformation moderator (effect modifier), and the covariates explained our dependent measures. Pairwise contrasts were based on the differences between estimated marginal means. We then examined the interaction between conditions and respondents’ vaccine misinformation and tested the nested model fit improvement as well. For each significant result, effect sizes (Cohen d) are also reported. For the binary logit model, estimated marginal means significance are based on z-ratio.

In Table D2, we report pairwise differences between all conditions for each of the four outcome variables, one by one. Table D2 coefficients represent the model estimates, which are the differences between each stated pairs of conditions in their respective units accounting for all covariate variables and multiple comparisons (Bonferroni correction by the total of 15 possible pairwise comparisons amongst six conditions; i.e., our alpha level of .05 divided by 15). Figure 1 presents predicted values of all outcome measures for each condition.

Table D1

| **Test** | **Conditions Compared** |
| --- | --- |
| H1 | C1 vs C6 |
| H2 | C2 vs C1; C2 vs C6; C3 vs C1; C3 vs C6 |
|  |  |
| H3 | C4 vs C1; C5 vs C1 |
|  |  |
| RQ1 | C4 vs C2; C5 vs C3 |
|  |  |
| H4 | H1 through RQ1 by respondent’s baseline vaccine misinformation |

**Results**

We first established the impact of the hesitancy-inducing narrative by comparing it with the baseline (i.e., the control condition). It did not differ from the control message on any outcome measure (Table 3, first row in all four columns; see also Figure D1). H1 was thus unsupported.

**Table D2.** Pairwise Contrasts Between Estimated Marginal Means

|  |  | **MMR Vaccine Risk Perceptions** | |  | **Pro-vaccine Policy Views** | |  | **Encourage Others to Vaccinate Their Children** | |  | **Intention to Send a Pro-vaccine Letter to State Representative** | |
| --- | --- | --- | --- | --- | --- | --- | --- | --- | --- | --- | --- | --- |
|  |  | ***EMM diff*** | ***Effect size*** |  | ***EMM diff*** | ***Effect size*** |  | ***EMM diff*** | ***Effect size*** |  | ***EMM diff*** | ***Effect size*** |
| Ctrl – Hes |  | -.00 |  |  | .02 |  |  | -.00 |  |  | .11 |  |
| Ctrl – SSM |  | .03*** | .35 |  | -.05** | -.25 |  | -.07* | -.24 |  | -.38 |  |
| Ctrl – SCN |  | .00 |  |  | .02 |  |  | -.01 |  |  | .14 |  |
| Ctrl – Hes+SSM |  | .01 |  |  | -.02 |  |  | -.02 |  |  | -.21 |  |
| Ctrl – Hes+SCN |  | .01 |  |  | .00 |  |  | -.01 |  |  | -.18 |  |
| Hes – SSM |  | .03*** | .38 |  | -.08*** | -.36 |  | -.06* | -.22 |  | -.49* | -.44 |
| Hes – SCN |  | .01 |  |  | -.00 |  |  | -.00 |  |  | .03 |  |
| Hes – Hes+SSM |  | .02 |  |  | -.04* | -.22 |  | -.02 |  |  | -.32 |  |
| Hes – Hes+SCN |  | .01 |  |  | -.01 |  |  | -.01 |  |  | -.30 |  |
| SSM – SCN |  | -.03*** | -.30 |  | .07*** | .36 |  | .06* | .22 |  | .52* | .47 |
| SSM – Hes+SSM |  | -.02 |  |  | .03 |  |  | .04 |  |  | .17 |  |
| SSM – Hes+SCN |  | -.02** | -.27 |  | .05** | .28 |  | .05 |  |  | .19 |  |
| SCN – Hes+SSM |  | .01 |  |  | -.04* | -.22 |  | -.02 |  |  | -.36 |  |
| SCN – Hes+SCN |  | .00 |  |  | -.01 |  |  | -.01 |  |  | -.33 |  |
| Hes+SSM – Hes+SCN |  | -.01 |  |  | .03 |  |  | .01 |  |  | .03 |  |

**Notes.** Ctrl – control, Hes – hesitancy-inducing message, SSM – science-supporting statistical message, SCN - science-consistent narrative, + indicates a combined message. * p<0.05, ** p<0.01, *** p<0.001. Bonferroni correction (15 tests) based contrasts between estimated marginal means (EMMs). All outcome variables range from 0 to 1. Contrast directionality: first condition MINUS second condition. Leftmost three models provide p values based on t-ratio statistic while the right-most model provides z-ratio statistic-based p value. Note that for the binary logit model (right-most fourth column), results are given on the log odds ratio (not the response) scale. Effect sizes provide Cohen d and are only shown for significant contrasts (with CI in left three models and asymptotic CIs in the fourth model). The models control for prior vaccine misinformation, media exposure to MMR and measles, parental status, sex, and sample type.

**Figure D1.** Estimated Marginal Means

**
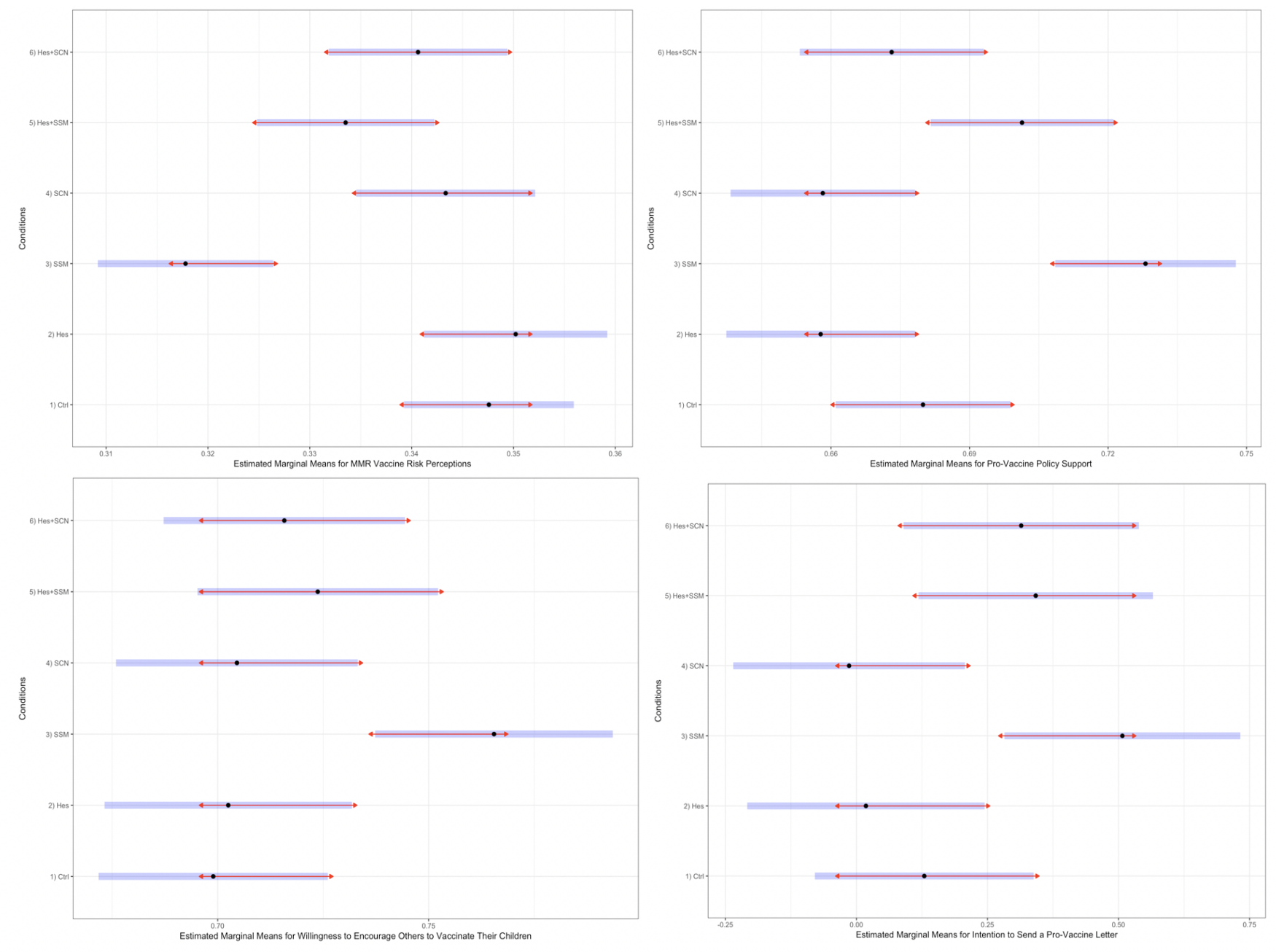
**

**Notes.** The four panels represent the different outcome variables indicated. Estimated (model predicted) marginal means (EMM) for each of the six conditions are shown (all outcome measures range from 0 to 1). The blue bars are the confidence intervals for EMMs while red arrows are for comparisons among them. Overlapping red arrows indicate insignificant findings between any two compared conditions accounting for the Bonferroni adjustment.

We then determined the effect of the science-supporting statistical message and the science-consistent narrative in relation to the control and the hesitancy-inducing narrative condition. Participants exposed to the science-supporting statistical message alone thought that the MMR was less risky than did those in the control (diff = .03, *p* < .001, effect size = .35, row 2 column 1 in Table D2), and expressed higher levels of pro-vaccine policy support (diff = -.05, *p* < .01, effect size = .25, row 2 column 2 in Table D2), and stronger intentions to encourage parents to vaccinate their children (diff = -.07, *p* < .05, effect size = .24, row 2 column 3 in Table D2 and Figure D1). However, there were no differences between the science-consistent narrative and the control condition (row 3, all columns of Table D2).

Second, we compared the science-supporting statistical message and the science-consistent narrative with the hesitancy-inducing narrative. As expected, respondents exposed to the science-supporting statistical message alone exhibited lower levels of perceived risk of the MMR vaccine (diff = .03, *p* < .001, effect size = .38, row 6 column 1 in Table D2), higher levels of pro-vaccine policy support (diff = -.08, *p* < .001, effect size = .36 row 6 column 2 in Table D2), higher intentions to encourage parents to vaccinate their children (diff = -.06, *p* < .05, effect size = .22, row 6 column 3 in Table D2), and higher likelihood of agreeing to send a letter to their state representatives (diff = -.49, *p* < .05, effect size = .44, row 6 column 4 in Table D2 and Figure D1), than did respondents in the hesitancy-inducing narrative condition. The science-consistent narrative did not differ from the hesitancy-inducing narrative in any outcome variables (see Figure 1). In summary, we found partial support for H2, in that the science-supporting statistical message (and not the science-consistent narrative) shifted respondents to more pro-vaccine positions than both the hesitancy-inducing narrative and the control condition.

Next, we examined whether the science-supporting statistical message “corrected” the negative influence of the vaccine-inducing narrative (H3). Although the hesitancy-inducing narrative alone had no influence to correct (i.e., no difference between the vaccine-hesitancy narrative and the control condition), this hypothesis provides a test of the influence of the science-supporting statistical message. We found that when the hesitancy-inducing narrative was followed by the science-supporting statistical message or the science-consistent narrative, neither of these combined conditions led to any changes in outcome variables compared to either the hesitancy-inducing narrative alone or the control condition alone (rows 3, 4, 7, and 8 in Table D2). The only exception was that the combined condition of hesitancy-inducing narrative and science-supporting statistical message increased pro-vaccine policy support as compared to the hesitancy-inducing narrative alone (diff = -.04, p < .05, effect size = .22, row 8 column 2 in Table D2). These patterns are evident in all four panels in Figure D1. Overall, no support for H3 was found.

For RQ1, we found that participants in the combined condition of the hesitancy-inducing narrative with the science-supporting statistical message did not significantly differ from those in the science-supporting statistical message alone (row 11 in all four columns in Table D2). Similarly, no difference was found between the science-consistent narrative alone and the combined condition of the hesitancy-inducing narrative with the science-consistent narrative.

Finally, analyses showed no significant interactions between respondents’ prior vaccine misinformation and experimental exposure on any of the outcome variables (H4 unsupported). The vaccine misinformation distribution was highly positively skewed with very few respondents having high levels of misinformation (31% of the sample scored zero for vaccine misinformation). Nevertheless, for stratification purposes, we present separate results for participants with low and high misinformation levels further below in Subsection 2.

**Table D3.** Manuscript outcome variables’ full model results

|  | **MMR Vaccine Risk Perceptions** | | |  | **Pro-vaccine Policy Views** | | |  | **Encourage Others to Vaccinate Their Children** | | | |  | | **Intention to Send a Pro-vaccine Letter to State Representative** | | |
| --- | --- | --- | --- | --- | --- | --- | --- | --- | --- | --- | --- | --- | --- | --- | --- | --- | --- |
|  | **Coef.** |  | **se** |  | **Coef.** |  | **se** |  | | **Coef.** |  | **se** |  | **Coef.** | |  | **se** |
| (Intercept) | .28 | *** | (.01) |  | .78 | *** | (.01) |  | | .74 | *** | (.02) |  | .78 | | *** | (.15) |
| Hesitancy-Inducing Narrative | .00 |  | (.01) |  | -.02 |  | (.01) |  | | .00 |  | (.02) |  | -.11 | |  | (.16) |
| Science-Supporting Message (SSM) | -.03 | *** | (.01) |  | .05 | *** | (.01) |  | | .07 | *** | (.02) |  | .38 | | * | (.16) |
| Science-Consistent Narrative (SCN) | .00 |  | (.01) |  | -.02 |  | (.01) |  | | .01 |  | (.02) |  | -.14 | |  | (.15) |
| Hesitancy + SSM | -.01 | * | (.01) |  | .02 |  | (.01) |  | | .02 |  | (.02) |  | .21 | |  | (.15) |
| Hesitancy + SCN | -.01 |  | (.01) |  | -.01 |  | (.01) |  | | .02 |  | (.02) |  | .18 | |  | (.16) |
| Nonpanel Sample Respondent | .01 | ** | (.00) |  | -.01 |  | (.01) |  | | .00 |  | (.01) |  | -.17 | | † | (.10) |
| Parent | .01 | ** | (.00) |  | -.02 | ** | (.01) |  | | .00 |  | (.01) |  | -.19 | | * | (.09) |
| Media exposure to MMR | -.01 | *** | (.00) |  | .02 | *** | (.00) |  | | .05 | *** | (.01) |  | .22 | | *** | (.04) |
| Female | .00 |  | (.00) |  | .02 | * | (.01) |  | | .05 | *** | (.01) |  | -.09 | |  | (.09) |
| Vaccine misinformation | .12 | *** | (.00) |  | -.21 | *** | (.01) |  | | -.22 | *** | (.01) |  | -1.19 | | *** | (.08) |
| **N** | 2,314 | | |  | 2,311 | | |  | 2,303 | | | |  | | 2,299 | | |
| **R-square** | .48 | | |  | .36 | | |  | | .26 | | |  | .13 | | | |

**Notes**. † p<.10, * p<0.05, ** p<0.01, *** p<0.001. R^2^ for the rightmost model (Intention to Send a Pro-vaccine Letter to State Representative) is McFadden statistic.

***Subsection 2:*** Details for Vaccine Misinformation Moderation

**Table D4.** Vaccine misinformation interactions for the manuscript outcome variables

|  | **MMR Vaccine Risk Perceptions** | | |  | **Pro-vaccine Policy Views** | | |  | **Encourage Others to Vaccinate Their Children** | | |  | **Intention to Send a Pro-vaccine Letter to State Representative** | | |
| --- | --- | --- | --- | --- | --- | --- | --- | --- | --- | --- | --- | --- | --- | --- | --- |
|  | **Coef.** |  | **se** |  | **Coef.** |  | **se** |  | **Coef.** |  | **se** |  | **Coef.** |  | **se** |
| (Intercept) | .28 | *** | (.01) |  | .78 | *** | (.02) |  | .75 | *** | (.02) |  | .76 | *** | (.18) |
| Hesitancy-Inducing Narrative | .00 |  | (.01) |  | -.02 |  | (.02) |  | .00 |  | (.03) |  | -.15 |  | (.22) |
| Science-Supporting Message (SSM) | -.02 | ** | (.01) |  | .05 | ** | (.02) |  | .05 | † | (.03) |  | .49 | * | (.23) |
| Science-Consistent Narrative (SCN) | .00 |  | (.01) |  | -.01 |  | (.02) |  | .02 |  | (.03) |  | -.15 |  | (.22) |
| Hesitancy + SSM | .00 |  | (.01) |  | .01 |  | (.02) |  | -.02 |  | (.03) |  | .30 |  | (.23) |
| Hesitancy + SCN | .00 |  | (.01) |  | -.01 |  | (.02) |  | .00 |  | (.03) |  | .12 |  | (.23) |
| Vaccine misinformation | .13 | *** | (.01) |  | -.20 | *** | (.02) |  | -.24 | *** | (.02) |  | -1.17 | *** | (.19) |
| Nonpanel Sample Respondent | .01 | ** | (.00) |  | -.01 |  | (.01) |  | .00 |  | (.01) |  | -.17 | † | (.10) |
| Parent | .01 | ** | (.00) |  | -.02 | ** | (.01) |  | .00 |  | (.01) |  | -.19 | * | (.09) |
| Media exposure to MMR | -.01 | *** | (.00) |  | .02 | *** | (.00) |  | .05 | *** | (.01) |  | .22 | *** | (.04) |
| Female | .00 |  | (.00) |  | .02 | * | (.01) |  | .05 | *** | (.01) |  | -.09 |  | (.09) |
| Hes X Vaccine misinformation | .00 |  | (.01) |  | -.01 |  | (.02) |  | .01 |  | (.03) |  | .07 |  | (.28) |
| SSM X Vaccine misinformation | -.01 |  | (.01) |  | -.01 |  | (.02) |  | .02 |  | (.03) |  | -.16 |  | (.26) |
| SCN X Vaccine misinformation | -.01 |  | (.01) |  | -.03 |  | (.02) |  | -.02 |  | (.03) |  | .02 |  | (.27) |
| Hes+SSM X Vaccine misinformation | -.03 | ** | (.01) |  | .02 |  | (.02) |  | .07 | * | (.03) |  | -.13 |  | (.27) |
| Hes+SCN X Vaccine misinformation | -.01 |  | (.01) |  | .00 |  | (.02) |  | .03 |  | (.03) |  | .11 |  | (.27) |
| **N** | 2,314 | | |  | 2,311 | | |  | 2,303 | | |  | 2,299 | | |
| **R^2^** | .48 | | |  | .36 | | |  | .26 | | |  | .13 | | |
| **F-change** | F(5)=1.99† | | |  | F(5)=1.16 | | |  | F(5)=1.96† | | |  | 1.6 | | |

**Notes**. † p<.10, * p<0.05, ** p<0.01, *** p<0.001. F-changes compared against the model without the interaction terms. The rightmost model (Intention to Send a Pro-vaccine Letter to State Representative) has a nonsignificant improvement in residual deviances (comparable to F-change) and the R^2^ reported for this model is McFadden statistic.

Below, the left four panels represent the different outcome variables when models predict only among respondents with low prior vaccine misinformation (those who scored at median or below in the misinformation index, N = 1,339) while the right four panels show the results for those with high prior vaccine misinformation (scoring higher than median in the misinformation index, N = 975). Note that the misinformation moderation is not significant, these plots are shown to give an idea of the pattern that results seemed to be more pronounced (differences between conditions) among those with low prior vaccine misinformation.

**Figure D2.** Respondent Vaccine Misinformation Trends (see Figure notes below)


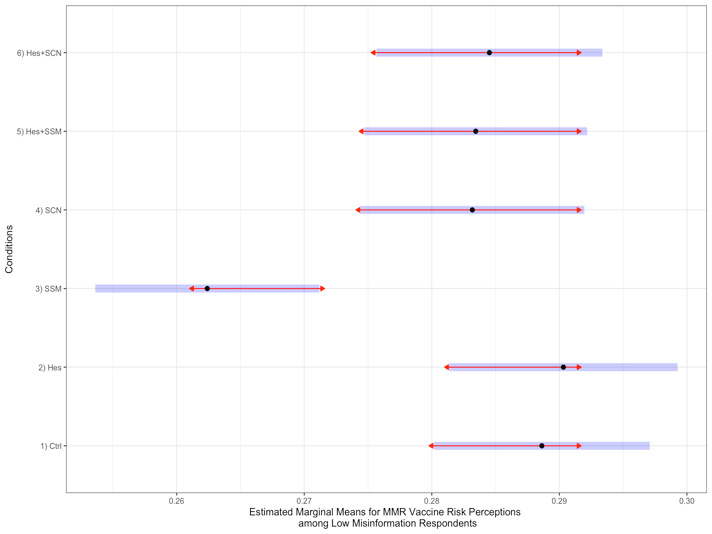

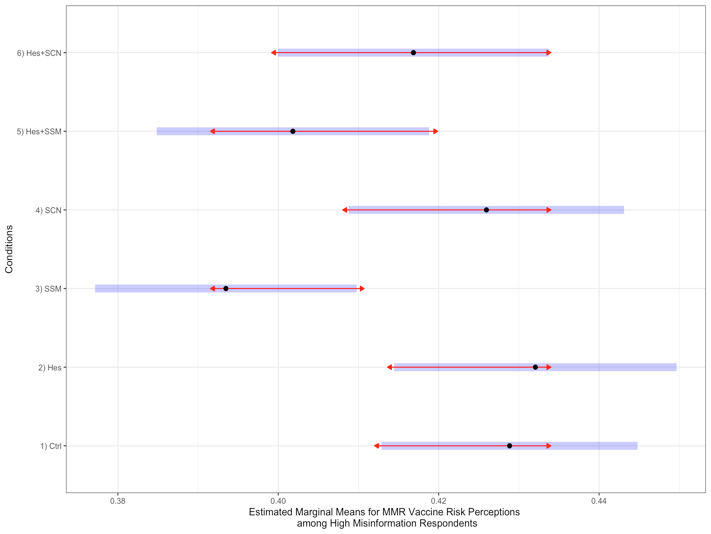


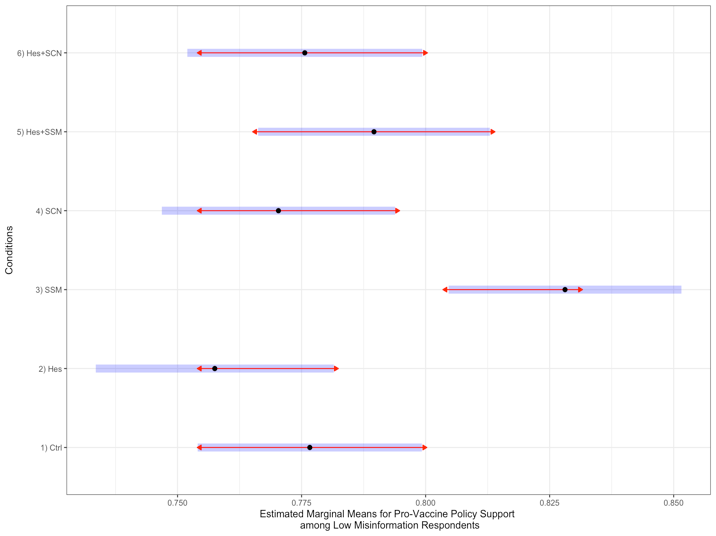

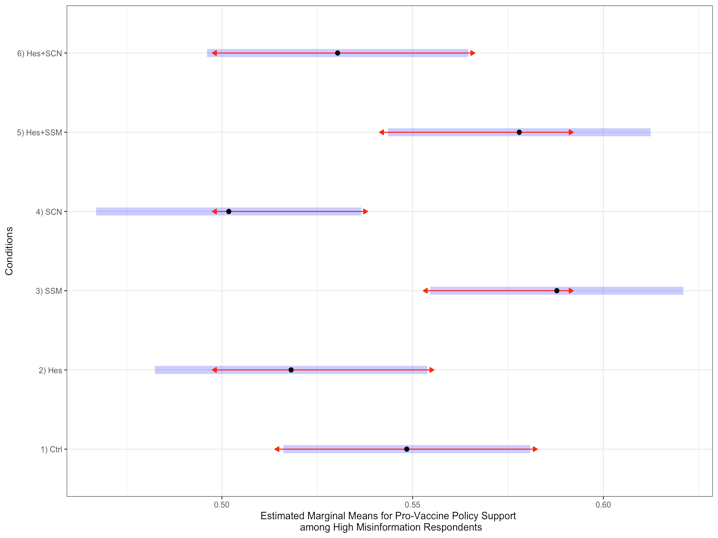


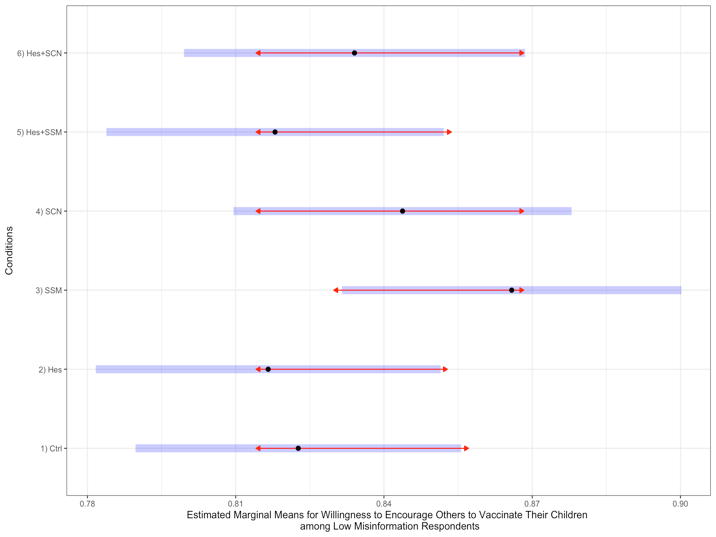

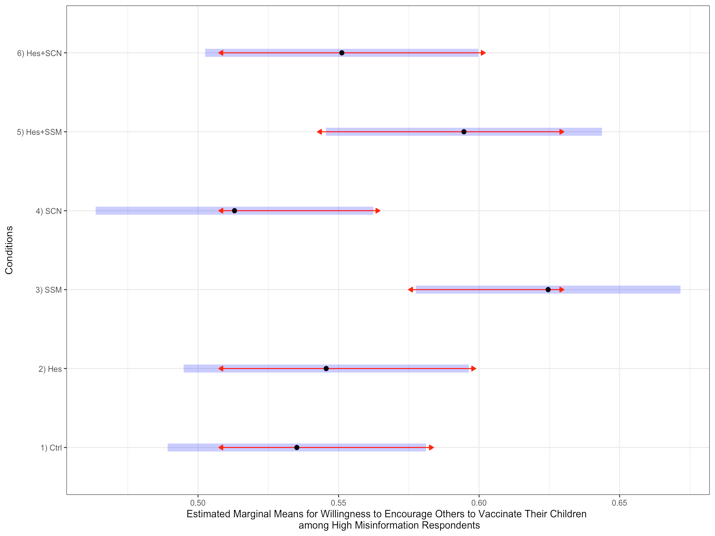


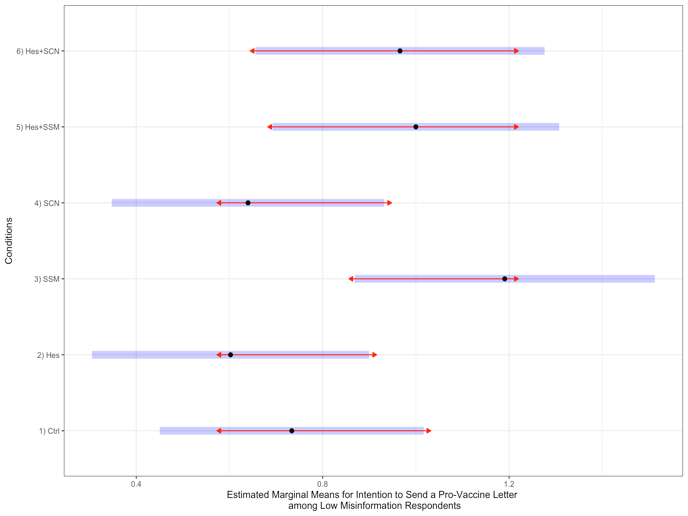

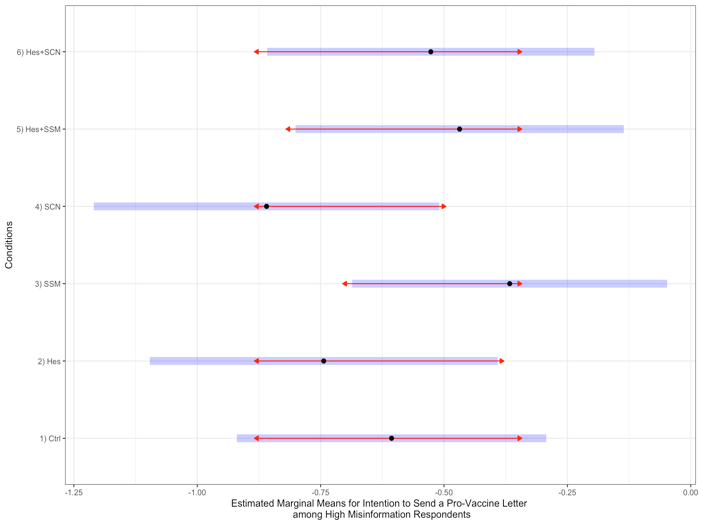


**Notes.** Estimated (model predicted) marginal means (EMM) for each of the six conditions are shown (all outcome measures range from 0 to 1). The blue bars are the confidence intervals for EMMs while red arrows are for comparisons among them. Overlapping red arrows indicate insignificant findings between any two compared conditions accounting for the Bonferroni adjustment.

***Subsection 3:*** Odds Ratios for Binary Logit Model

**Table D5**. Odds ratios for the binary outcome variable – Intention to Send a Pro-vaccine Letter to State Representative

|  | **Odds Ratio** | **2.50%** | **97.50%** |
| --- | --- | --- | --- |
| (Intercept) | 2.18 | 1.63 | 2.93 |
| Hesitancy-Inducing Narrative | .89 | .66 | 1.21 |
| Science-Supporting Message (SSM) | 1.46 | 1.08 | 1.98 |
| Science-Consistent Narrative (SCN) | .87 | .64 | 1.17 |
| Hesitancy + SSM | 1.24 | .91 | 1.68 |
| Hesitancy + SCN | 1.20 | .89 | 1.63 |
| Nonpanel Sample Respondent | .84 | .70 | 1.02 |
| Parent | .83 | .69 | .99 |
| Media exposure to MMR | 1.24 | 1.14 | 1.36 |
| Female | .91 | .76 | 1.09 |
| Vaccine misinformation | .30 | .26 | .36 |

**Notes**. For example, being exposed to SSM as opposed to control increases the odds of agreeing to send a letter by a factor of 1.46. The intercept’s OR should not be interpreted, it is not meaningful.

***Subsection 4:*** Results Including Respondent Ideology for the Two Policy Related Outcome Variables

**Table D6**. Models that control for ideology for policy-related outcome variables

|  | **Pro-vaccine Policy Views** | | |  | **Intention to Send a Pro-vaccine Letter to State Representative** | | |
| --- | --- | --- | --- | --- | --- | --- | --- |
|  | **Coef.** |  | **se** |  | **Coef.** |  | **se** |
| (Intercept) | .71 | *** | (.02) |  | .15 |  | (.20) |
| Hesitancy-Inducing Narrative | -.03 | * | (.01) |  | -.16 |  | (.16) |
| Science-Supporting Message (SSM) | .05 | *** | (.01) |  | .37 | * | (.16) |
| Science-Consistent Narrative (SCN) | -.03 | † | (.01) |  | -.17 |  | (.16) |
| Hesitancy + SSM | .02 |  | (.01) |  | .21 |  | (.16) |
| Hesitancy + SCN | -.01 |  | (.01) |  | .17 |  | (.16) |
| Nonpanel Sample Respondent | -.01 |  | (.01) |  | -.17 | † | (.10) |
| Parent | -.02 | * | (.01) |  | -.14 |  | (.09) |
| Media exposure to MMR | .02 | *** | (.00) |  | .20 | *** | (.05) |
| Female | .02 | * | (.01) |  | -.12 |  | (.09) |
| Vaccine misinformation | -.20 | *** | (.01) |  | -1.13 | *** | (.08) |
| Ideology (Liberal) | .02 | *** | (.00) |  | .21 | *** | (.04) |
| **N** | 2,299 | | |  | 2,294 | | |
| **R^2^** | .37 | | |  | .14 | | |

† p<.10, * p<0.05, ** p<0.01, *** p<0.001. The rightmost model (Intention to Send a Pro-vaccine Letter to State Representative) has R^2^ reported in McFadden statistic.

***Subsection 5:*** Methodological Check – Device of Survey Respondent (PC vs Smartphone/Tablet) and Sample Type

Some respondents took the survey experiment on their phones while some others completed on computers. In the panel sample, 766 respondents took the survey over a desktop computer (42.5%) while 807 (44.8%) on a smartphone and 109 on a tablet (6%). In the control sample, 324 respondents took the survey over a desktop computer (32.2%) while 496 (49.3%) on a smartphone and 44 on a tablet (4.4%). The device with which the respondent participated in the survey experiment might matter for our study, especially given that our manipulation was audio-visual (news videos). Indeed, recent research shows that people process news videos differentially in large computer screens vs smaller smart phone screens (Dunaway and Soroka, 2019).* Hence we controlled for this and did not find a mode effect for three outcome variables, but there was a significant effect for MMR vaccine risk perceptions index outcome variable. Such that, those who watched the experiment on their smartphones had greater risk perceptions about MMR. We delved into analyzing why this could be the case, and our analysis showed that demographic variables did not explain this difference. In fact, independent of the experimental module, we found that smartphone respondents of the survey had greater risk perceptions about the vaccine, controlling for demographics. We do not have an explanation about why this could be the case, one potential explanation could lie with the recruitment strategies of the survey company. Still, we also looked at interaction between sample type and experimental conditions and none of those had significant effects, showing that sample type did not interfere with the update of experimental stimuli.

* Johanna Dunaway & Stuart Soroka (2019) Smartphone-size screens constrain cognitive access to video news stories, Information, Communication & Society,DOI: [10.1080/1369118X.2019.1631367](https://doi.org/10.1080/1369118X.2019.1631367)

**Table D7**. Models with device of use (computer vs smartphone/tablet)

|  | **MMR Vaccine Risk Perceptions** | | |  | **Pro-vaccine Policy Views** | | |  | **Encourage Others to Vaccinate Their Children** | | |  | **Intention to Send a Pro-vaccine Letter to State Representative** | | |
| --- | --- | --- | --- | --- | --- | --- | --- | --- | --- | --- | --- | --- | --- | --- | --- |
|  | **Coef.** |  | **se** |  | **Coef.** |  | **se** |  | **Coef.** |  | **se** |  | **Coef.** |  | **se** |
| (Intercept) | .28 | *** | (.01) |  | .78 | *** | (.02) |  | .75 | *** | (.02) |  | .85 | *** | (.19) |
| Hesitancy-Inducing Narrative | .00 |  | (.01) |  | -.03 |  | (.02) |  | .02 |  | (.03) |  | -.03 |  | (.22) |
| Science-Supporting Message (SSM) | -.03 | *** | (.01) |  | .06 | ** | (.02) |  | .07 | * | (.03) |  | .43 | † | (.23) |
| Science-Consistent Narrative (SCN) | -.01 |  | (.01) |  | -.02 |  | (.02) |  | -.01 |  | (.03) |  | -.16 |  | (.22) |
| Hesitancy + SSM | -.01 |  | (.01) |  | .01 |  | (.02) |  | .02 |  | (.03) |  | .06 |  | (.23) |
| Hesitancy + SCN | .00 |  | (.01) |  | -.02 |  | (.02) |  | -.01 |  | (.03) |  | .13 |  | (.22) |
| Phone | .01 |  | (.01) |  | .00 |  | (.02) |  | -.01 |  | (.03) |  | -.17 |  | (.21) |
| Nonpanel Sample Respondent | .01 | * | (.00) |  | -.01 |  | (.01) |  | .00 |  | (.01) |  | -.16 |  | (.10) |
| Parent | .01 | * | (.00) |  | -.02 | ** | (.01) |  | .00 |  | (.01) |  | -.15 |  | (.09) |
| Media exposure to MMR | -.01 | *** | (.00) |  | .02 | *** | (.00) |  | .05 | *** | (.01) |  | .21 | *** | (.05) |
| Female | .00 |  | (.00) |  | .02 | * | (.01) |  | .05 | *** | (.01) |  | -.07 |  | (.09) |
| Vaccine misinformation | .12 | *** | (.00) |  | -.21 | *** | (.01) |  | -.22 | *** | (.01) |  | -1.19 | *** | (.08) |
| Hes X Phone | .01 |  | (.01) |  | .02 |  | (.03) |  | -.04 |  | (.04) |  | -.18 |  | (.31) |
| SSM X Phone | .00 |  | (.01) |  | -.02 |  | (.03) |  | -.01 |  | (.04) |  | -.11 |  | (.31) |
| SCN X Phone | .00 |  | (.01) |  | -.01 |  | (.03) |  | .04 |  | (.04) |  | .03 |  | (.31) |
| Hes+SSM X Phone | .00 |  | (.01) |  | .01 |  | (.03) |  | .01 |  | (.04) |  | .29 |  | (.31) |
| Hes+SCN X Phone | -.01 |  | (.01) |  | .02 |  | (.03) |  | .05 |  | (.04) |  | .10 |  | (.31) |
| **N** | 2314 | | |  | 2311 | | |  | 2303 | | |  | 2307 | | |
| **R^2^** | .48 | | |  | .36 | | |  | .26 | | |  | .13 | | |
| **F-test** | F(5)=.82 | | |  | F(5)=.76 | | |  | F(5)=1.14 | | |  | 2.65 | | |

**Notes**. † p<.10, * p<0.05, ** p<0.01, *** p<0.001. R^2^ for the rightmost model (Intention to Send a Pro-vaccine Letter to State Representative) is McFadden statistic. F-changes compared against the model without the interaction terms. The rightmost model (Intention to Send a Pro-vaccine Letter to State Representative) has a nonsignificant improvement in residual deviances (comparable to F-change). Phone (0=PC, 1=smartphone/tablet)

Our full sample comes from two simultaneously conducted surveys. The larger sample is a part of a panel study, it is the fourth wave of the panel. The second sample is a concurrent new sample and is composed of fresh respondents recruited by the survey company (also probability-based nationally representative sample). The results reported in the main paper are obtained from this aggregated sample. This brings up the question whether sample type mattered for the experimental effects we observed. In order to answer this question, we did two things. We first run the models on each sample separately and observed whether there was any reverse pattern of findings. We did not find any reverse pattern of results across samples. However we found that our smaller sample (the clean refresh sample) there are less number of significant coefficients. Second, we examined whether there was an interaction effect between experimental manipulation and sample type. This analysis would allow us to test whether in one of the samples respondents in one condition might have reacted differently than the respondents in the same condition in the other sample. We found no such interaction effects. Overall we conclude that both samples produced similar results and there should not be a methodological bias that interferes with the substantive findings.

**Table D8**. Sample Type Analysis

|  | **MMR Vaccine Risk Perceptions** | | |  | | **Pro-vaccine Policy Views** | | | |  | | **Encourage Others to Vaccinate Their Children** | | | |  | | **Intention to Send a Pro-vaccine Letter to State Representative** | | | |
| --- | --- | --- | --- | --- | --- | --- | --- | --- | --- | --- | --- | --- | --- | --- | --- | --- | --- | --- | --- | --- | --- |
|  | **Coef.** |  | **se** |  | **Coef.** | |  | **se** |  | | **Coef.** | |  | **se** |  | | **Coef.** | |  | **se** |  |
| (Intercept) | .28 | *** | (.01) |  | .78 | | *** | (.01) |  | | .74 | | *** | (.02) |  | | .81 | | *** | (.17) |  |
| Hesitancy-Inducing Narrative | .01 |  | (.01) |  | -.02 | |  | (.02) |  | | .01 | |  | (.02) |  | | -.19 | |  | (.19) |  |
| Science-Supporting Message (SSM) | -.03 | *** | (.01) |  | .05 | | ** | (.02) |  | | .07 | | ** | (.02) |  | | .50 | | * | (.19) |  |
| Science-Consistent Narrative (SCN) | .00 |  | (.01) |  | -.01 | |  | (.02) |  | | .00 | |  | (.02) |  | | -.29 | |  | (.19) |  |
| Hesitancy + SSM | -.02 | * | (.01) |  | .02 | |  | (.02) |  | | .03 | |  | (.02) |  | | .23 | |  | (.19) |  |
| Hesitancy + SCN | -.01 |  | (.01) |  | -.02 | |  | (.02) |  | | .01 | |  | (.02) |  | | .14 | |  | (.19) |  |
| Sample | .01 |  | (.01) |  | -.01 | |  | (.02) |  | | -.01 | |  | (.03) |  | | -.24 | |  | (.22) |  |
| Parent | .01 | ** | (.00) |  | -.02 | | ** | (.01) |  | | .00 | |  | (.01) |  | | -.19 | | * | (.09) |  |
| Media exposure to MMR | -.01 | *** | (.00) |  | .02 | | *** | (.00) |  | | .05 | | *** | (.01) |  | | .22 | | *** | (.04) |  |
| Female | .00 |  | (.00) |  | .02 | | * | (.01) |  | | .05 | | *** | (.01) |  | | -.08 | |  | (.09) |  |
| Vaccine misinformation | .12 | *** | (.00) |  | -.21 | | *** | (.01) |  | | -.22 | | *** | (.01) |  | | -1.19 | | *** | (.08) |  |
| Hes X Sample | -.01 |  | (.01) |  | .00 | |  | (.03) |  | | -.01 | |  | (.04) |  | | .25 | |  | (.33) |  |
| SSM X Sample | .01 |  | (.01) |  | -.02 | |  | (.03) |  | | .00 | |  | (.04) |  | | -.35 | |  | (.32) |  |
| SCN X Sample | .00 |  | (.01) |  | -.02 | |  | (.03) |  | | .03 | |  | (.04) |  | | .46 | |  | (.33) |  |
| Hes+SSM X Sample | .00 |  | (.01) |  | .01 | |  | (.03) |  | | -.03 | |  | (.04) |  | | -.04 | |  | (.32) |  |
| Hes+SCN X Sample | .00 |  | (.01) |  | .03 | |  | (.03) |  | | .03 | |  | (.04) |  | | .12 | |  | (.33) |  |
| **N** | 2315 | | |  | 2312 | | | |  | | 2304 | | | |  | | 2307 | | | |  |
| **R^2^** | .48 | | |  | .36 | | | |  | | .26 | | | |  | | .13 | | | |  |
| **F-test** | F(5)=.32 | | |  | F(5)=.68 | | | |  | | F(5)=.48 | | | |  | | 6.6 | | | |  |

**Notes**. † p<.10, * p<0.05, ** p<0.01, *** p<0.001. R^2^ for the rightmost model (Intention to Send a Pro-vaccine Letter to State Representative) is McFadden statistic. F-changes compared against the model without the interaction terms. The rightmost model (Intention to Send a Pro-vaccine Letter to State Representative) has a nonsignificant improvement in residual deviances (comparable to F-change). Sample (0=panel, 1=non-panel).

***Subsection 6.*** Other Results for Preregistered Tests (Other outcome measures, additional moderator, and the longitudinal effects)

**Table D9**. Other outcome variables in the preregistration

|  | **Measles Risk Perceptions** | | |  | **Likelihood of Sharing the Experiment Video** | | |  | **Trust in Health Actors** | | |  | **Vaccine Misperceptions** | | |
| --- | --- | --- | --- | --- | --- | --- | --- | --- | --- | --- | --- | --- | --- | --- | --- |
| ***Main effect models*** |  |  |  |  |  |  |  |  |  |  |  |  |  |  |  |
|  | **Coef.** |  | **se** |  | **Coef.** |  | **se** |  | **Coef.** |  | **se** |  | **Coef.** |  | **se** |
| (Intercept) | .42 | *** | (.01) |  | .22 | *** | (.02) |  | .69 | *** | (.01) |  | .38 | *** | (.01) |
| Hesitancy-Inducing Narrative | .03 | † | (.01) |  | -.11 | *** | (.03) |  | .00 |  | (.01) |  | -.01 |  | (.01) |
| Science-Supporting Message (SSM) | .09 | *** | (.01) |  | .12 | *** | (.02) |  | .03 | ** | (.01) |  | -.05 | *** | (.01) |
| Science-Consistent Narrative (SCN) | .06 | *** | (.01) |  | .05 | * | (.02) |  | .01 |  | (.01) |  | -.05 | *** | (.01) |
| Hesitancy + SSM | .08 | *** | (.01) |  | .05 | † | (.03) |  | .01 |  | (.01) |  | -.12 | *** | (.01) |
| Hesitancy + SCN | .08 | *** | (.01) |  | .01 |  | (.03) |  | .03 | ** | (.01) |  | -.11 | *** | (.01) |
| Nonpanel Sample Respondent | .00 |  | (.01) |  | -.01 |  | (.02) |  | -.02 | * | (.01) |  | .02 | *** | (.01) |
| Parent | .02 | † | (.01) |  | .05 | ** | (.01) |  | -.02 | * | (.01) |  | .00 |  | (.01) |
| Media exposure to MMR | .03 | *** | (.00) |  | .01 | † | (.01) |  | .01 | ** | (.00) |  | -.02 | *** | (.00) |
| Female | .06 | *** | (.01) |  | .07 | *** | (.01) |  | -.01 |  | (.01) |  | -.01 |  | (.01) |
| Vaccine misinformation | -.05 | *** | (.01) |  | .02 |  | (.01) |  | -.14 | *** | (.01) |  | .10 | *** | (.00) |
| **N** | 2314 | | |  | 2119 | | |  | 2312 | | |  | 2310 | | |
| **R^2^** | .11 | | |  | .06 | | |  | .24 | | |  | .26 | | |
| ***Models with interaction terms*** |  |  |  |  |  |  |  |  |  |  |  |  |  |  |  |
| (Intercept) | .41 | *** | (.02) |  | .21 | *** | (.03) |  | .69 | *** | (.01) |  | .38 | *** | (.01) |
| Hesitancy-Inducing Narrative | .03 |  | (.02) |  | -.16 | *** | (.04) |  | .01 |  | (.02) |  | -.02 |  | (.01) |
| Science-Supporting Message (SSM) | .10 | *** | (.02) |  | .16 | *** | (.03) |  | .04 | * | (.02) |  | -.06 | *** | (.01) |
| Science-Consistent Narrative (SCN) | .08 | *** | (.02) |  | .10 | ** | (.03) |  | .02 |  | (.02) |  | -.05 | ** | (.01) |
| Hesitancy + SSM | .09 | *** | (.02) |  | .06 | † | (.03) |  | .00 |  | (.02) |  | -.13 | *** | (.01) |
| Hesitancy + SCN | .08 | *** | (.02) |  | .01 |  | (.04) |  | .02 |  | (.02) |  | -.13 | *** | (.01) |
| Vaccine misinformation | -.04 | * | (.02) |  | .03 |  | (.03) |  | -.14 | *** | (.01) |  | .09 | *** | (.01) |
| Nonpanel Sample Respondent | .00 |  | (.01) |  | -.01 |  | (.02) |  | -.02 | * | (.01) |  | .02 | *** | (.01) |
| Parent | .02 | * | (.01) |  | .05 | *** | (.01) |  | -.02 | * | (.01) |  | .00 |  | (.01) |
| Media exposure to MMR | .04 | *** | (.00) |  | .01 | † | (.01) |  | .01 | *** | (.00) |  | -.02 | *** | (.00) |
| Female | .06 | *** | (.01) |  | .07 | *** | (.01) |  | -.01 |  | (.01) |  | -.01 | † | (.01) |
| Hes X Vaccine misinformation | .00 |  | (.02) |  | .10 | * | (.04) |  | -.02 |  | (.02) |  | .01 |  | (.02) |
| SSM X Vaccine misinformation | -.02 |  | (.02) |  | -.07 | † | (.04) |  | -.01 |  | (.02) |  | .01 |  | (.02) |
| SCN X Vaccine misinformation | -.04 | † | (.02) |  | -.07 | † | (.04) |  | -.01 |  | (.02) |  | .00 |  | (.02) |
| Hes+SSM X Vaccine misinformation | -.01 |  | (.02) |  | -.02 |  | (.04) |  | .03 |  | (.02) |  | .02 |  | (.02) |
| Hes+SCN X Vaccine misinformation | .00 |  | (.02) |  | .00 |  | (.04) |  | .03 |  | (.02) |  | .03 | † | (.02) |
| **N** | 2314 | | |  | 2119 | | |  | 2312 | | |  | 2310 | | |
| **R^2^** | .11 | | |  | .07 | | |  | .24 | | |  | .26 | | |
| **F-test** | F(5)=.82 | | |  | F(5)=4.78*** | | |  | F(5)=2.07† | | |  | F(5)=1.07 | | |

**Notes**. † p<.10, * p<0.05, ** p<0.01, *** p<0.001.

**Figure D3.** Estimated Marginal Means Plotting for other outcome variables preregistered (See Deviations from Pre-registration for reasons for not including these results in the main report)


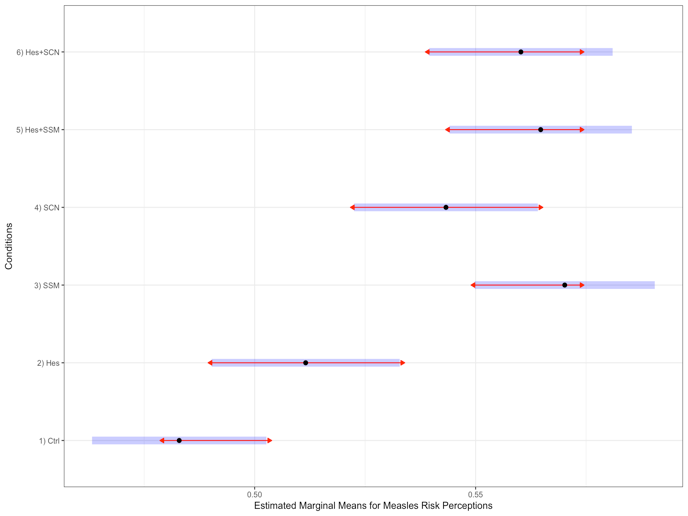

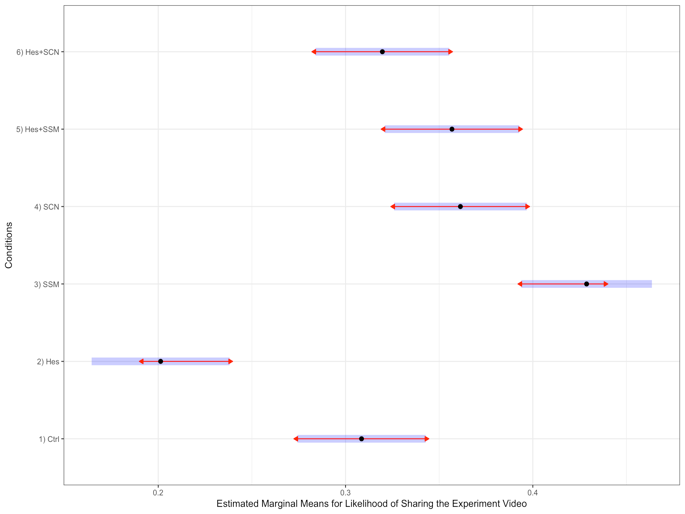


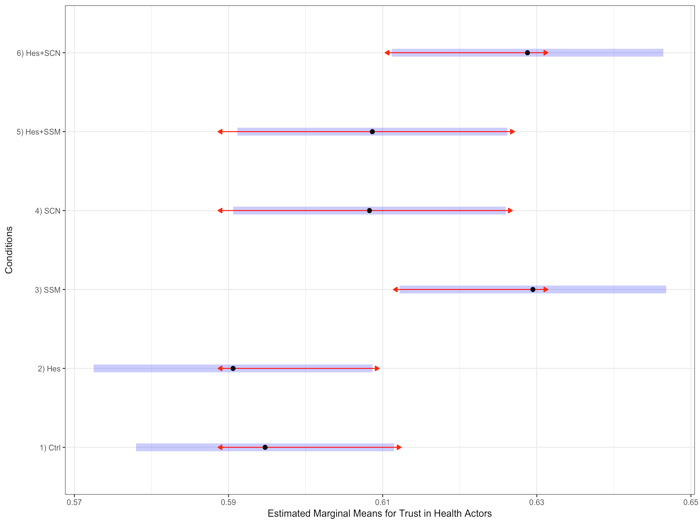

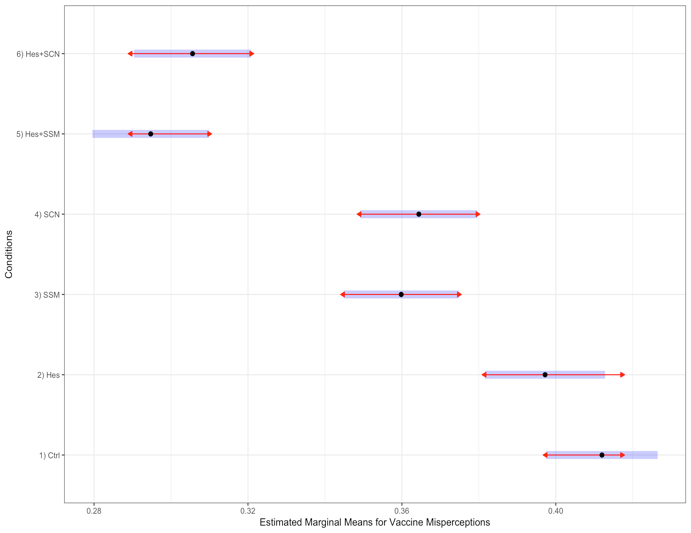


**Notes.** The four panels represent the different outcome variables indicated. Estimated (model predicted) marginal means (EMM) for each of the six conditions are shown (all outcome measures range from 0 to 1). The blue bars are the confidence intervals for EMMs while red arrows are for comparisons among them. Overlapping red arrows indicate insignificant findings between any two compared conditions accounting for the Bonferroni adjustment.

**Table D10**. Conspiratorial Belief Moderator (instead of Vaccine Misinformation)

|  | **MMR Vaccine Risk Perceptions** | | |  | **Pro-vaccine Policy Views** | | |  | **Encourage Others to Vaccinate Their Children** | | |  | **Intention to Send a Pro-vaccine Letter to State Representative** | | |
| --- | --- | --- | --- | --- | --- | --- | --- | --- | --- | --- | --- | --- | --- | --- | --- |
|  | Coef. |  | se |  | Coef. |  | se |  | Coef. |  | se |  | Coef. |  | se |
| (Intercept) | .25 | *** | (.01) |  | .77 | *** | (.02) |  | .77 | *** | (.02) |  | .76 | *** | (.17) |
| Hesitancy-Inducing Narrative | .00 |  | (.01) |  | -.01 |  | (.02) |  | .01 |  | (.02) |  | -.04 |  | (.15) |
| Science-Supporting Message (SSM) | -.03 | *** | (.01) |  | .04 | ** | (.02) |  | .06 | ** | (.02) |  | .31 | * | (.15) |
| Science-Consistent Narrative (SCN) | -.01 |  | (.01) |  | -.02 |  | (.02) |  | .01 |  | (.02) |  | -.11 |  | (.15) |
| Hesitancy + SSM | -.01 | † | (.01) |  | .02 |  | (.02) |  | .02 |  | (.02) |  | .17 |  | (.15) |
| Hesitancy + SCN | .00 |  | (.01) |  | -.01 |  | (.02) |  | .01 |  | (.02) |  | .14 |  | (.15) |
| Nonpanel Sample Respondent | .01 | * | (.00) |  | -.01 |  | (.01) |  | .00 |  | (.01) |  | -.17 | † | (.09) |
| Parent | .01 | ** | (.00) |  | -.04 | *** | (.01) |  | .00 |  | (.01) |  | -.23 | ** | (.09) |
| Media exposure to MMR | -.02 | *** | (.00) |  | .04 | *** | (.00) |  | .06 | *** | (.01) |  | .30 | *** | (.04) |
| Female | .00 |  | (.00) |  | .02 | * | (.01) |  | .06 | *** | (.01) |  | -.06 |  | (.09) |
| Conspiration | .06 | *** | (.00) |  | -.08 | *** | (.01) |  | -.11 | *** | (.01) |  | -.46 | *** | (.06) |
| **N** | 2307 | | |  | 2305 | | |  | 2297 | | |  | 2301 | | |
| **R^2^** | .28 | | |  | .14 | | |  | .14 | | |  | .07 | | |
| **Interaction Models** |  |  |  |  |  |  |  |  |  |  |  |  |  |  |  |
|  | **MMR Vaccine Risk Perceptions** | | |  | **Pro-vaccine Policy Views** | | |  | **Encourage Others to Vaccinate Their Children** | | |  | **Intention to Send a Pro-vaccine Letter to State Representative** | | |
|  | Coef. |  | se |  | Coef. |  | se |  | Coef. |  | se |  | Coef. |  | se |
| (Intercept) | .26 | *** | (.01) |  | .74 | *** | (.03) |  | .76 | *** | (.04) |  | .59 | * | (.27) |
| Hesitancy-Inducing Narrative | -.01 |  | (.02) |  | -.01 |  | (.04) |  | .03 |  | (.05) |  | .10 |  | (.38) |
| Science-Supporting Message (SSM) | -.03 |  | (.02) |  | .10 | ** | (.04) |  | .08 |  | (.05) |  | .54 |  | (.37) |
| Science-Consistent Narrative (SCN) | -.02 |  | (.02) |  | .02 |  | (.04) |  | .00 |  | (.05) |  | .04 |  | (.37) |
| Hesitancy + SSM | -.02 |  | (.02) |  | .03 |  | (.04) |  | .02 |  | (.05) |  | .31 |  | (.37) |
| Hesitancy + SCN | -.01 |  | (.02) |  | .03 |  | (.04) |  | .04 |  | (.05) |  | .48 |  | (.38) |
| Conspiration | .06 | *** | (.01) |  | -.07 | *** | (.01) |  | -.10 | *** | (.02) |  | -.37 | ** | (.13) |
| Nonpanel Sample Respondent | .01 | * | (.00) |  | -.02 |  | (.01) |  | .00 |  | (.01) |  | -.17 | † | (.09) |
| Parent | .01 | ** | (.00) |  | -.04 | *** | (.01) |  | .00 |  | (.01) |  | -.23 | ** | (.09) |
| Media exposure to MMR | -.02 | *** | (.00) |  | .04 | *** | (.00) |  | .06 | *** | (.01) |  | .30 | *** | (.04) |
| Female | .00 |  | (.00) |  | .02 | * | (.01) |  | .06 | *** | (.01) |  | -.06 |  | (.09) |
| Hes X Conspiration | .00 |  | (.01) |  | .00 |  | (.02) |  | -.01 |  | (.03) |  | -.08 |  | (.19) |
| SSM X Conspiration | .00 |  | (.01) |  | -.03 | † | (.02) |  | -.01 |  | (.03) |  | -.13 |  | (.18) |
| SCN X Conspiration | .01 |  | (.01) |  | -.02 |  | (.02) |  | .01 |  | (.03) |  | -.08 |  | (.19) |
| Hes+SSM X Conspiration | .00 |  | (.01) |  | .00 |  | (.02) |  | .00 |  | (.03) |  | -.08 |  | (.19) |
| Hes+SCN X Conspiration | .00 |  | (.01) |  | -.02 |  | (.02) |  | -.02 |  | (.03) |  | -.19 |  | (.20) |
| **N** | 2307 | | |  | 2305 | | |  | 2297 | | |  | 2301 | | |
| **R^2^** | .28 | | |  | .14 | | |  | .15 | | |  | .07 | | |
| **F-test** | F(5)=.29 | | |  | F(5)=.91 | | |  | F(5)=.24 | | |  | 1.00 | | |

**Notes.** † p<.10, * p<0.05, ** p<0.01, *** p<0.001. R^2^ for the rightmost model (Intention to Send a Pro-vaccine Letter to State Representative) is McFadden statistic. F-changes compared against the model without the interaction terms. The rightmost model (Intention to Send a Pro-vaccine Letter to State Representative) has a nonsignificant improvement in residual deviances (comparable to F-change). Conspiratorial Belief is a three item index, see preregistration for details.

**Table D11**. Longitudinal Null Effects (Time 1 model variables predicting Time 2 outcome measures)

|  | **MMR Vaccine Risk Perceptions (Longitudinal)** | | |  | **Pro-vaccine Policy Views (Longitudinal)** | | |  | **Encourage Others to Vaccinate Their Children (Longitudinal)** | | |  | **Intention to Send a Pro-vaccine Letter to State Representative (Longitudinal)** | | |
| --- | --- | --- | --- | --- | --- | --- | --- | --- | --- | --- | --- | --- | --- | --- | --- |
| ***Main effect models*** |  |  |  |  |  |  |  |  |  |  |  |  |  |  |  |
|  | **Coef.** |  | **se** |  | **Coef.** |  | **se** |  | **Coef.** |  | **se** |  | **Coef.** |  | **se** |
| (Intercept) | .06 | *** | (.01) |  | .83 |  | (.02) |  | .91 | *** | (.02) |  | 1.19 | *** | (.18) |
| Hesitancy-Inducing Narrative | .02 |  | (.01) |  | -.01 |  | (.02) |  | .01 |  | (.02) |  | -.18 |  | (.19) |
| Science-Supporting Message (SSM) | .00 |  | (.01) |  | .01 |  | (.02) |  | -.01 |  | (.02) |  | .23 |  | (.20) |
| Science-Consistent Narrative (SCN) | .01 |  | (.01) |  | -.02 |  | (.02) |  | -.01 |  | (.02) |  | -.21 |  | (.19) |
| Hesitancy + SSM | -.02 |  | (.01) |  | .00 |  | (.02) |  | .01 |  | (.02) |  | .03 |  | (.20) |
| Hesitancy + SCN | .00 |  | (.01) |  | -.02 |  | (.02) |  | -.01 |  | (.02) |  | -.21 |  | (.19) |
| Parent | .02 | ** | (.01) |  | -.01 |  | (.01) |  | .02 | † | (.01) |  | .07 |  | (.11) |
| Media exposure to MMR | -.01 | † | (.00) |  | .02 | ** | (.01) |  | .02 | * | (.01) |  | .16 | ** | (.06) |
| Female | .00 |  | (.01) |  | .00 |  | (.01) |  | .02 |  | (.01) |  | -.18 |  | (.11) |
| Vaccine misinformation | .17 | *** | (.01) |  | -.19 | *** | (.01) |  | -.20 | *** | (.01) |  | -.90 | *** | (.09) |
| **N** | 1567 | | |  | 1561 | | |  | 1571 | | |  | 1568 | | |
| **R^2^** | .36 | | |  | .26 | | |  | .22 | | |  | .23 | | |
| ***Interaction models*** |  |  |  |  |  |  |  |  |  |  |  |  |  |  |  |
| (Intercept) | .04 | ** | (.02) |  | .82 | *** | (.02) |  | .92 | *** | (.02) |  | 1.12 | *** | (.23) |
| Hesitancy-Inducing Narrative | .04 | * | (.02) |  | -.02 |  | (.02) |  | -.01 |  | (.03) |  | -.31 |  | (.27) |
| Science-Supporting Message (SSM) | .02 |  | (.02) |  | .00 |  | (.02) |  | -.02 |  | (.03) |  | .29 |  | (.29) |
| Science-Consistent Narrative (SCN) | .00 |  | (.02) |  | .00 |  | (.02) |  | .00 |  | (.03) |  | -.03 |  | (.29) |
| Hesitancy + SSM | .00 |  | (.02) |  | .02 |  | (.03) |  | .01 |  | (.03) |  | .04 |  | (.29) |
| Hesitancy + SCN | .01 |  | (.02) |  | -.01 |  | (.03) |  | -.03 |  | (.03) |  | .10 |  | (.29) |
| Vaccine misinformation | .19 | *** | (.02) |  | -.18 | *** | (.02) |  | -.21 | *** | (.02) |  | -.80 | *** | (.23) |
| Parent | .02 | ** | (.01) |  | -.01 |  | (.01) |  | .02 | † | (.01) |  | .08 |  | (.12) |
| Media exposure to MMR | -.01 | † | (.00) |  | .02 | ** | (.01) |  | .02 | ** | (.01) |  | .16 | ** | (.06) |
| Female | .01 |  | (.01) |  | .00 |  | (.01) |  | .02 |  | (.01) |  | -.18 |  | (.11) |
| Hes X Vaccine misinformation | -.04 | † | (.02) |  | .01 |  | (.03) |  | .03 |  | (.03) |  | .22 |  | (.31) |
| SSM X Vaccine misinformation | -.04 | † | (.02) |  | .01 |  | (.03) |  | .03 |  | (.03) |  | -.10 |  | (.30) |
| SCN X Vaccine misinformation | .01 |  | (.02) |  | -.04 |  | (.03) |  | -.01 |  | (.03) |  | -.28 |  | (.32) |
| Hes+SSM X Vaccine misinformation | -.03 |  | (.02) |  | -.03 |  | (.03) |  | -.01 |  | (.03) |  | -.02 |  | (.32) |
| Hes+SCN X Vaccine misinformation | -.02 |  | (.02) |  | -.02 |  | (.03) |  | .03 |  | (.03) |  | -.48 |  | (.33) |
| **N** | 1567 | | |  | 1561 | | |  | 1571 | | |  | 1568 | | |
| **R^2^** | .37 | | |  | .27 | | |  | .22 | | |  | .23 | | |
| **F-test** | F(5)=1.52 | | |  | F(5)=1.22 | | |  | F(5)=.64 | | |  | 5.9 | | |

**Notes.** † p<.10, * p<0.05, ** p<0.01, *** p<0.001. R^2^ for the rightmost model (Intention to Send a Pro-vaccine Letter to State Representative) is McFadden statistic. F-changes compared against the model without the interaction terms. The rightmost model (Intention to Send a Pro-vaccine Letter to State Representative) has a nonsignificant improvement in residual deviances (comparable to F-change). Conspiratorial Belief is a three item index.

**Figure D4**. Longitudinal Marginal Mean Effects (Time 1 model variables predicting Time 2 outcome measures)


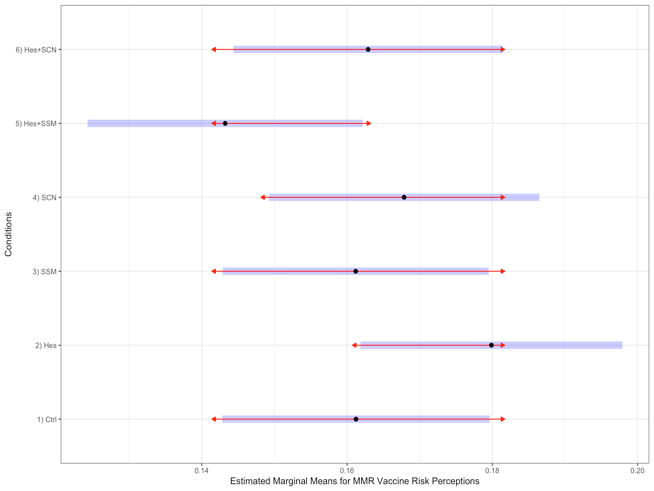

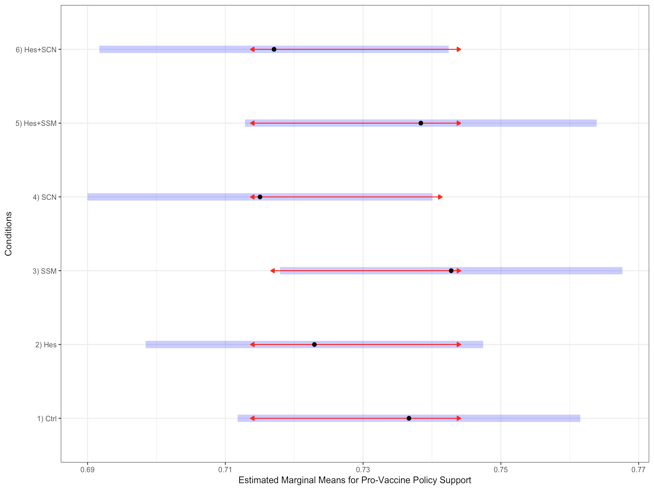


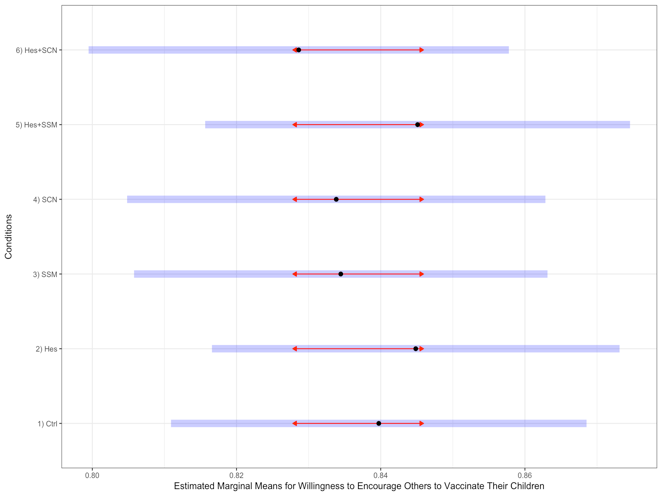

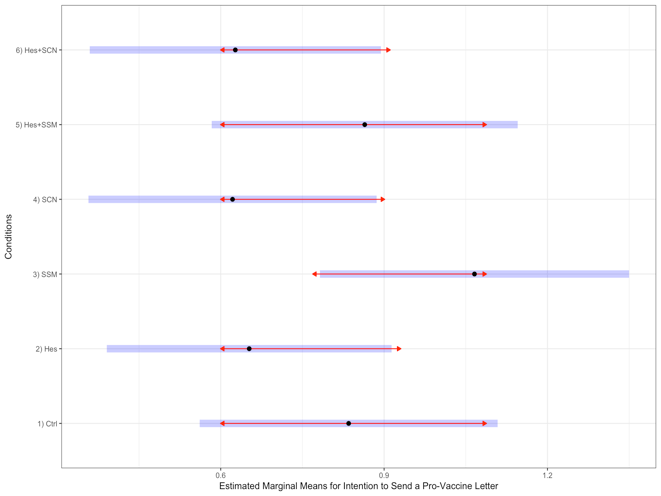


**Notes.** The four panels represent the different outcome variables indicated. Estimated (model predicted) marginal means (EMM) for each of the six conditions are shown (all outcome measures range from 0 to 1). The blue bars are the confidence intervals for EMMs while red arrows are for comparisons among them. Overlapping red arrows indicate insignificant findings between any two compared conditions accounting for the Bonferroni adjustment.

**Supporting Information F.** Additional Preregistered Analyses and Robustness Checks

***Subsection 1:*** Other Outcome Measures Pre-registered

*Please read the Deviations from Preregistration section for details on why these were not included in the main report.

Table F1.

| Dependent Variable: Measles Risk Perceptions |  |  |  |  |  |  |
| --- | --- | --- | --- | --- | --- | --- |
|  | **SS (Type III)** | ***df*** | **MS** | ***F*** | ***P*** | ***η_p_^2^*** |
| Parent | .19 | 1 | .19 | 4.46 | .04 | .002 |
| Female | 2.42 | 1 | 2.42 | 58.41 | .00 | .025 |
| Control Sample | .00 | 1 | .00 | .04 | .85 | .000 |
| Trust in Medical Authorities | .29 | 1 | .29 | 6.92 | .01 | .003 |
| Vaccine Misinformation | .86 | 1 | .86 | 20.69 | .00 | .009 |
| Media Exposure (Measles) | 2.65 | 1 | 2.65 | 63.97 | .00 | .027 |
| Hesitancy-Inducing Message (Factor 1) | .11 | 1 | .11 | 2.56 | .11 | .001 |
| Science-Supporting Message (Factor 2) | 2.06 | 2 | 1.03 | 24.88 | .00 | .021 |
| Factor 1 X Factor 2 | .11 | 2 | .05 | 1.32 | .27 | .001 |
| Residuals | 95.19 | 2300 | .04 |  |  |  |
| R^2^ | .07 |  |  |  |  |  |

Table F2.

| Dependent Variable: Likelihood of Sharing the Experimental Video |  |  |  |  |  |  |
| --- | --- | --- | --- | --- | --- | --- |
|  | **SS (Type III)** | ***df*** | **MS** | ***F*** | ***P*** | ***η_p_^2^*** |
| Parent | 11.61 | 1 | 11.61 | 11.47 | .00 | .005 |
| Female | 25.26 | 1 | 25.26 | 24.94 | .00 | .012 |
| Control Sample | .08 | 1 | .08 | .08 | .78 | .000 |
| Trust in Medical Authorities | 9.31 | 1 | 9.31 | 9.19 | .00 | .004 |
| Vaccine Misinformation | 8.51 | 1 | 8.51 | 8.41 | .00 | .004 |
| Media Exposure (Measles) | 1.78 | 1 | 1.78 | 1.75 | .19 | .001 |
| Hesitancy-Inducing Message (Factor 1) | 25.23 | 1 | 25.23 | 24.92 | .00 | .012 |
| Science-Supporting Message (Factor 2) | 60.13 | 2 | 30.07 | 29.69 | .00 | .027 |
| Factor 1 X Factor 2 | 3.38 | 2 | 1.69 | 1.67 | .19 | .002 |
| Residuals | 2131.92 | 2105 | 1.01 |  |  |  |
| R^2^ | .06 |  |  |  |  |  |

Table F3.

| Dependent Variable: Trust in Authorities Managing Public Health |  |  |  |  |  |  |
| --- | --- | --- | --- | --- | --- | --- |
|  | **SS (Type III)** | ***df*** | **MS** | ***F*** | ***P*** | ***η_p_^2^*** |
| Parent | .01 | 1 | .01 | .04 | .83 | .000 |
| Female | .02 | 1 | .02 | .11 | .74 | .000 |
| Control Sample | .14 | 1 | .14 | .78 | .38 | .000 |
| Trust in Medical Authorities | 208.10 | 1 | 208.10 | 1167.10 | .00 | .337 |
| Vaccine Misinformation | 7.15 | 1 | 7.15 | 40.11 | .00 | .017 |
| Media Exposure (Measles) | .00 | 1 | .00 | .02 | .89 | .000 |
| Hesitancy-Inducing Message (Factor 1) | .03 | 1 | .03 | .18 | .67 | .000 |
| Science-Supporting Message (Factor 2) | 2.58 | 2 | 1.29 | 7.24 | .00 | .006 |
| Factor 1 X Factor 2 | 1.33 | 2 | .66 | 3.72 | .02 | .003 |
| Residuals | 409.93 | 2299 | .18 |  |  |  |
| R^2^ | .50 |  |  |  |  |  |

Table F4.

| Dependent Variable: Vaccine Misperceptions |  |  |  |  |  |  |
| --- | --- | --- | --- | --- | --- | --- |
|  | **SS (Type III)** | ***df*** | **MS** | ***F*** | ***P*** | ***η_p_^2^*** |
| Parent | .15 | 1 | .15 | .76 | .38 | .000 |
| Female | .71 | 1 | .71 | 3.62 | .06 | .002 |
| Control Sample | 2.14 | 1 | 2.14 | 10.89 | .00 | .005 |
| Trust in Medical Authorities | 9.03 | 1 | 9.03 | 45.87 | .00 | .020 |
| Vaccine Misinformation | 36.28 | 1 | 36.28 | 184.26 | .00 | .074 |
| Media Exposure (Measles) | 8.87 | 1 | 8.87 | 45.04 | .00 | .019 |
| Hesitancy-Inducing Message (Factor 1) | 10.97 | 1 | 10.97 | 55.71 | .00 | .024 |
| Science-Supporting Message (Factor 2) | 24.76 | 2 | 12.38 | 62.87 | .00 | .052 |
| Factor 1 X Factor 2 | 2.54 | 2 | 1.27 | 6.44 | .00 | .006 |
| Residuals | 451.99 | 2296 | .20 |  |  |  |
| R^2^ | .27 |  |  |  |  |  |

***Subsection 2:*** Vaccine Misinformation Moderation

Table F5.

| Dependent Variable:  MMR Vaccine Risk Perception among Low Vaccine Misinformation Individuals |  |  |  |  |  |  |
| --- | --- | --- | --- | --- | --- | --- |
|  | **SS (Type III)** | ***df*** | **MS** | ***F*** | ***P*** | ***η_p_^2^*** |
| Parent | .57 | 1 | .57 | 4.99 | .03 | .004 |
| Female | .05 | 1 | .05 | .46 | .50 | .000 |
| Control Sample | .06 | 1 | .06 | .53 | .47 | .000 |
| Trust in Medical Authorities | 15.87 | 1 | 15.87 | 138.36 | .00 | .095 |
| Vaccine Misinformation | 6.79 | 1 | 6.79 | 59.19 | .00 | .043 |
| Media Exposure (Measles) | 3.23 | 1 | 3.23 | 28.18 | .00 | .021 |
| Hesitancy-Inducing Message (Factor 1) | .64 | 1 | .64 | 5.55 | .02 | .004 |
| Science-Supporting Message (Factor 2) | 2.08 | 2 | 1.04 | 9.05 | .00 | .014 |
| Factor 1 X Factor 2 | .55 | 2 | .27 | 2.39 | .09 | .004 |
| Residuals | 151.26 | 1319 | .12 |  |  |  |
| R^2^ | .22 |  |  |  |  |  |

**Note.** Vaccine Misinformation Low is composed of respondents who scored close to the median, at or lower than .50 in the vaccine misinformation index.

Table F6.

| Dependent Variable:  MMR Vaccine Risk Perception among High Vaccine Misinformation Individuals |  |  |  |  |  |  |
| --- | --- | --- | --- | --- | --- | --- |
|  | **SS (Type III)** | ***df*** | **MS** | ***F*** | ***P*** | ***η_p_^2^*** |
| Parent | .39 | 1 | .39 | 1.30 | .25 | .001 |
| Female | .02 | 1 | .02 | .07 | .79 | .000 |
| Control Sample | .56 | 1 | .56 | 1.88 | .17 | .002 |
| Trust in Medical Authorities | 46.63 | 1 | 46.63 | 156.39 | .00 | .141 |
| Vaccine Misinformation | 46.86 | 1 | 46.86 | 157.18 | .00 | .142 |
| Media Exposure (Measles) | 4.83 | 1 | 4.83 | 16.20 | .00 | .017 |
| Hesitancy-Inducing Message (Factor 1) | .08 | 1 | .08 | .25 | .62 | .000 |
| Science-Supporting Message (Factor 2) | 5.73 | 2 | 2.86 | 9.61 | .00 | .020 |
| Factor 1 X Factor 2 | .88 | 2 | .44 | 1.47 | .23 | .003 |
| Residuals | 282.94 | 949 | .30 |  |  |  |
| R^2^ | .40 |  |  |  |  |  |

**Note.** Vaccine Misinformation High is composed of respondents who scored close to the median, at or greater than .51 in the vaccine misinformation index.

Table F7.

| Dependent Variable: Pro-Vaccine Policy Views among Low Vaccine Misinformation Individuals |  |  |  |  |  |  |
| --- | --- | --- | --- | --- | --- | --- |
|  | **SS (Type III)** | ***df*** | **MS** | ***F*** | ***P*** | ***η_p_^2^*** |
| Parent | 2.27 | 1 | 2.27 | 4.76 | .03 | .004 |
| Female | 1.65 | 1 | 1.65 | 3.44 | .06 | .003 |
| Control Sample | .74 | 1 | .74 | 1.54 | .21 | .001 |
| Trust in Medical Authorities | 7.38 | 1 | 7.38 | 15.43 | .00 | .012 |
| Vaccine Misinformation | 41.46 | 1 | 41.46 | 86.71 | .00 | .062 |
| Media Exposure (Measles) | 3.32 | 1 | 3.32 | 6.94 | .01 | .005 |
| Ideology (Liberal) | 10.33 | 1 | 10.33 | 21.61 | .00 | .016 |
| Hesitancy-Inducing Message (Factor 1) | 1.82 | 1 | 1.82 | 3.81 | .05 | .003 |
| Science-Supporting Message (Factor 2) | 7.80 | 2 | 3.90 | 8.16 | .00 | .012 |
| Factor 1 X Factor 2 | 1.58 | 2 | .79 | 1.65 | .19 | .003 |
| Residuals | 629.75 | 1317 | .48 |  |  |  |
| R^2^ | .16 |  |  |  |  |  |

**Note.** Vaccine Misinformation Low is composed of respondents who scored close to the median, at or lower than .50 in the vaccine misinformation index.

Table F8.

| Dependent Variable: Pro-Vaccine Policy Views among High Vaccine Misinformation Individuals |  |  |  |  |  |  |
| --- | --- | --- | --- | --- | --- | --- |
|  | **SS (Type III)** | ***df*** | **MS** | ***F*** | ***P*** | ***η_p_^2^*** |
| Parent | .01 | 1 | .01 | .01 | .92 | .000 |
| Female | 2.11 | 1 | 2.11 | 2.91 | .09 | .003 |
| Control Sample | .02 | 1 | .02 | .03 | .86 | .000 |
| Trust in Medical Authorities | 33.23 | 1 | 33.23 | 45.72 | .00 | .046 |
| Vaccine Misinformation | 59.70 | 1 | 59.70 | 82.14 | .00 | .079 |
| Media Exposure (Measles) | 10.17 | 1 | 10.17 | 13.99 | .00 | .014 |
| Ideology (Liberal) | 10.80 | 1 | 10.80 | 14.85 | .00 | .015 |
| Hesitancy-Inducing Message (Factor 1) | .17 | 1 | .17 | .24 | .63 | .000 |
| Science-Supporting Message (Factor 2) | 10.82 | 2 | 5.41 | 7.44 | .00 | .015 |
| Factor 1 X Factor 2 | 2.74 | 2 | 1.37 | 1.89 | .15 | .004 |
| Residuals | 694.10 | 955 | .73 |  |  |  |
| R^2^ | .24 |  |  |  |  |  |

**Note.** Vaccine Misinformation High is composed of respondents who scored close to the median, at or greater than .51 in the vaccine misinformation index.

Table F9.

| Outcome variable: Encouraging Others to Vaccinate Their Children - among Low Vaccine Misinformation Individuals | Coef. | se | z | p value |  | OR | CI 2.5 % | CI 97.5 % |
| --- | --- | --- | --- | --- | --- | --- | --- | --- |
| Vaccine Misinformation | -2.04 | .31 | -6.51 | .00 |  | .13 | .07 | .24 |
| Control Sample | -.01 | .13 | -.09 | .93 |  | .99 | .76 | 1.29 |
| Parent | -.09 | .13 | -.74 | .46 |  | .91 | .71 | 1.17 |
| Media Exposure (Measles) | .28 | .06 | 4.61 | .00 |  | 1.32 | 1.18 | 1.49 |
| Female | .28 | .12 | 2.29 | .02 |  | 1.33 | 1.04 | 1.70 |
| Trust in Medical Authorities | .80 | .13 | 6.20 | .00 |  | 2.22 | 1.73 | 2.86 |
| Hesitancy Inducing Narrative (Hes) | -.05 | .21 | -.23 | .82 |  | .95 | .63 | 1.44 |
| Science-supporting Message (SSM) | .34 | .21 | 1.63 | .10 |  | 1.41 | .93 | 2.14 |
| Science-consistent Narrative (SCN) | .10 | .21 | .46 | .64 |  | 1.10 | .73 | 1.66 |
| Hes X SSM | -.16 | .30 | -.53 | .59 |  | .85 | .47 | 1.54 |
| Hes X SCN | .06 | .30 | .22 | .83 |  | 1.07 | .59 | 1.92 |
| Pseudo R^2^ | .11 |  |  |  |  |  |  |  |

**Note.** Vaccine Misinformation Low is composed of respondents who scored close to the median, at or lower than .50 in the vaccine misinformation index.

Table F10.

| Outcome variable: Encouraging Others to Vaccinate Their Children - among High Vaccine Misinformation Individuals | Coef. | se | z | p value |  | OR | CI 2.5 % | CI 97.5 % |
| --- | --- | --- | --- | --- | --- | --- | --- | --- |
| Vaccine Misinformation | -1.02 | .23 | -4.47 | .00 |  | .36 | .23 | .56 |
| Control Sample | -.06 | .18 | -.35 | .72 |  | .94 | .67 | 1.32 |
| Parent | .01 | .17 | .07 | .95 |  | 1.01 | .73 | 1.40 |
| Media Exposure (Measles) | .30 | .08 | 3.68 | .00 |  | 1.35 | 1.15 | 1.59 |
| Female | .74 | .17 | 4.31 | .00 |  | 2.10 | 1.50 | 2.95 |
| Trust in Medical Authorities | .99 | .15 | 6.46 | .00 |  | 2.68 | 2.00 | 3.64 |
| Hesitancy Inducing Narrative (Hes) | .20 | .29 | .69 | .49 |  | 1.22 | .69 | 2.16 |
| Science-supporting Message (SSM) | .71 | .27 | 2.59 | .01 |  | 2.03 | 1.19 | 3.47 |
| Science-consistent Narrative (SCN) | -.33 | .30 | -1.08 | .28 |  | .72 | .40 | 1.30 |
| Hes X SSM | -.99 | .40 | -2.46 | .01 |  | .37 | .17 | .81 |
| Hes X SCN | .20 | .42 | .48 | .63 |  | 1.23 | .54 | 2.80 |
| Pseudo R^2^ | .17 |  |  |  |  |  |  |  |

**Note.** Vaccine Misinformation High is composed of respondents who scored close to the median, at or greater than .51 in the vaccine misinformation index.

Table F11.

| Outcome variable: Likelihood of Agreeing to Send a Pro-Vaccine Letter to State Representative - among Low Vaccine Misinformation Individuals | Coef. | se | z | p value |  | OR | CI 2.5 % | CI 97.5 % |
| --- | --- | --- | --- | --- | --- | --- | --- | --- |
| Vaccine Misinformation | -1.94 | .32 | -6.08 | .00 |  | .14 | .08 | .27 |
| Control Sample | -.09 | .14 | -.65 | .51 |  | .92 | .70 | 1.20 |
| Parent | -.15 | .13 | -1.14 | .26 |  | .86 | .67 | 1.11 |
| Media Exposure (Measles) | .22 | .06 | 3.49 | .00 |  | 1.24 | 1.10 | 1.40 |
| Female | -.07 | .13 | -.52 | .61 |  | .94 | .73 | 1.20 |
| Trust in Medical Authorities | .12 | .13 | .97 | .33 |  | 1.13 | .88 | 1.45 |
| Ideology (Liberal) | .22 | .06 | 3.80 | .00 |  | 1.25 | 1.11 | 1.40 |
| Hesitancy Inducing Narrative (Hes) | -.17 | .21 | -.81 | .42 |  | .84 | .56 | 1.27 |
| Science-supporting Message (SSM) | .44 | .22 | 2.01 | .04 |  | 1.55 | 1.01 | 2.39 |
| Science-consistent Narrative (SCN) | -.11 | .21 | -.55 | .58 |  | .89 | .59 | 1.34 |
| Hes X SSM | .02 | .31 | .06 | .95 |  | 1.02 | .55 | 1.87 |
| Hes X SCN | .49 | .30 | 1.62 | .11 |  | 1.63 | .90 | 2.94 |
| Pseudo R^2^ | .09 |  |  |  |  |  |  |  |

**Note.** Vaccine Misinformation Low is composed of respondents who scored close to the median, at or lower than .50 in the vaccine misinformation index.

Table F12.

| Outcome variable: Likelihood of Agreeing to Send a Pro-Vaccine Letter to State Representative - among High Vaccine Misinformation Individuals | Coef. | se | z | p value |  | OR | CI 2.5 % | CI 97.5 % |
| --- | --- | --- | --- | --- | --- | --- | --- | --- |
| Vaccine Misinformation | -.52 | .16 | -3.16 | .00 |  | .60 | .43 | .82 |
| Control Sample | -.25 | .15 | -1.71 | .09 |  | .78 | .58 | 1.04 |
| Parent | -.05 | .14 | -.35 | .73 |  | .95 | .72 | 1.25 |
| Media Exposure (Measles) | .13 | .07 | 1.91 | .06 |  | 1.14 | 1.00 | 1.31 |
| Female | -.12 | .14 | -.83 | .41 |  | .89 | .67 | 1.17 |
| Trust in Medical Authorities | .57 | .11 | 5.00 | .00 |  | 1.76 | 1.42 | 2.21 |
| Ideology (Liberal) | .19 | .07 | 2.86 | .00 |  | 1.21 | 1.06 | 1.38 |
| Hesitancy Inducing Narrative (Hes) | -.15 | .24 | -.63 | .53 |  | .86 | .53 | 1.38 |
| Science-supporting Message (SSM) | .29 | .23 | 1.24 | .21 |  | 1.33 | .85 | 2.11 |
| Science-consistent Narrative (SCN) | -.24 | .24 | -.99 | .32 |  | .79 | .49 | 1.26 |
| Hes X SSM | -.05 | .34 | -.15 | .88 |  | .95 | .49 | 1.85 |
| Hes X SCN | .48 | .35 | 1.39 | .17 |  | 1.62 | .82 | 3.22 |
| Pseudo R^2^ | .08 |  |  |  |  |  |  |  |

**Note.** Vaccine Misinformation High is composed of respondents who scored close to the median, at or greater than .51 in the vaccine misinformation index.

***Subsection 3:*** Device of Survey-taking (PC vs phone/tablet)

Table F13.

| Dependent Variable: MMR Vaccine Risk Perceptions |  |  |  |  |  |  |
| --- | --- | --- | --- | --- | --- | --- |
|  | **SS (Type III)** | ***df*** | **MS** | ***F*** | ***P*** | ***η_p_^2^*** |
| Parent | .37 | 1 | .37 | 1.91 | .17 | .001 |
| Female | .02 | 1 | .02 | .08 | .77 | .000 |
| Control Sample | .43 | 1 | .43 | 2.21 | .14 | .001 |
| Trust in Medical Authorities | 59.75 | 1 | 59.75 | 310.63 | .00 | .120 |
| Vaccine Misinformation | 140.27 | 1 | 140.27 | 729.18 | .00 | .242 |
| Media Exposure (Measles) | 6.82 | 1 | 6.82 | 35.43 | .00 | .015 |
| Device type | .86 | 1 | .86 | 4.46 | .04 | .002 |
| Hesitancy-Inducing Message (Factor 1) | .64 | 1 | .64 | 3.30 | .07 | .001 |
| Science-Supporting Message (Factor 2) | 7.24 | 2 | 3.62 | 18.82 | .00 | .016 |
| Factor 1 X Factor 2 | 1.14 | 2 | .57 | 2.96 | .05 | .003 |
| Residuals | 438.39 | 2279 | .19 |  |  |  |
| R^2^ | .55 |  |  |  |  |  |

Table F14.

| Dependent Variable: Pro-Vaccine Policy Views |  |  |  |  |  |  |
| --- | --- | --- | --- | --- | --- | --- |
|  | **SS (Type III)** | ***df*** | **MS** | ***F*** | ***P*** | ***η_p_^2^*** |
| Parent | 1.67 | 1 | 1.67 | 2.86 | .09 | .001 |
| Female | 3.32 | 1 | 3.32 | 5.67 | .02 | .002 |
| Control Sample | .31 | 1 | .31 | .52 | .47 | .000 |
| Trust in Medical Authorities | 38.33 | 1 | 38.33 | 65.52 | .00 | .028 |
| Vaccine Misinformation | 276.05 | 1 | 276.05 | 471.86 | .00 | .171 |
| Media Exposure (Measles) | 12.72 | 1 | 12.72 | 21.74 | .00 | .009 |
| Ideology (Liberal) | 21.67 | 1 | 21.67 | 37.03 | .00 | .016 |
| Device type | .51 | 1 | .51 | .88 | .35 | .000 |
| Hesitancy-Inducing Message (Factor 1) | 1.50 | 1 | 1.50 | 2.57 | .11 | .001 |
| Science-Supporting Message (Factor 2) | 18.37 | 2 | 9.19 | 15.70 | .00 | .014 |
| Factor 1 X Factor 2 | 3.78 | 2 | 1.89 | 3.23 | .04 | .003 |
| Residuals | 1336.20 | 2284 | .59 |  |  |  |
| R^2^ | .39 |  |  |  |  |  |

Table F15.

| Outcome variable: Encouraging Others to Vaccinate Their Children | Coef. | se | z value | | p | |  | | OR | | CI 2.5% | | CI 97.5% | |
| --- | --- | --- | --- | --- | --- | --- | --- | --- | --- | --- | --- | --- | --- | --- |
| Vaccine Misinformation | -1.37 | (.11) | -12.75 | .00 | |  | | .25 | | .21 | | .31 | |  |
| Control Sample | -.02 | (.11) | -.24 | .81 | |  | | .98 | | .79 | | 1.20 | |  |
| Parent | -.08 | (.10) | -.82 | .42 | |  | | .92 | | .75 | | 1.12 | |  |
| Media Exposure (Measles) | .29 | (.05) | 5.93 | .00 | |  | | 1.34 | | 1.21 | | 1.47 | |  |
| Female | .43 | (.10) | 4.31 | .00 | |  | | 1.54 | | 1.27 | | 1.87 | |  |
| Trust in Medical Authorities | .86 | (.10) | 8.79 | .00 | |  | | 2.36 | | 1.95 | | 2.87 | |  |
| Phone | .01 | (.10) | .11 | .91 | |  | | 1.01 | | .83 | | 1.24 | |  |
| Hesitancy Inducing Narrative (Hes) | .07 | (.17) | .41 | .68 | |  | | 1.07 | | .77 | | 1.49 | |  |
| Science-supporting Message (SSM) | .52 | (.17) | 3.10 | .00 | |  | | 1.68 | | 1.21 | | 2.34 | |  |
| Science-consistent Narrative (SCN) | -.02 | (.17) | -.11 | .91 | |  | | .98 | | .71 | | 1.36 | |  |
| Hes X SSM | -.49 | (.24) | -2.03 | .04 | |  | | .61 | | .38 | | .98 | |  |
| Hes X SCN | .08 | (.24) | .35 | .73 | |  | | 1.09 | | .68 | | 1.74 | |  |
| Pseudo R^2^ | .23 |  |  |  | |  | |  | |  | |  | |  |

Table F16.

| Outcome variable: Likelihood of Agreeing to Send a Pro-Vaccine Letter to State Representative | Coef. | se | z value | p |  | OR | CI 2.5% | CI 97.5% |
| --- | --- | --- | --- | --- | --- | --- | --- | --- |
| Vaccine Misinformation | -.93 | (.09) | -10.13 | .00 |  | .40 | .33 | .47 |
| Control Sample | -.13 | (.10) | -1.34 | .18 |  | .88 | .72 | 1.06 |
| Parent | -.07 | (.10) | -.77 | .44 |  | .93 | .77 | 1.12 |
| Media Exposure (Measles) | .17 | (.05) | 3.61 | .00 |  | 1.18 | 1.08 | 1.29 |
| Female | -.09 | (.09) | -.91 | .36 |  | .92 | .76 | 1.10 |
| Trust in Medical Authorities | .36 | (.08) | 4.39 | .00 |  | 1.44 | 1.22 | 1.70 |
| Ideology (Liberal) | .21 | (.04) | 4.93 | .00 |  | 1.24 | 1.14 | 1.35 |
| Phone | -.14 | (.10) | -1.50 | .13 |  | .87 | .72 | 1.05 |
| Hesitancy Inducing Narrative (Hes) | -.16 | (.16) | -1.01 | .31 |  | .85 | .62 | 1.16 |
| Science-supporting Message (SSM) | .37 | (.16) | 2.36 | .02 |  | 1.45 | 1.07 | 1.98 |
| Science-consistent Narrative (SCN) | -.16 | (.16) | -1.02 | .31 |  | .85 | .63 | 1.16 |
| Hes X SSM | .00 | (.23) | .00 | 1.00 |  | 1.00 | .64 | 1.56 |
| Hes X SCN | .48 | (.23) | 2.12 | .03 |  | 1.62 | 1.04 | 2.53 |
| Pseudo R^2^ | .15 |  |  |  |  |  |  |  |
